# Towards a General Method for Using Cyclotron-Produced Ga68 to Manufacture Clinical and Research Ga68 Tracers

**DOI:** 10.3390/molecules29225457

**Published:** 2024-11-19

**Authors:** Ivan E. Wang, Kevin Cheng, Allen F. Brooks, Peter J. H. Scott, Benjamin L. Viglianti

**Affiliations:** 1 Department of Medicinal Chemistry, College of Pharmacy, University of Michigan, 1301 Catherine St. 2276 Medical Science I, Ann Arbor, MI 48109, USA; ivanw@med.umich.edu (I.E.W.); pjhscott@med.umich.edu (P.J.H.S.); 2 Division of Nuclear Medicine, Department of Radiology, University of Michigan, 1301 Catherine St. 2276 Medical Science I, Ann Arbor, MI 48109, USA; chengkev@umich.edu (K.C.); afb@umich.edu (A.F.B.)

**Keywords:** cyclotron, gallium, pentixafor, FAPI

## Abstract

The success of multiple nuclear medicine radiotherapeutics in treating cancer requires an increased supply of companion diagnostic imaging agents radiolabeled with gallium-68. Cyclotron production addresses the need for access to gallium-68 and has been validated for use with commercially produced sterile kits. For novel research tracers undergoing translational studies (IND or RDRC), developing and purchasing sterile kits is time- and cost-prohibitive. An on-cassette labeling method with terminal filtration allows non-sterile kits to be fabricated in-house, simplifying workflow and allowing multiple PET imaging agents to be evaluated using the same kit (i.e., parts, reagents, and timelist) with minimal variation. Using modified GE gallium chloride cassettes, four diverse clinically relevant tracers (DOTA-TOC, FAPI-04, pentixafor, and PSMA-11) were radiolabeled with gallium-68 to evaluate the approach using DOTA and HBED-CC chelator types. The tracers were all formulated according to established FDA-approved formulations and sterile-filtered using a PVDF membrane. The automated procedure is robust, tolerating DOTA and HBED-CC chelators, and can be used to screen numerous gallium-68 agents for rapid translation to clinical use.

## 1. Introduction

The use of chelation chemistry to link a radiometal (for example, a diagnostic agent (gallium-68, etc.) or a therapeutic agent (lutetium-177, etc.)) to a targeting moiety (usually an antibody, small peptide, or a peptidomimetic) has been a key strategy in developing novel theranostic pairs in nuclear medicine. Since binding, pharmacokinetics, and subsequent localization in tumors are expected to be similar between theranostic pairs as the targeting moiety and its chelator are unchanged, many of the most notable theranostic pairs use this strategy. These similarities allow for a diagnostic agent to be used for initial screening and subsequent monitoring, whereas a therapeutic agent can be used for the specific targeting of tumors with limited off-target toxicity. With the increased enrollment of cancer patients in nuclear medicine treatments, increased amounts of consistent gallium-68 production are required to support the current clinical load and to evaluate and bring new PET imaging diagnostics to clinical settings. 

Although the method for using germanium-68/gallium-68 generators is robust, and its workflow is cemented by its day-to-day use in nuclear pharmacies with a kit-based approach, the decay of these generators and their limited activity make scheduling difficult and make meeting tracer demand problematic. Generator elutions can simplify workflow, with production on demand, but their low activity loading (<3.7 GBq) compared to molybdenum-99/technetium-99m generators limits supply. Cyclotron-based production provides an alternative to gallium-68 generators that is more consistent and allows for production on-site without the need for a generator (or germanium-68) supply, but it requires additional infrastructure as well as purification methods to separate metal contaminants, which can prevent the coordination of radiogallium [1,2]. The established kit-based workflow using commercially approved kits (NetSpot and Locametz) is currently incompatible with the direct addition of cyclotron-produced [^68^Ga]GaCl_3_ (using commercially available cassettes) [3]. Alternatively, suitably purified [^68^Ga]GaCl_3_ can be produced using a cyclotron for the subsequent labeling of precursors. We recently reported a method for the purification of cyclotron-produced [^68^Ga]GaCl_3_, since no commercial cassette is currently available [3,4]. However, without a regulatory certified “sterile” space or sterile instruments, kits that are prepared in-house for research purposes using our previously published method do not meet regulatory criteria. Terminal filtration is required to achieve sterile doses for injection in humans; thus, using the demonstrated purification protocol, the development of an on-cassette labeling method is required for research tracers to be used in initial investigational new drug (IND) applications or radioactive drug research committee (RDRC) studies. 

In the United States, for research tracers to be used as clinical diagnostic tracers, the FDA must approve their use. Gathering sufficient data to support their use requires initial studies to demonstrate basic information regarding tracer metabolism, kinetics, dosimetry, and localization [5]. These studies often consist of a limited number of scans (up to 30 patients), which makes the ordering of custom compound kits in an ISO-rated clean room expensive and impractical. An on-cassette labeling method that labels multiple different gallium-68-based tracers can help screen promising tracers and streamline the approval of tracers in development without sterile compounding kits. 

Recently, two gallium-68-based tracers (FAPI-04, pentixafor) were identified for use in the imaging of cancer and other diseases where expression of the relevant protein target is present. FAPI-04 is a proline–glycine peptidomimetic linked to a DOTA (dodecane tetraacetic acid) chelator that has a ~ 6.5 nM affinity for fibroblast-activating protein (FAP) [6]. FAP is overexpressed in numerous cancers and is associated with matrix remodeling, angiogenesis, chemotherapy resistance, and immunosuppression. FAPI-04 has increased tumor internalization and uptake compared to the background, providing enhanced imaging when compared to other FAPI analogs [6,7,8,9]. Since FAPI-04 also relies on chelation, therapeutic pairs using lutetium-177 or yttrium-90 have been proposed for targeted therapy. Pentixafor (boclatixafortide) is a cyclic 5-amino-acid peptide linked to a DOTA chelator that has a ~20 nM affinity for chemokine receptor 4 (CXCR4) [10]. As an analog of the endogenous ligand CXCL12 which is implicated in tumorigenesis, tumor progression, and metastasis, pentixafor has been developed to image various cancers and immune dysregulation. Currently, there are three ongoing clinical trials looking at the use of pentixafor as an alternative to current tracers for imaging lymphomas, neuroendocrine tumors (NETs), and adrenal adenomas [11,12]. The therapeutic pairing of pentixafor with Pentixather using yttrium-90 or lutetium-177 has been proposed for targeted therapy in hematologic malignancies and solid tumors (Table 1) [13,14]. Together, these tracers, FAPI-04 and pentixafor, have demonstrated the need for an increased supply of gallium-68. With an on-cassette method, initial research with new indications can be explored using an IND or RDRC pilot study without the need for commercial sterile kits. The goal is to explore common chelator types (DOTA and HBED-CC (N,N′-bis-[2-hydroxy-5-(carboxyethyl)benzyl]ethylenediamine-N,N′-diacetic acid)) with peptides and peptidomimetics (FAPI-04 and pentixafor) to determine if an on-cassette workflow using cyclotron-produced [^68^Ga]GaCl_3_ would be compatible with multiple gallium-68 radiopharmaceuticals for use in initial clinical studies. 

## 2. Results and Discussion

### 2.1. Reduced-Volume Elution of TK200, Formulation of [^68^Ga]GaCl_3_, and Labeling of DOTA-TOC

**Figure 1:**
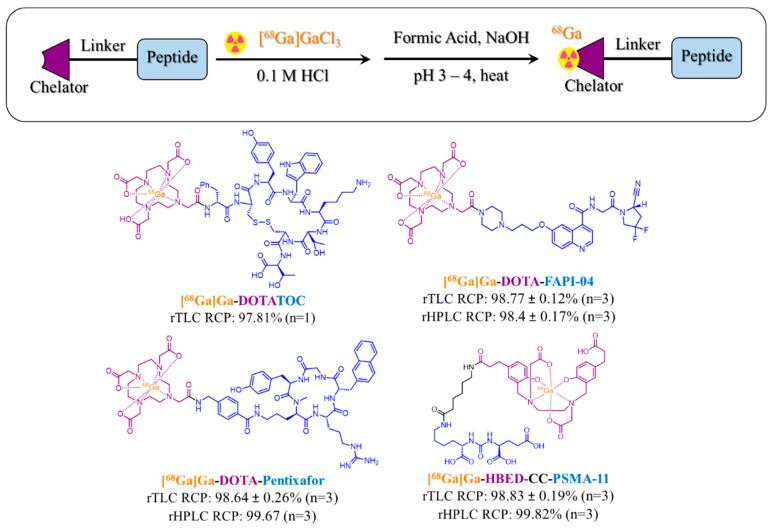
**On-cassette labeling of clinically relevant tracers.** Four clinically relevant tracers, DOTA-TOC, FAPI-04, pentixafor, and PSMA-11, were labeled with gallium-68 using the on-cassette labeling method. In orange is the radiometal, in purple is the chelator, in black is the linker, and in blue is the targeting peptide. Radiochemical purity (RCP) was determined by both radioTLC and radioHPLC.

The current GE gallium chloride cassette and the modified GE gallium chloride cassette dispense [^68^Ga]GaCl_3_ in 5 mL of 0.1 M HCl, which is comparable to a 5 mL generator eluate for the subsequent labeling of commercial kits [1,2,3,4]. A reduced [^68^Ga]GaCl_3_ volume must be used to accommodate the maximum volume of the GE reactors (4.5 mL). Currently, the elution of the TK200 resin is accomplished first with water and then with a 0.433 M HCl solution. By reducing the volumes of both elutions by half, 2.5 mL of [^68^Ga]GaCl_3_ is dispensed. Reduced TK200 elution led to 1698 MBq of [^68^Ga]GaCl_3_ in 2.6 mL, with 46.6 ± 7.2 MBq (decay-corrected) retained on the TK200 resin. The elution efficiency was 97.13 ± 0.4% (n = 2), indicating negligible activity loss and the successful reduction of [^68^Ga]GaCl_3_ volume. To test for [^68^Ga]GaCl_3_ suitability, 2.6 mL of [^68^Ga]GaCl_3_ was added to a formulated vial of DOTA-TOC, following the metal readout assay, yielding 1354 MBq in 2.6 mL with a radiochemical purity (RCP) by radio-thin layer chromatography (rTLC) of 97.81% and a pH of 3.0 to 3.3 (Figure 1). [3] This demonstrated that using reduced volumes to elute the TK200 resin still yielded suitable [^68^Ga]GaCl_3_ for the radiolabeling of gallium-68 kits. 

### 2.2. Filter Membrane Material and Filter Efficiency with DOTA-TOC

Previously, only generator or cyclotron-produced [^68^Ga]GaCl_3_ was terminally filtered [3]. Since [^68^Ga]GaCl_3_ is relatively small and inert, interaction with filter membrane material is not a major consideration. The use of small peptides with various functional groups that can interact with filter membrane material can complicate the terminal filtration process and lead to increased filter retention. Thus, various filter membrane materials were tested with DOTA-TOC. For DOTA-TOC, the Millex-GV filter retained the least amount of activity (12.38%) when compared to the Millex-LG (17.41%) and Millex-GP (23.72%) filters. The Millex-GS filter led to the highest product retention due to its construction from mixed cellulose esters which interact with DOTA-TOC. This led to a 99.67% retention of activity on the filter (Table 2). In a separate test run, labeled [^68^Ga]Ga-DOTA-TOC was pushed through a Cathivex-GV filter (PVDF) and dried using 48 mL of air, leading to 6.54% of activity being retained on the filter. This was conducted to simulate the complete drying of the filter using nitrogen push gas from a synthesis module (FASTLab 2 or related cassette-type synthesizer) while transferring into the product vial. Between the Millex-GV and Cathivex-GV filters, Cathivex-GV is preferred, as the included vents prevent air bubbles from locking the filter. The data indicate that Cathivex-GV filters were the most optimal for the terminal filtration of small peptides, which were used for the terminal filtration of FAPI-04, pentixafor, and PSMA-11. 

For DOTA-TOC, the filtered product was analyzed for RCP using rTLC. Doses produced with optimized sterile filtering all had RCP > 98%. 

### 2.3. Vial and Reactor Preparation

The well-established use of NetSpot kits in gallium-68 production has demonstrated the robustness of the formulation. Therefore, the investigated gallium tracers were formulated and assessed for compatibility with the NetSpot formulation. Precursors were formulated as previously reported; however, depending on the solubility of the precursor, variations in stock solution preparation were used to accurately measure and transfer the precursor into the reactor [3]. For highly water-soluble precursors (DOTA-TOC, pentixafor, PSMA-11), Milli-Q water was used to prepare the stock solutions. To transfer the moderately water-soluble precursor (FAPI-04), a 0.5% acetonitrile solution in MQ water was used (Table 3). The samples were lyophilized to remove the remaining solvents to yield either a free-flowing white powder or a white solid disk. Although acetonitrile is a class II solvent, to prevent an additional quality control test or periodical quality control tests, minimal volume acetonitrile was used in sample preparation. By using a lyophilizer, the solvents used to transfer the precursor were removed, indicating little need for additional solvent analysis after labeling with gallium-68 [15,16]. 

### 2.4. Labeling and Validation of Gallium Tracers

The cassette layout and setup were unchanged between gallium tracers, with only minor variations in timelist used for the heating and cooling of the reactor during chelation. Labeling with gallium-68 is dependent on the precursor (or peptide) characteristics and the chelator used (chelation chemistry and optimal pH for chelation). The use of a pH adjusting agent (or buffer) is required for an optimal chelation reaction, which for gallium-68 occurs between 3.5 and 6.5 using DOTA or HBED-CC chelators [17]. A specific temperature for heating and duration of heating are also required for consistent labeling in a time-efficient manner [17]. Cooling the reactor was necessary due to the use of plastic tubing, which can soften or deform at elevated temperatures (100 °C). 

For precursors containing the DOTA chelator (DOTA-TOC, FAPI-04, pentixafor), after the addition of 0.5 mL of buffer solution, the reactor was heated at 100 °C for 8 min, with 4 min of active cooling using compressed air [3]. The labeling of FAPI-04 using gallium-68 (n = 3) at the end of synthesis (49 min after end of bombardment) yielded 1273 ± 60.7 MBq in 2.7 mL. Often, a 1 M ammonia acetate in methanol (1:1) solvent system is used for rTLC analysis of FDA-approved peptide-based radiopharmaceuticals (DOTA-TATE, PSMA-11); however, with FAPI-04, there was poor resolution between free gallium, gallium colloids, and labeled FAPI-04 due to a broad product peak. Switching to a 5 M ammonia acetate in methanol (1:1) solvent system led to improved separation, which was adapted for in the analysis of RCP by rTLC for FAPI-04. The doses had an initial RCP by rTLC of 98.77 ± 0.12% and by rHPLC of 98.4 ± 0.17%, and at 4 h, they had an RCP by rTLC of 98.17 ± 0.53% and by rHPLC of 98.26 ± 0.05%, all of which were passing values (Figure 1 and Table 4). All doses also passed based on sterility and suitability criteria. Pentixafor, at the end of synthesis (49.2 min after end of bombardment), yielded 1288 ± 64.4 MBq in 2.7 ± 0.08 mL. Using a 1 M ammonia acetate in methanol (1:1) solvent system, the doses had an initial RCP by rTLC of 98.64 ± 0.26% and by rHPLC of 99.67%, and at 4 h, measurements were taken that showed the stability of [^68^Ga]Ga-pentixafor, an RCP by rTLC of 98.43 ± 0.19%, and by rHPLC of 99.15 ± 0.12% (Figure 1 and Table 4). All doses also passed based on sterility and suitability criteria. 

For precursors containing the HBED-CC chelator, as previously demonstrated, the timelist was modified to heat at 60 °C for 5 min [2]. Additionally, a 30 sec active cooling step was used to prevent the softening or deformation of plastic tubing during transfer. The labeling of PSMA-11 using gallium-68 was validated once and then repeated another two times without sterility testing to determine procedure robustness (total n = 3). At the end of synthesis (38.6 min), 1177 ± 31.8 MBq in 2.5 mL was produced. The doses had an initial RCP by rTLC of 98.83 ± 0.19% and by rHPLC of 99.82%, and at 4 h, they had an RCP by rTLC of 98.84 ± 0.10% and by rHPLC of 99.84%, all of which satisfy quality control metrics for use (Figure 1 and Table 4). For the one dose that was fully validated, sterility and suitability data conformed with quality control criteria. The labeling of PSMA-11 with this procedure demonstrated feasibility and the universal use of this method with minimal changes. For the production of PSMA-11, given the FDA-approved kits (Locametz or Illuccix) and the many additional approved methods, our method demonstrates the versatility of the approach and its ability to prepare gallium-68 agents that feature a HBED-CC chelator [2,18,19]. Additionally, validations using the on-cassette method were not conducted as the FDA-approved kits and the previously established cyclotron method are more optimal [2,18,19].

### 2.5. A Generalized Cyclotron Gallium-68 Labeling Method

On-cassette synthesis allows the production of numerous clinically relevant tracers with minimal changes in the synthesis timelist, precursor preparation workflow, and cassette setup. This allows the preparation of custom GE FASTLab 2 cassettes ahead of production, only requiring the addition of a formulated precursor reactor the day of synthesis, which can be prepared ahead of time and stored in a freezer. The workflow for aliquoting the precursor reactor only differs in the solvent used to prepare the precursor stock solution, which is dependent on the polarity and solubility of the tracer of interest. The minimal changes in precursor preparation workflow allow for improved consistency without outsourcing production in initial IND or RDRC studies.

The FASTLab 2 synthesizer pushes the labeled product through a 0.22 μm sterile filter for terminal filtration automatically. This reduces hand exposure to radiation when compared to the previously reported approach for manually adding [^68^Ga]GaCl_3_ across a sterile filter into a sterile formulated vial [3]. Subsequent filter integrity, endotoxin, and post-release sterility testing allow the preparation of precursor reactors in a non-sterile ISO-rated clean room, without the need for sterile instruments (lyophilizer, pipettors) or sterile assembly parts (GE COC reactor, luer lock caps, reagents for formulation, precursor) during preparation. This simplifies the workflow and cost associated with pilot production of a novel tracer, as outsourcing vial preparation is expensive at smaller scales. 

The on-cassette method is compatible with the DOTA and HBED-CC chelators. For other chelators that were not tested (NOTA, NOTP, NODAGA, DFO, THP, etc.), similar pH and heat considerations could lead to the suitable incorporation of gallium-68. This allows for the initial screening of various tracers with numerous gallium-68 chelators. Additionally, with known chelators, the pH adjustment for labeling can be altered so that alterative radiometals, including SPECT (In-111, etc.), PET (Cu-64, etc.), and beta (Lu-177, etc.) or alpha (Ac-225, etc.) emitting isotopes, can be chelated to the precursor to produce theranostic pairs. This on-cassette method can be modified with other radiometals to also allow the use of therapeutic isotopes for initial preclinical animal studies in addition to diagnostic isotopes for IND and RDRC studies. 

## 3. Materials and Methods

### 3.1. General

Isotopically enriched [^68^Zn]ZnO (≥98.2% enriched) was purchased from Isoflex USA (San Francisco, CA, cat. no. Zn-68 Oxide). Nitric Acid 70% (≥99.999% trace metal basis), 1,10 phenanthroline (≥99%), and DMSO (≥99.9%, anhydrous) were purchased from Sigma-Aldrich (Burlington, MA, USA). The 0.22 μm sterile filters (Millex-GP, Millex-GS, Millex-GV, and Cathivex-GV), 0.2 μm sterile filters (Millex-FG and Millex-LG), tryptic soy broth (TSB), and fluid thioglycolate medium (FTM) were purchased from Millipore-Sigma (Darmstadt, Germany). DOTA-(Tyr3)-octreotide acetate (DOTA-TOC, CAS: 204318-14-9), 2,5-dihydroxybenzoic acid (gentisic acid, 99%), D-mannitol (98%), formic acid (97%, ACS reagent), acetonitrile (99.9%, extra dry), and NaOH (NF/FCC analytical grade) were purchased from Fischer Scientific (Waltham, MA, USA). DOTA-FAPI-04 (CAS: 2374782-02-0) was purchased from MedChemExpress (Monmouth Junction, NJ, USA). DOTA-(Tyr3)-pentixafor (CAS: 1341207-62-2) and HBED-CC-PSMA-11 (CAS: 1366302-52-4) were purchased from ABX pharmaceuticals (Advanced Biochemical Compounds, Radeberg, Germany). Absolute (200 proof) ethanol (anhydrous, USP) was purchased from Decon Laboratories, Inc. (King of Prussia, PA, USA). Vials with natural PTFE/blue silicone septa were purchased from Wheaton (Millville, NJ, USA). The 10 mL empty sterile vials were purchased from Hollister Stier (Spokane, WA, USA). Sterile ultrapure 0.1 M HCl and a 1.85 GBq germanium-68/gallium-68 generator (GalliaPharm) were purchased from Eckert & Ziegler (Berlin, Germany). All solutions and dilutions were prepared using Milli-Q (Millipore, MQ (Darmstadt, Germany)) water with a Q-Gard 2 filter unless otherwise indicated. The 100 mL sterile water (sterile water for injection, SWFI) bags, cyclic olefin copolymer (COC) material reactors, developer kits, and gallium chloride cassettes were purchased from GE Healthcare (Chicago, IL, USA). ZR resins was purchased from Triskem International (Bruz, Bretagne, France). Glass microfiber radio-thin layer chromatographs (rTLC) impregnated with silica acid were purchased from Agilent Technologies (Santa Clara, CA, USA).

### 3.2. Liquid Target Preparation, Cyclotron Target Irradiation, and Gallium-68 Purification

Dissolution of isotopically enriched [^68^Zn]ZnO in nitric acid to prepare zinc-68 liquid targets, cyclotron target irradiation, and gallium-68 purification were conducted as previously reported [3]. In brief, 2.5 mL of Zn(NO_3_)_2_ solution was irradiated at 50 μA for 60 min using a GE PETtrace 800 cyclotron producing approximately 6.1 GBq of [^68^Ga]Ga(NO_3_)_3_ at the end of irradiation [3]. The target solution containing [^68^Ga]Ga(NO_3_)_3_, co-produced nitrogen-13 species, and other metal contaminants was delivered to a FASTLab 2 synthesizer and passed through an in-line C18 resin to remove residuals (trace quantities) of organic impurities. Subsequently, a 2 mL (340 mg) hydroxamate resin (ZR Load) was used for the initial trapping of gallium-68 [3]. After sufficient washing to remove impurities, the gallium-68 was fractionally eluted across two 2 mL (680 mg) hydroxamate (ZR chromatography) resins and formulated using a strong anion exchange (SAX) A8 resin and a trioctyl phosphine-based TK200 resin to yield purified [^68^Ga]GaCl_3_ suitable for kit labeling. To elute the TK200 resin, approximately 2.18 mL SWFI and then 3.03 mL of 0.433 M HCl were used. Reduced-volume TK200 elution was also used, containing approximately 1.09 mL SWFI and then 1.52 mL of 0.433 M HCl. This led to a formulated [^68^Ga]GaCl_3_ solution of ~0.1 M in 2.5 mL instead of the original 5 mL. It is worth noting that the volumes and molarities were approximated from GE’s gallium chloride timelist.

### 3.3. Formulation of Precursor in Vial and Reactor 

Precursor was formulated to match the NetSpot (DOTA-TATE) kit formulation in GE COC reactors or vials [20]. A stock solution of 1,10 phenanthroline was prepared at a concentration of 200 μg/mL in 200 proof ethanol, 25 μL of which was added to each reactor or vial. The reactors or vials were dried under vacuum (at a minimum of 2 h). Then, 2 mg/mL gentisic acid and 25 mg/mL **D**-mannitol stock solutions were prepared in Milli-Q (MQ) water, 3 μL and 800 μL of which were added to each reactor or vial. Finally, the precursor dissolved in MQ water (DOTA-TOC, pentixafor, PSMA-11) or a 0.5% solution of acetonitrile in water (FAPI-04) was used to transfer the stock precursor solution into each reactor or vial (see Table 2). The formulated reactors or vials were placed in a freezer (−20 °C) for at minimum 2 h until frozen and then lyophilized for 3–4 days until a dry powder was observed. This led to the formulation of 5 μg 1,10 phenanthroline, 6 μg gentisic acid, 20 mg **D**-mannitol, and 28–32 nmol of precursor per reactor or vial. These reactors or vials were kept in a freezer (−20 °C) prior to use.

The reaction buffer was also modeled after the NetSpot (DOTA-TATE) kit formulation, which was made in 10 mL batches by dissolving 565 mg sodium hydroxide and 600 mg (490 μL) formic acid in 9.51 mL of MQ water. For each reaction, approximately 0.5 to 0.55 mL of buffer was used. 

### 3.4. Development of a On-Cassette Layout

**Figure 2:**
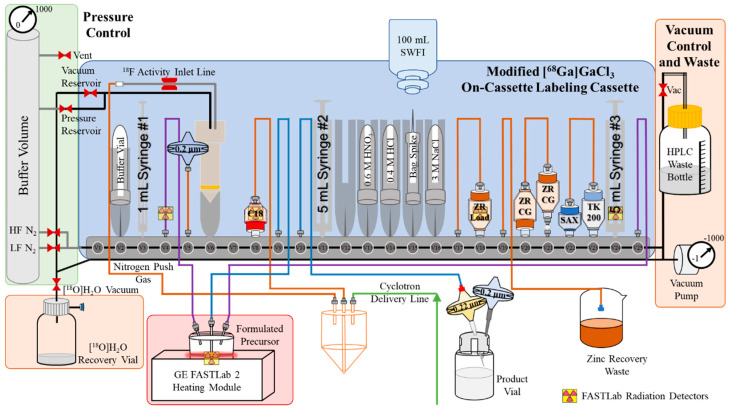
**FASTLab 2 cassette layout for on-cassette labeling of gallium-68 tracers**. Automated purification of cyclotron-produced gallium-68 and subsequent labeling of gallium-68 tracers. The green line represents the delivery line from the cyclotron target to the FASTLab, orange lines represent gallium-68 purification and formulation into [^68^Ga]GaCl_3_, purple lines represent the addition of [^68^Ga]GaCl_3_ into formulated reaction vessel and labeling reagents, blue lines represent the dose transfer line into the final dose vial. Black lines are vacuum lines, and grey lines are nitrogen overpressure lines. CG—chromatography; HF—high flow nitrogen (N_2_) gas; LF—low flow nitrogen (N_2_) gas; SAX A8—strong anion exchange A8 resin; SWFI—sterile water for injection; TK200—trioctyl phosphine 200 resin; ZR—hydroxamate resin. GE gallium chloride cassettes were modified as reported in “Improved purification of cyclotron [^68^Ga]GaCl_3_ for the production of ^68^Ga radiopharmaceuticals.” Subsequently, a formulated GE COC reactor was added with tubing connected to positions V4, V9, and V25. V9 was connected to the center luer lock of the reactor for the transferring of liquids. The pH adjustment (buffer) solution was added into an 11 mm crimped vial and placed in V2. A sterile 0.2 μm Millex-FG filter was attached to tubing in V5 to act as a secondary vent. Another tubing was added in V10 for the transferring of the product into the dose vial.

For the purification and formulation of [^68^Ga]Ga(NO_3_)_3_ into [^68^Ga]GaCl_3_, the (right-hand side, Figure 2) cassette layout is unchanged from the previously established method [3]. The on-cassette labeling layout modifies the left-hand side of the cassette to allow the addition of a buffer vial, tubing for the labeling reaction to occur in the formulated GE COC reactor, and a final transfer line for the labeled product into the dose vial (Figure 2). 

### 3.5. Radiolabeling, Formulation Compatibility, and Filter Efficiency

Production of [^68^Ga]GaCl_3_ using the FASTLab 2 module was modified to elute TK200 using 2.5 mL of ~0.1 M HCl. A previously developed metal readout assay was used to determine the suitability of the reduced-volume [^68^Ga]GaCl_3_ and determine its relative HCl molarity by labeling DOTA-TOC and checking the radiochemical purity (RCP) and pH [3]. In brief, the reduced-volume [^68^Ga]GaCl_3_ was added into an in-house formulated DOTA-TOC vial followed by 0.3 mL buffer and the reaction was heated at 100 °C for 8 min. An aliquot was obtained for RCP and pH determination. 

Automated labeling of the precursor was accomplished using a FASTLab 2 synthesizer. In the modified program, 2.5 mL (± module syringe driver tolerance) of [^68^Ga]GaCl_3_ in 0.1 M HCl was eluted from the TK200 resin into the formulated GE COC reactor containing a precursor (DOTA-TOC, FAPI-04, pentixafor, and PSMA-11). Immediately following elution, 0.5 mL of buffer solution was added, and the reactor was heated at either 60 °C for 5 min, or 100 °C for 8 min (Table 2). For reactions that were heated at 100 °C, the solutions were cooled for 4 min prior to transferring. For reactions that were heated at 60 °C, the solutions were cooled for 30 s prior to transferring. All products were transferred through a sterile filter (either Millex-LG, Millex-GP, Millex-GS, Millex-GV, or Cathivex-GV) into the product vial or manually filtered into the product vial to determine the amount of activity retained on the filter. An aliquot was obtained to determine RCP by radioTLC and radioHPLC. For validation runs, an aliquot of the terminally filtered product was tested for quality control. 

### 3.6. Pre-Release Quality Control and Analysis 

See Supplementary Materials for copies of radioTLC and radioHPLC traces.

#### 3.6.1. Radiochemical Purity (RCP) and Stability by RadioTLC (rTLC) and RadioHPLC (rHPLC)

The aliquots obtained during workflow development and validation runs were analyzed for radiochemical purity (RCP) by both rTLC and rHPLC, with the passing RCP being > 95%. For rTLC and rHPLC, separate aliquots were obtained from the final vial at the initial timepoint and then repeated at either 1 h or 4 h to determine the stability of the product after labeling. rTLC: Glass fiber rTLC plates impregnated with silica acid were used as the solid phase. For DOTA-TOC, pentixafor, and PSMA-11, the plates were developed in a 1 M ammonia acetate in methanol solution (1:1) to analyze RCP. For FAPI-04, a 5 M ammonia acetate in methanol solution (1:1) was used to analyze RCP. All samples were analyzed using a Bioscan AR2000 rTLC plate scanner. [^68^Ga]Ga-DOTA-TOC had an Rf between 0.6 and 0.8, [^68^Ga]Ga-DOTA-FAPI-04 Rf between 0.4 and 1.0, [^68^Ga]Ga-DOTA-pentixafor Rf between 0.5 and 1.0, and [^68^Ga]Ga-HBED-CC-PSMA-11 Rf between 0.6 and 1.0, with free gallium-68 or gallium-68 colloids at the origin (with Rf ~ 0). 

rHPLC: Using a Luna Omega 5 μm Polar C18 100 Å, LC column 150 × 4.6 mm (pn: 00F-4754-E0) at 40 °C with a CBM-20A liquid chromatograph (Shimadzu) connected to a Bioscan detector, UV at 254 nm, radioactive decay (in CPM) was monitored. The following solvent systems were used: A: H_2_O and 0.1% TFA; B: MeCN and 0.1% TFA. Either (1) a step gradient of 0% B 5 min, 70% B 1 min, then 0% B 4 min or (2) 0–70% B 6 min, then 0% B 4 min was used, both with a flow rate of 2 mL/min. The rHPLC method was adapted from previous quality control measures for PSMA-11 [2].

#### 3.6.2. Suitability by pH and Visual Inspection 

Samples were visually inspected for clarity, color, and the presence of particulates at the initial timepoint and at 4 h for stability. Using pH indicator strips (MQuant), the pH of the samples was determined. All labeled products should be clear, colorless to slightly yellow, and free from particulates, with a pH of 3.0 to 4.5. 

#### 3.6.3. Sterile Filter Integrity Test and Bacterial Endotoxins

As part of the validation process, a product sterility test was completed according to current good manufacturing practices (cGMP) and our facility’s quality assurance (QA) program. A filter integrity test was completed by pushing air through a 0.22 μm filter used during terminal filtration. The filter should withstand at minimum 50 psi of pressure. Bacterial endotoxin analysis was performed using the Limulus amebocyte lysate method with an Endosafe-PTS test system (Charles River Laboratories International) using a 1:300 dilution. All labeled products should have an endotoxin unit of ≤53.8 EU/mL. 

### 3.7. Post-Release/Periodic Quality Control and Analysis 

#### 3.7.1. Sterility Testing Using Tryptic Soy Broth (TSB) and Fluid Thioglycolate Medium (FTM)

As part of the validation process, passing doses to be released for use were tested for bacterial contamination using tryptic soy broth (TSB) and a fluid thioglycolate medium (FTM). These samples were monitored on days 5, 7, and 14 according to USP <71> [21]. A total of 250 μL of the sample was added into the TSB and FTM media and inverted once. The TSB was incubated at room temperature with deviations from 20 to 25 °C. The FTM was incubated at 32 ± 0.5 °C. Samples were passing if they were clear with no turbidity after 14 days of monitoring. 

#### 3.7.2. Radionuclidic Purity (RNP) by γ-Spectrometry

Radionuclidic purity was tested as part of validation runs, and periodically thereafter as a periodic quality indicator test (PQIT). We tested this weekly to confirm the suitability of the gallium-68 produced using the cyclotron [2]. Radionuclidic purity is determined by γ-spectrometry using a multi-channel analyzer (MCA, Canberra) with an energy window of 8–2048 keV. Samples (25 μL) were counted 20–24 h after the end of synthesis (EOS) for a minimum of 25 min to quantify radionuclidic impurities such as ^66^Ga (t_1/2_ = 9.49 h) and ^67^Ga (t_1/2_ = 78.3 h), where passing RNP was >98% (or ^66^Ga/^67^Ga ≤ 2%). 

### 3.8. Statistics

All statistics are represented as mean ± standard deviation unless otherwise specified. 

## 4. Conclusions

The validation of clinically relevant tracers (DOTA-TOC, FAPI-04, pentixafor, and PSMA-11) with DOTA and HBED-CC chelators using cyclotron-produced [^68^Ga]GaCl_3_ with our on-cassette method is demonstrated to be robust and suitable for the manufacturing of research tracers for initial IND and RDRC studies. For tracers that do not require post-labeling purification, a similar workflow, cassette layout, and timelist allow the screening of numerous novel and diverse research tracers for potential use with gallium-68 as a diagnostic agent. The on-cassette method allows the use of a non-ISO-rated clean room to prepare precursor reactors, decreasing costs and increasing the feasibility of clinical translation for promising research tracers for new indications and studies. This automated procedure can also be adapted to other SPECT, PET, and beta or alpha radiometals for novel diagnostic or therapeutic purposes. 

## Figures and Tables

**Table 1:** Tracers of interest, their properties, and indications.

Peptide	Chelator	Properties	Receptor	Indications
NOC	DOTA	Cyclic, disulfide peptide	SSTR	Neuroendocrine neoplasms (NENs) and neuroendocrine tumors (NETs)
TOC				
TATE				
FAPI-04	DOTA	Quinoline-based, proline-glycine, peptidomimetic	FAP	Numerous generalized cancers
Pentixafor	DOTA	Cyclic, non-disulfide peptide	CXCR4	Lymphoma, NETs, adrenal adenomas, in hyperaldosteronism and hypercortisolism [12]
PSMA-11	HBED-CC	Linear, 2 amino acid, primary structure, peptidomimetic	PSMA	Prostate cancer

**Table 2:** Percent activity retained using DOTA-TOC on different filter membrane materials, using manual filtration method for terminal sterilization of dose.

	Millex-LG	Millex-GP	Millex-GS	Millex-GV	Cathivex-GV
Filter Identifier Color	Grey	Green	Blue	Gold	Gold/White
Membrane Material	PTFE	PES	MCE	PVDF	PVDF
Vented Filter	No	No	No	No	Yes
Filter Size (μm)	0.2	0.22	0.22	0.22	0.22
% DOTA-TOC Activity Trapped	17.41	23.72	99.67	12.38	6.54

PTFE—polytetrafluoroethylene, PES—hydrophilic polyethersulfone, MCE—mixed cellulose esters, PVDF—hydrophilic polyvinylidenefluroide, ND—no data. All data n = 1.

**Table 3:** Formulation of precursors, their quantities, reaction temperature, heating duration, and active cooling duration.

Precursor	Precursor (μg)	Precursor (nmol)	Stock Solution Solvent	Formulation	Rxn Temp. (°C)	Heating Time (min)	Active Cooling Time (min)
DOTA-TOC	40	28	MQ water	NetSpot	100	8	4
FAPI-04	40	33	0.5% MeCN	NetSpot	100	8	4
Pentixafor	25	29	MQ water	NetSpot	100	8	4
PSMA-11	30	32	MQ water	NetSpot	60	5	0.5

**Table 4:** Radiochemical purity for validating FAPI-04 and pentixafor and for “pseudo-validation” of PSMA-11.

Average Values	FAPI-04	Pentixafor	“PSMA-11”
Sample size (n)	3	3	1 + 2 *
Calibrated activity (MBq) **	1273 ± 60.7	1288 ± 64.4	1177 ± 31.8
Radiochemical yield (%)	20.9 ± 1.00	21.1 ± 1.06	19.2 ± 0.52
Molar activity (Gbq/μmol)	38.8 ± 1.85	45.0 ± 2.25	37.2 ± 1.00
Volume of dose (mL)	2.7 ± 0.00	2.7 ± 0.08	2.5 ± 0.00
RCP (rTLC, %)	98.77 ± 0.12	98.64 ± 0.26	98.83 ± 0.19
RCP (rHPLC, %)	98.4 ± 0.17	99.67 ± 0.00	99.82 ± 0.00
4 h RCP (rTLC, %)	98.17 ± 0.53	98.43 ± 0.19	98.84 ± 0.10
4 h RCP (rHPLC, %)	98.26 ± 0.05	99.15 ± 0.12	99.84 ± 0.00

* Two of the PSMA-11 runs were “pseudo-validations” in which some of the quality control (filter integrity, endotoxin, sterility) tests were not performed. ** Calculated starting activity is 6.1 GBq of [^68^Ga]Ga(NO_3_)_3_ prior to its purification and formulation into [^68^Ga]GaCl_3_ for addition into the reactor. Data are reported as mean ± standard deviation.

## Supplementary Materials

The following supporting information can be downloaded at: https://www.mdpi.com/article/10.3390/molecules29225457/s1, Figure S1: radioTLC of [^68^Ga]Ga-DOTATOC using reduced volume (2.5 mL) TK200 elution; Figure S2: radioTLC of filtered [^68^Ga]Ga-DOTATOC using 0.22 μm Millex-GV Filter; Figure S3: radioTLC of filtered [^68^Ga]Ga-DOTATOC using 0.2 μm Millex-LG Filter; Figure S4: radioTLC of filtered [^68^Ga]Ga-DOTATOC using 0.22 μm Millex-GP Filter; Figure S5: radioTLC of filtered [^68^Ga]Ga-DOTATOC using 0.22 μm Millex-GS Filter; Figure S6: radioTLC of filtered [^68^Ga]Ga-DOTATOC using 0.22 μm Cathivex-GV Filter following 48 mL air to dry filter; Figure S7: radioTLC of FAPI-04 Validation #1 at initial timepoint (5M NH4OAc: MeOH (1:1)); Figure S8: radioTLC of FAPI-04 Validation #2 at initial timepoint (5M NH4OAc: MeOH (1:1)); Figure S9: radioTLC of FAPI-04 Validation #3 at initial timepoint (5M NH4OAc: MeOH (1:1)); Figure S10: radioHPLC of FAPI-04 Validation #1 at initial timepoint; Figure S11: radioHPLC of FAPI-04 Validation #2 at initial timepoint; Figure S12: radioHPLC of FAPI-04 Validation #3 at initial timepoint; Figure S13: radioTLC of FAPI-04 Validation #1 at 4-h timepoint (5M NH4OAc: MeOH (1:1)); Figure S14: radioTLC of FAPI-04 Validation #2 at 4-h timepoint (5M NH4OAc: MeOH (1:1)); Figure S15: radioTLC of FAPI-04 Validation #3 at 4-h timepoint (5M NH4OAc: MeOH (1:1)); Figure S16: radioHPLC of FAPI-04 Validation #1 at 4-h timepoint; Figure S17: radioHPLC of FAPI-04 Validation #2 at 4-h timepoint; Figure S18: radioHPLC of FAPI-04 Validation #3 at 4-h timepoint; Figure S19: radioTLC of Pentixafor Validation #1 at initial timepoint; Figure S20: radioTLC of Pentixafor Validation #2 at initial timepoint; Figure S21: radioTLC of Pentixafor Validation #3 at initial timepoint; Figure S22: radioHPLC of Pentixafor Validation #1 at initial timepoint; Figure S23: radioHPLC of Pentixafor Validation #2 at initial timepoint; Figure S24: radioHPLC of Pentixafor Validation #3 at initial timepoint; Figure S25: radioTLC of Pentixafor Validation #1 at 4-h timepoint; Figure S26: radioTLC of Pentixafor Validation #2 at 4-h timepoint; Figure S27: radioTLC of Pentixafor Validation #3 at 4-h timepoint; Figure S28: radioHPLC of Pentixafor Validation #1 at 4-h timepoint; Figure S29: radioHPLC of Pentixafor Validation #2 at 4-h timepoint; Figure S30: radioHPLC of Pentixafor Validation #3 at 4-h timepoint; Figure S31: radioTLC of PSMA-11 Validation #1 at initial timepoint; Figure S32: radioTLC of PSMA-11 pseudo-validation #1 at initial imepoint; Figure S33: radioTLC of PSMA-11 pseudo-validation #2 at initial timepoint; Figure S34: radioHPLC of PSMA-11 Validation #1 at initial timepoint; Figure S35: radioHPLC of PSMA-11 pseudo-validation #1 at initial timepoint; Figure S36: radioHPLC of PSMA-11 pseudo-validation #2 at initial timepoint; Figure S37: radioTLC of PSMA-11 Validation #1 at 4-h timepoint; Figure S38: radioTLC of PSMA-11 pseudo-validation #1 at 4-h timepoint; Figure S39: radioTLC of PSMA-11 pseudo-validation #2 at 4-h timepoint; Figure S40: radioHPLC of PSMA-11 Validation #1 at 4-h timepoint; Figure S41: radioHPLC of PSMA-11 pseudo-validation #1 at 4-h timepoint; Figure S42: radioHPLC of PSMA-11 pseudo-validation #2 at 4-h timepoint. Table S1: Components in formulated GE COC reactors following after NetSpot (Dotatate) formulation; Table S2: Summary of Validation for FAPI-04; Table S3: Summary of Validation for Pentixafor; Table S4: Summary of Validation for PSMA-11.
