# Supplementary material for: Towards a General Method for Using Cyclotron-Produced Ga68 to Manufacture Clinical and Research Ga68 Tracers
Source: Molecules. 2024 Nov 19;29(22):5457. doi: 10.3390/molecules29225457 (PMC11597648; doi:10.3390/molecules29225457)
Supplement: Supplementary file 1 [file molecules-29-05457-s001.zip › molecules-3271471-supplementary.pdf]

A General Method for Using Cyclotron Produced Ga68 to Manufacture Clinical and Research Ga68 Tracers

Ivan E. Wang[a], Kevin Cheng, Allen F. Brooks[a], Peter J. H. Scott[a], Benjamin L. Viglianti[a]

[a]Division of Nuclear Medicine, Department of Radiology, University of Michigan, 1301 Catherine St. 2276 Medical Science I, Ann Arbor MI, 48109, United States of America

Table S1: Components in formulated GE COC reactors following after NetSpot (Dotatate) formulation.
Table S2: Summary of Validation for FAPI-04
Table S3: Summary of Validation for Pentixafor
Table S4: Summary of Validation for PSMA-11

Figure S1: radioTLC of [^{68}Ga]Ga-DOTATOC using reduced volume (2.5 mL) TK200 elution
Figure S2: radioTLC of filtered [^{68}Ga]Ga-DOTATOC using 0.22 μm Millex-GV Filter
Figure S3: radioTLC of filtered [^{68}Ga]Ga-DOTATOC using 0.2 μm Millex-LG Filter
Figure S4: radioTLC of filtered [^{68}Ga]Ga-DOTATOC using 0.22 μm Millex-GP Filter
Figure S5: radioTLC of filtered [^{68}Ga]Ga-DOTATOC using 0.22 μm Millex-GS Filter
Figure S6: radioTLC of filtered [^{68}Ga]Ga-DOTATOC using 0.22 μm Cathivex-GV Filter following 48 mL air to dry filter
Figure S7: radioTLC of FAPI-04 Validation #1 at initial timepoint (5M NH_4OAc : MeOH (1:1))
Figure S8: radioTLC of FAPI-04 Validation #2 at initial timepoint (5M NH_4OAc : MeOH (1:1))
Figure S9: radioTLC of FAPI-04 Validation #3 at initial timepoint (5M NH_4OAc : MeOH (1:1))
Figure S10: radioHPLC of FAPI-04 Validation #1 at initial timepoint
Figure S11: radioHPLC of FAPI-04 Validation #2 at initial timepoint
Figure S12: radioHPLC of FAPI-04 Validation #3 at initial timepoint
Figure S13: radioTLC of FAPI-04 Validation #1 at 4-hr timepoint (5M NH_4OAc : MeOH (1:1))
Figure S14: radioTLC of FAPI-04 Validation #2 at 4-hr timepoint (5M NH_4OAc : MeOH (1:1))
Figure S15: radioTLC of FAPI-04 Validation #3 at 4-hr timepoint (5M NH_4OAc : MeOH (1:1))
Figure S16: radioHPLC of FAPI-04 Validation #1 at 4-hr timepoint
Figure S17: radioHPLC of FAPI-04 Validation #2 at 4-hr timepoint
Figure S18: radioHPLC of FAPI-04 Validation #3 at 4-hr timepoint
Figure S19: radioTLC of Pentixafor Validation #1 at initial timepoint
Figure S20: radioTLC of Pentixafor Validation #2 at initial timepoint
Figure S21: radioTLC of Pentixafor Validation #3 at initial timepoint
Figure S22: radioHPLC of Pentixafor Validation #1 at initial timepoint
Figure S23: radioHPLC of Pentixafor Validation #2 at initial timepoint
Figure S24: radioHPLC of Pentixafor Validation #3 at initial timepoint
Figure S25: radioTLC of Pentixafor Validation #1 at 4-hr timepoint
Figure S26: radioTLC of Pentixafor Validation #2 at 4-hr timepoint
Figure S27: radioTLC of Pentixafor Validation #3 at 4-hr timepoint
Figure S28: radioHPLC of Pentixafor Validation #1 at 4-hr timepoint
Figure S29: radioHPLC of Pentixafor Validation #2 at 4-hr timepoint
Figure S30: radioHPLC of Pentixafor Validation #3 at 4-hr timepoint
Figure S31: radioTLC of PSMA-11 Validation #1 at initial timepoint
Figure S32: radioTLC of PSMA-11 pseudo-validation #1 at initial timepoint
Figure S33: radioTLC of PSMA-11 pseudo-validation #2 at initial timepoint
Figure S34: radioHPLC of PSMA-11 Validation #1 at initial timepoint
Figure S35: radioHPLC of PSMA-11 pseudo-validation #1 at initial timepoint
Figure S36: radioHPLC of PSMA-11 pseudo-validation #2 at initial timepoint
Figure S37: radioTLC of PSMA-11 Validation #1 at 4-hr timepoint
Figure S38: radioTLC of PSMA-11 pseudo-validation #1 at 4-hr timepoint

Figure S39: radioTLC of PSMA-11 pseudo-validation #2 at 4-hr timepoint
Figure S40: radioHPLC of PSMA-11 Validation #1 at 4-hr timepoint
Figure S41: radioHPLC of PSMA-11 pseudo-validation #1 at 4-hr timepoint
Figure S42: radioHPLC of PSMA-11 pseudo-validation #2 at 4-hr timepoint

Table S1: Components in formulated GE COC reactors following after NetSpot (Dotatate) formulation.

	Formulated Reactor				
	Component	Use	mass (µg)	mass (µmol)	volume (mL)
Ligand Vial	Precursor	Ligand	25-52	0.028-0.033	-
	1,10-phenanthroline	Chelator	5	0.02775	-
	Mannitol	Bulking agent	20000	109.79	-
	Dihydroxybenzoic acid (gentisic acid)	Radioprotectant	6	0.03893	-
Buffer Vial	Formic Acid	Buffer (Acid)	30000	651.82	-
	Sodium Hydroxide	Buffer (Base)	28250	706.30	-
	SWFI	Diluent	-	-	qs 0.5

Table S2: Summary of Validation for FAPI-04

	Criteria	FAPI4-V1 (IEW-07-04)	FAPI4-V2 (IEW-07-05)	FAPI4-V3 (IEW-07-06)	Average (n=3)	Passing Criteria	Units
Cyclotron and Purification	Cyclotron Beam Current	50	50	50	50	-	µA
	Cyclotron Beam Time	60.5	61	61	60.83	-	min
	Step222 Syringe Displacement	20.4	20.6	20.4	20.5	-	mm
	Equivalent volume Loaded onto SAX+TK200	2.4684	2.4926	2.4684	2.476	-	mL
	Length of Purification	25.65	26.65	26.95	26.42	-	min
	Length of Labeling	23.00	23.00	22.00	22.67		min
Activities and Assay	Activity of FAPI4 at Cal	36.5	34.1	32.5	34.37	-	mCi
	Decayed to EOB	62.01	59.13	56.36	59.17	-	mCi
	Volume of FAPI4	2.7	2.7	2.7	2.70	-	mL
	Assay at Cal	13.52	12.63	12.04	12.73	-	mCi/mL
	Assay at EOB	22.97	21.90	20.87	21.91	-	mCi/mL
Quality Control and Sterility	RCP (rTLC)	98.61	98.89	98.82	98.77	≥ 90	%
	Impurities (rTLC)	1.02	0.81	0.77	0.87	≤ 10	%
	Radiochemical Identity (Rf)	0.546	0.588	0.608	0.58	0.4-1.0	-
	RCP (HPLC)	98.568	98.238	98.400	98.40	≥ 90	%
	Impurities (HPLC)	1.432	1.762	1.600	1.60	≤ 10	%
	Radiochemical Identity (Rt)	4.508	4.533	4.542	4.53	4-5	min
	pH	3.0-3.3	3.0-3.3	3.0-3.3	3.15	3.0 - 4.5	-
	Appearance and Visual Inspection	clear, colorless, no ppt	clear, colorless, no ppt	clear, colorless, no ppt	-	clear, colorless to slight yellow, no ppt	-
	Filter Bubble Point Test	62	62	60	61.33	≥ 50	psi
	Bacterial Endotoxin	< 15.0	< 15.0	< 15.0	-	≤ 58.3	EU/mL
	Radionuclidic Inpurities (66Ga/67Ga)	0.15	0.173	0.183	0.169	≤ 2	%
	Radionuclidic Purity (RNP)	99.85	99.827	99.817	99.831	> 98	%
	Bacterial contamination	Clear, not turbid	Clear, not turbid	Clear, not turbid	-	Clear, not turbid	-
Radiochemical Stability at Expiration (4 hours)	RCP (rTLC)	97.46	98.74	98.32	98.17	≥ 90	%
	Impurities (rTLC)	1.06	0.88	1.28	1.07	≤ 10	%
	Radiochemical Identity (Rf)	0.637	0.563	0.551	0.58	0.4-1.0	-
	RCP* (HPLC)	98.307	98.211	98.255	98.26	≥ 90	%
	Impurities* (HPLC)	1.694	1.79	1.745	1.74	≤ 10	%
	Radiochemical Identity (Rt)	4.542	4.533	4.533	4.54	4-5	min
	Appearance and Visual Inspection	clear, colorless, no ppt	clear, colorless, no ppt	clear, colorless, no ppt	-	clear, colorless to slight yellow, no ppt	-

*For 4-hour stability, a 20 µL injection was used instead of 10 µL since the impurity peak becomes indistinguishable from background noise.

Table S3: Summary of Validation for Pentixafor

	Criteria	PTXA4-V1 (IEW-07-07)	PTXA4-V2 (IEW-07-08)	PTXA4-V3 (IEW-07-09)	Average (n=3)	Passing Criteria	Units
Cyclotron and Purification	Cyclotron Beam Current	50	50	50	50	-	µA
	Cyclotron Beam Time	61	61	61	61.00	-	min
	Step222 Syringe Displacement	20.4	20.4	20.4	20.4	-	mm
	Equivalent volume Loaded onto SAX+TK200	2.4684	2.4684	2.4684	2.468	-	mL
	Length of Purification	26.08	26.83	26.63	26.52	-	min
	Length of Labeling	22.00	23.00	22.00	22.33		min
Activities and Assay	Activity of PTXA4 at Cal	37.2	33.2	33.9	34.77	-	mCi
	Decayed to EOB	65.17	56.99	59.39	60.51	-	mCi
	Volume of PTXA4	2.8	2.6	2.7	2.70	-	mL
	Assay at Cal	13.29	12.77	12.56	12.87	-	mCi/mL
	Assay at EOB	23.27	21.92	21.99	22.40	-	mCi/mL
Quality Control and Sterility	RCP (rTLC)	98.27	98.85	98.8	98.64	≥ 90	%
	Impurities (rTLC)	1.34	0.81	0.77	0.97	≤ 10	%
	Radiochemical Identity (Rf)	0.669	0.663	0.749	0.69	0.5-1.0	-
	RCP (HPLC)	99.674	99.676	99.648	99.67	≥ 90	%
	Impurities (HPLC)	0.326	0.324	0.352	0.33	≤ 10	%
	Radiochemical Identity (Rt)	5	5.017	5	5.01	4-5	min
	pH	3.6	3.6	3.6	3.15	3.0 - 4.5	-
	Appearance and Visual Inspection	clear, colorless, no ppt	clear, colorless, no ppt	clear, colorless, no ppt	-	clear, colorless to slight yellow, no ppt	-
	Filter Bubble Point Test	62	61	65	62.67	≥ 50	psi
	Bacterial Endotoxin	< 15.0	< 15.0	< 15.0	-	≤ 58.3	EU/mL
	Radionuclidic Inpurities (66Ga/67Ga)	0.153	0.153	0.173	0.160	≤ 2	%
	Radionuclidic Purity (RNP)	99.847	99.847	99.827	99.840	> 98	%
	Bacterial contamination	Clear, not turbid	Clear, not turbid	Clear, not turbid	-	Clear, not turbid	-
Radiochemical Stability at Expiration (4 hours)	RCP (rTLC)	98.31	98.7	98.29	98.43	≥ 90	%
	Impurities (rTLC)	1.39	1.03	1.39	1.27	≤ 10	%
	Radiochemical Identity (Rf)	0.649	0.617	0.776	0.68	0.5-1.0	-
	RCP* (HPLC)	99.203	98.959	99.282	99.15	≥ 90	%
	Impurities* (HPLC)	0.798	1.041	0.718	0.85	≤ 10	%
	Radiochemical Identity (Rt)	5.008	5.017	5.008	5.01	4-5	min
	Appearance and Visual Inspection	clear, colorless, no ppt	clear, colorless, no ppt	clear, colorless, no ppt	-	clear, colorless to slight yellow, no ppt	-

*For 4-hour stability, a 20 µL injection was used instead of 10 µL since the impurity peak becomes indistinguishable from background noise.

Table S4: Summary of Validation for PSMA-11

	Criteria	PSMA11-V1 (IEW-07-11)	PSMA11-Chem1** (IEW-07-12)	PSMA11-Chem2**,*** (IEW-07-13)	Average (n=3)	Passing Criteria	Units
Cyclotron and Purification	Cyclotron Beam Current	50	50	49.79	49.93	-	µA
	Cyclotron Beam Time	61	61	56	59.33	-	min
	Step222 Syringe Displacement	20.4	20.4	20.4	20.4	-	mm
	Equivalent volume Loaded onto SAX+TK200	2.4684	2.4684	2.4684	2.468	-	mL
	Length of Purification	22.42	23.38	22.90	22.90	-	min
	Length of Labeling	16.00	15.00	16.00	15.67		min
Activities and Assay	Activity of PSMA11 at Cal	31.2	31.4	32.8	31.80	-	mCi
	Decayed to EOB	48.86	49.17	52.42	50.15	-	mCi
	Volume of PSMA11	2.5	2.5	2.5	2.50	-	mL
	Assay at Cal	12.48	12.56	13.12	12.72	-	mCi/mL
	Assay at EOB	19.54	19.67	20.97	20.06	-	mCi/mL
Quality Control and Sterility	RCP (rTLC)	98.94	98.57	98.98	98.83	≥ 95	%
	Impurities (rTLC)	0.69	1.09	0.6	0.79	≤ 5	%
	Radiochemical Identity (Rf)	0.777	0.756	0.847	0.79	0.6-1.0	-
	RCP (HPLC)	> 99.9	99.459	> 99.9	99.82	≥ 95	%
	Impurities (HPLC)	< 0.01	0.541	< 0.01	0.54	≤ 5	%
	Radiochemical Identity (Rt)	4.65	4.617	4.608	4.63	4-5	min
	pH	3.3-3.6	3.0-3.3	3.0-3.3	3.15	3.0 - 4.5	-
	Appearance and Visual Inspection	clear, colorless, no ppt	clear, colorless, no ppt	clear, colorless, no ppt	-	clear, colorless to slight yellow, no ppt	-
	Filter Bubble Point Test	60	*Not completed, n/a*	*Not completed, n/a*	-	≥ 50	psi
	Bacterial Endotoxin	< 15.0	*Not completed, n/a*	*Not completed, n/a*	-	≤ 58.3	EU/mL
	Radionuclidic Inpurities (66Ga/67Ga)	0.116	0.136	0.127	0.126	≤ 2	%
	Radionuclidic Purity (RNP)	99.884	99.864	99.873	99.874	> 98	%
	Bacterial contamination	Clear, not turbid	*Not completed, n/a*	*Not completed, n/a*	-	Clear, not turbid	-
Radiochemical Stability at Expiration (4 hours)	RCP (rTLC)	98.7	98.88	98.93	98.84	≥ 95	%
	Impurities (rTLC)	0.97	0.91	0.77	0.88	≤ 5	%
	Radiochemical Identity (Rf)	0.75	0.733	0.764	0.75	0.6-1.0	-
	RCP* (HPLC)	> 99.9	99.525	> 99.9	99.84	≥ 95	%
	Impurities* (HPLC)	< 0.01	0.475	< 0.01	0.48	≤ 5	%
	Radiochemical Identity (Rt)	4.65	4.633	4.633	4.64	4-5	min
	Appearance and Visual Inspection	clear, colorless, no ppt	clear, colorless, no ppt	clear, colorless, no ppt	-	clear, colorless to slight yellow, no ppt	-

*For 4-hour stability, a 30 µL injection was used instead of 10 µL since the impurity peak becomes indistinguishable from background noise.

**Not a validation run, only testing chemistry therefore dose was not sterile, not assembled in LFH (filters were still used), no filter bubble point test, endotoxin, or sterility completed

***During intial irradiation, there was a overpressure issue at 26 minutes which interrupted the irradiation for 10 minutes (decay). A second irradiation was started at 50 µA. After 26 minutes the current was decreased to 49 µA then irradiated for another 14 minutes. The cyclotron beam current was caluclate using the weighted mean current and the cyclotron bean time was the total irradiation time

Figure S1: radioTLC of [^{68}Ga]Ga-DOTATOC using reduced volume (2.5 mL) TK200 elution

Method: TATE File: 060223TOC01.R001

Instrument Parameters

Method:	TATE	File:	060223TOC01.R001
Evaluated:	02 Jun 2023 16:25:19	Created:	02 Jun 2023 16:25:19
Evaluation by:	LM		
Collimator Type:	Hi Efficiency	Width:	10 mm
Elect. Resol:	Normal	Amp. Range:	50 - 2047
Resolution:	256 chan	Chan Size:	0.864 mm
Hi Voltage:	1526 Volts	Chan of Zero mm:	10.3
Run Time:	1.00 min	Max Count:	999999
Relative Pos:	0.0 mm		

Comments

Default Method for use in defining other methods

Analysis Parameters

Bkg Subtraction:	none	Origin:	60.0 mm
Normalization:	none	Front:	115.0 mm
Total Counts:	66672.0 (66672.0 CPM)	Region:	50.0 - 140.0 mm
Total File Counts:	66672		

Region Analysis

Definition: Table

Reg	(mm) Start	(mm) Stop	(mm) Centroid	RF	Region Counts	Region CPM	% of Total	% of ROI
Rgn 1	49.0	73.1	60.4	0.007	1276.0	1276.0	1.91	1.92
Rgn 2	73.1	141.4	100.0	0.728	65211.0	65211.0	97.81	98.08
2 Peaks					66487.0	66487.0	99.72	100.00

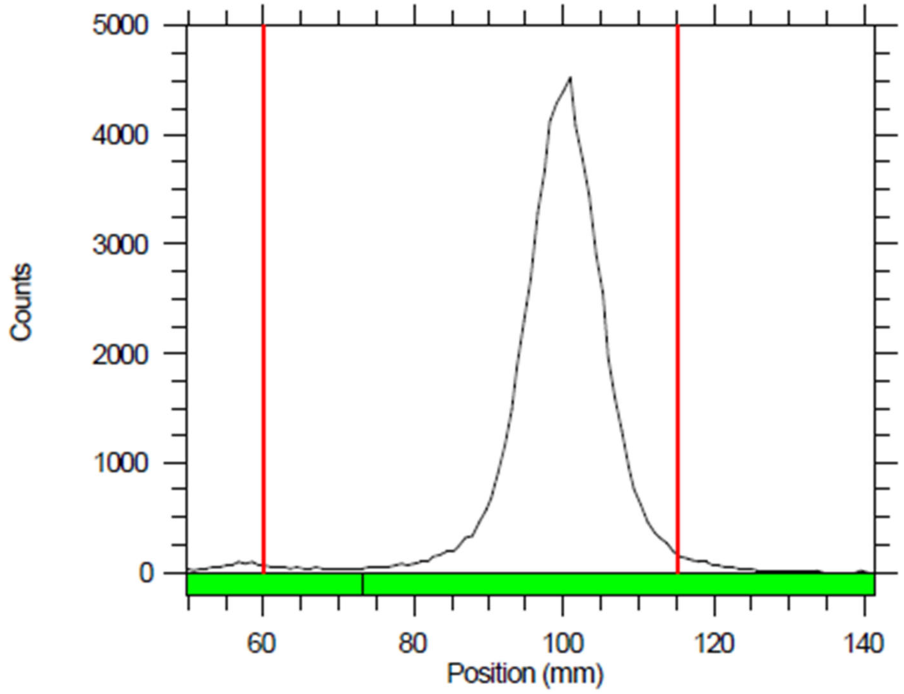

Figure S2: radioTLC of filtered [^{68}Ga]Ga-DOTATOC using 0.22 µm Millex-GV Filter

Method: TATE File: 072723TOC_V.R001

Instrument Parameters

Method:	TATE	File:	072723TOC_V.R001
Evaluated:	27 Jul 2023 10:51:07	Created:	27 Jul 2023 10:51:07
Evaluation by:	IEW		
Collimator Type:	Hi Efficiency	Width:	10 mm
Elect. Resol:	Normal	Amp. Range:	50 - 2047
Resolution:	256 chan	Chan Size:	0.864 mm
Hi Voltage:	1528 Volts	Chan of Zero mm:	10.3
Run Time:	1.00 min	Max Count	999999
Relative Pos:	0.0 mm		

Comments

Default Method for use in defining other methods

Analysis Parameters

Bkg Subtraction:	none	Origin:	60.0 mm
Normalization:	none	Front:	115.0 mm
Total Counts:	81365.0 (81365.0 CPM)	Region:	50.0 - 140.0 mm
Total File Counts:	81365		

Region Analysis

Definition: Table

Reg	(mm) Start	(mm) Stop	(mm) Centroid	RF	Region Counts	Region CPM	% of Total	% of ROI
Rgn 1	38.6	69.7	59.6	-0.007	483.0	483.0	0.59	0.59
Rgn 2	69.7	140.5	99.1	0.711	80802.0	80802.0	99.31	99.41
2 Peaks					81285.0	81285.0	99.90	100.00

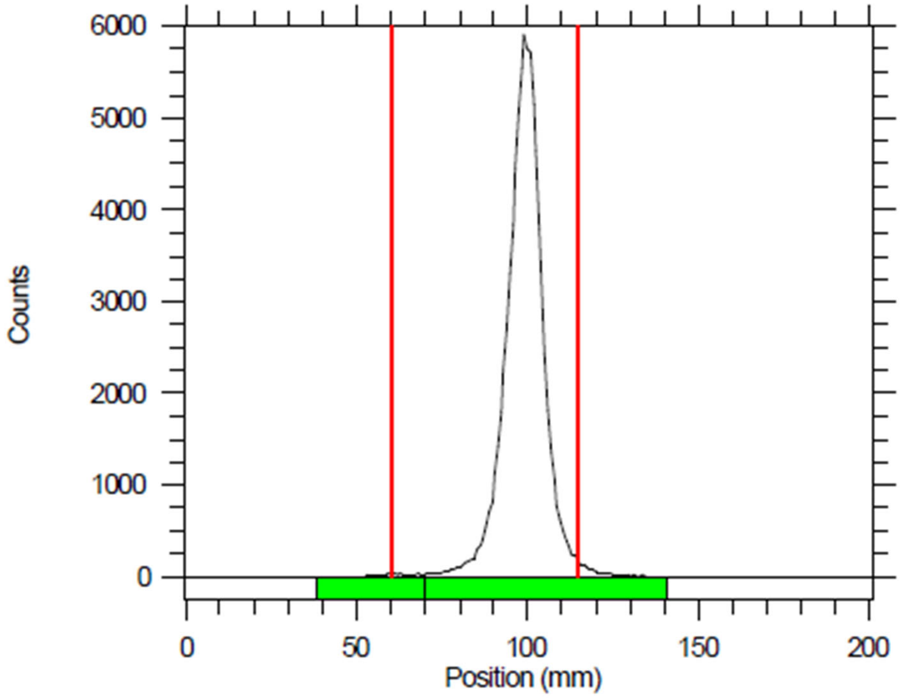

Figure S3: radioTLC of filtered [^{68}Ga]Ga-DOTATOC using 0.2 µm Millex-LG Filter

Method: TATE File: 080223-PTFE.R001A

Instrument Parameters

Method:	TATE	File:	080223-PTFE.R001A
Evaluated:	02 Aug 2023 11:17:19	Created:	02 Aug 2023 10:32:45
Evaluation by:	IEW		
Collimator Type:	Hi Efficiency	Width:	10 mm
Elect. Resol:	Normal	Amp. Range:	50 - 2047
Resolution:	256 chan	Chan Size:	0.864 mm
Hi Voltage:	1526 Volts	Chan of Zero mm:	10.3
Run Time:	1.00 min	Max Count	999999
Relative Pos:	0.0 mm		

Comments

Default Method for use in defining other methods

Analysis Parameters

Bkg Subtraction:	none	Origin:	60.0 mm
Normalization:	none	Front:	115.0 mm
Total Counts:	107634.0 (107634.0 CPM)	Region:	50.0 - 140.0 mm
Total File Counts:	107634		

Region Analysis

Definition: Table

Reg	(mm) Start	(mm) Stop	(mm) Centroid	RF	Region Counts	Region CPM	% of Total	% of ROI
Rgn 1	49.0	73.1	61.7	0.031	1050.0	1050.0	0.98	0.98
Rgn 2	72.3	128.4	100.2	0.731	106332.0	106332.0	98.79	99.02
2 Peaks					107382.0	107382.0	99.77	100.00

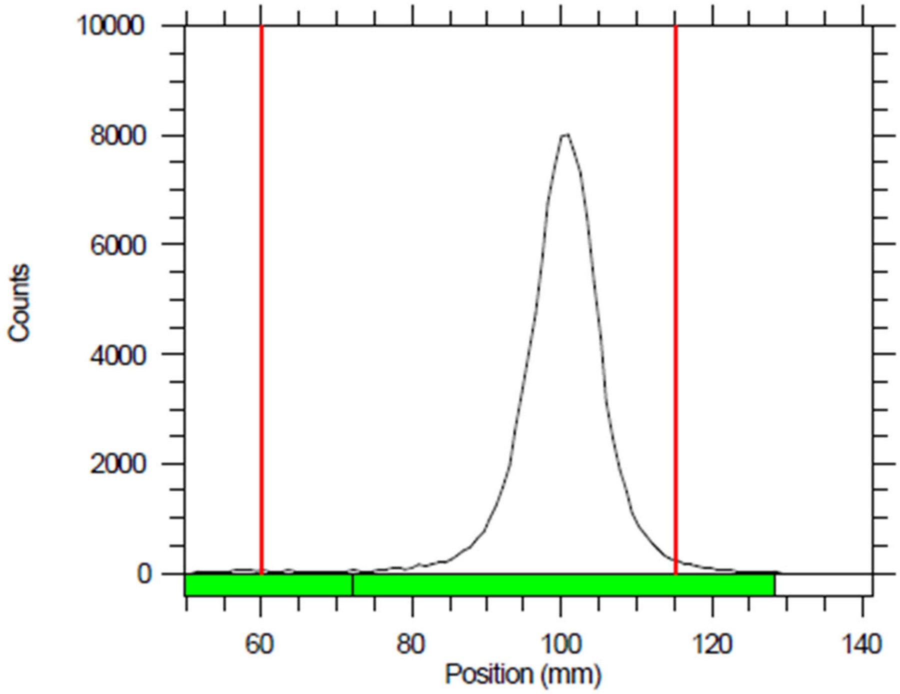

Figure S4: radioTLC of filtered [^{68}Ga]Ga-DOTATOC using 0.22 µm Millex-GP Filter

Method: TATE File: 080223-DOSEP.R001A

Instrument Parameters

Method:	TATE	File:	080223-DOSEP.R001A
Evaluated:	02 Aug 2023 11:17:00	Created:	02 Aug 2023 10:28:35
Evaluation by:	IEW		
Collimator Type:	Hi Efficiency	Width:	10 mm
Elect. Resol:	Normal	Amp. Range:	50 - 2047
Resolution:	256 chan	Chan Size:	0.864 mm
Hi Voltage:	1527 Volts	Chan of Zero mm:	10.3
Run Time:	1.00 min	Max Count	999999
Relative Pos:	0.0 mm		

Comments

Default Method for use in defining other methods

Analysis Parameters

Bkg Subtraction:	none	Origin:	60.0 mm
Normalization:	none	Front:	115.0 mm
Total Counts:	84520.0 (84520.0 CPM)	Region:	50.0 - 140.0 mm
Total File Counts:	84520		

Region Analysis

Definition: Table

Reg	(mm) Start	(mm) Stop	(mm) Centroid	RF	Region Counts	Region CPM	% of Total	% of ROI
Rgn 1	51.5	77.5	64.6	0.084	760.0	760.0	0.90	0.90
Rgn 2	77.5	135.3	105.3	0.824	83322.0	83322.0	98.58	99.10
2 Peaks					84082.0	84082.0	99.48	100.00

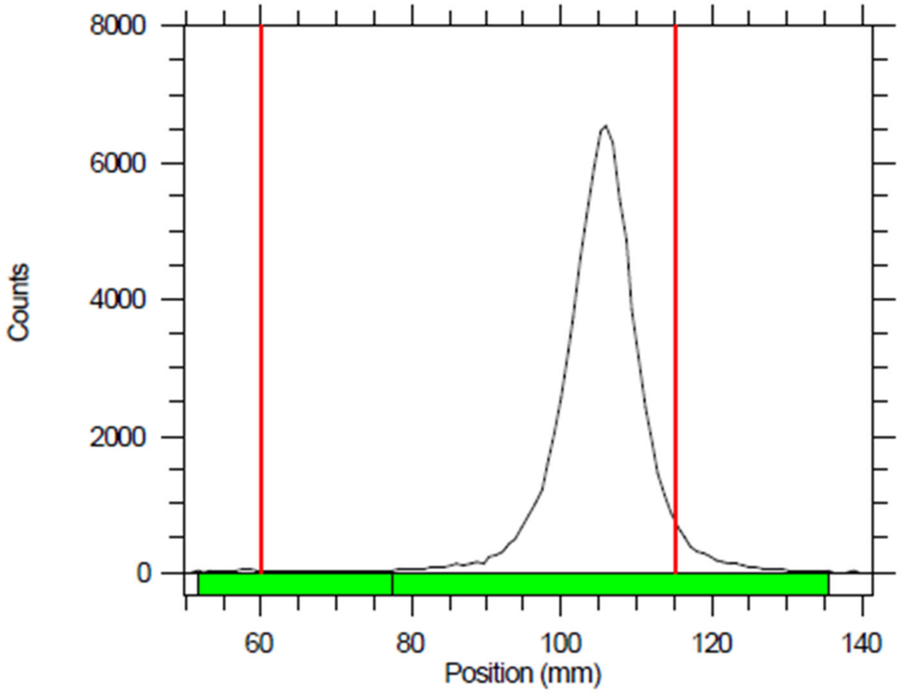

Figure S5: radioTLC of filtered [^{68}Ga]Ga-DOTATOC using 0.22 µm Millex-GS Filter (There was some contamination at the origin of the plate reader, % ROI was used)

Method: TATE File: 072723TOC_S.R001

Instrument Parameters

Method:	TATE	File:	072723TOC_S.R001
Evaluated:	27 Jul 2023 10:45:34	Created:	27 Jul 2023 10:45:34
Evaluation by:	IEW		
Collimator Type:	Hi Efficiency	Width:	10 mm
Elect. Resol:	Normal	Amp. Range:	50 - 2047
Resolution:	256 chan	Chan Size:	0.864 mm
Hi Voltage:	1527 Volts	Chan of Zero mm:	10.3
Run Time:	3.00 min	Max Count	999999
Relative Pos:	0.0 mm		

Comments

Default Method for use in defining other methods

Analysis Parameters

Bkg Subtraction:	none	Origin:	60.0 mm
Normalization:	none	Front:	115.0 mm
Total Counts:	81019.0 (27006.3 CPM)	Region:	50.0 - 140.0 mm
Total File Counts:	81019		

Region Analysis

Definition: Table

Reg	(mm) Start	(mm) Stop	(mm) Centroid	RF	Region Counts	Region CPM	% of Total	% of ROI
Rgn 1	27.4	73.1	58.5	-0.028	42384.0	14128.0	52.31	53.99
Rgn 2	73.1	131.9	93.2	0.604	36119.0	12039.7	44.58	46.01
2 Peaks					78503.0	26167.7	96.89	100.00

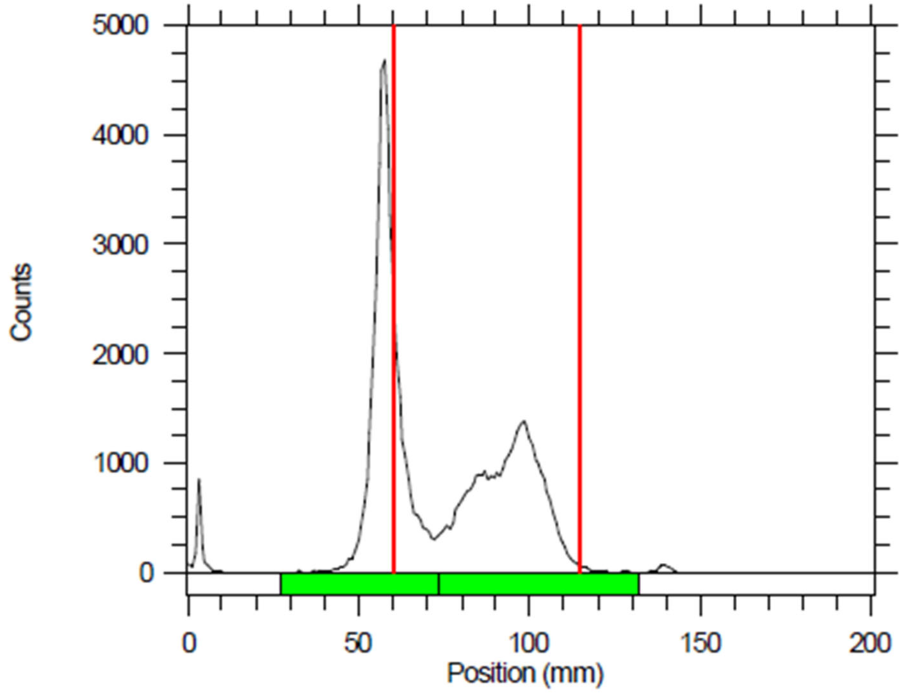

Figure S6: radioTLC of filtered [^{68}Ga]Ga-DOTATOC using 0.22 µm Cathivex-GV Filter following 48 mL air to dry filter

Method: TATE File: 080223-CATH.R001A

Instrument Parameters

Method:	TATE	File:	080223-CATH.R001A
Evaluated:	02 Aug 2023 11:16:19	Created:	02 Aug 2023 10:43:32
Evaluation by:	IEW		
Collimator Type:	Hi Efficiency	Width:	10 mm
Elect. Resol:	Normal	Amp. Range:	50 - 2047
Resolution:	256 chan	Chan Size:	0.864 mm
Hi Voltage:	1528 Volts	Chan of Zero mm:	10.3
Run Time:	1.00 min	Max Count:	999999
Relative Pos:	0.0 mm		

Comments

Default Method for use in defining other methods

Analysis Parameters

Bkg Subtraction:	none	Origin:	60.0 mm
Normalization:	none	Front:	115.0 mm
Total Counts:	157333.0 (157333.0 CPM)	Region:	50.0 - 140.0 mm
Total File Counts:	157333		

Region Analysis

Definition: Table

Reg	(mm) Start	(mm) Stop	(mm) Centroid	RF	Region Counts	Region CPM	% of Total	% of ROI
Rgn 1	50.7	69.7	59.6	-0.008	1557.0	1557.0	0.99	1.00
Rgn 2	68.8	120.6	95.7	0.648	154723.0	154723.0	98.34	99.00
2 Peaks					156280.0	156280.0	99.33	100.00

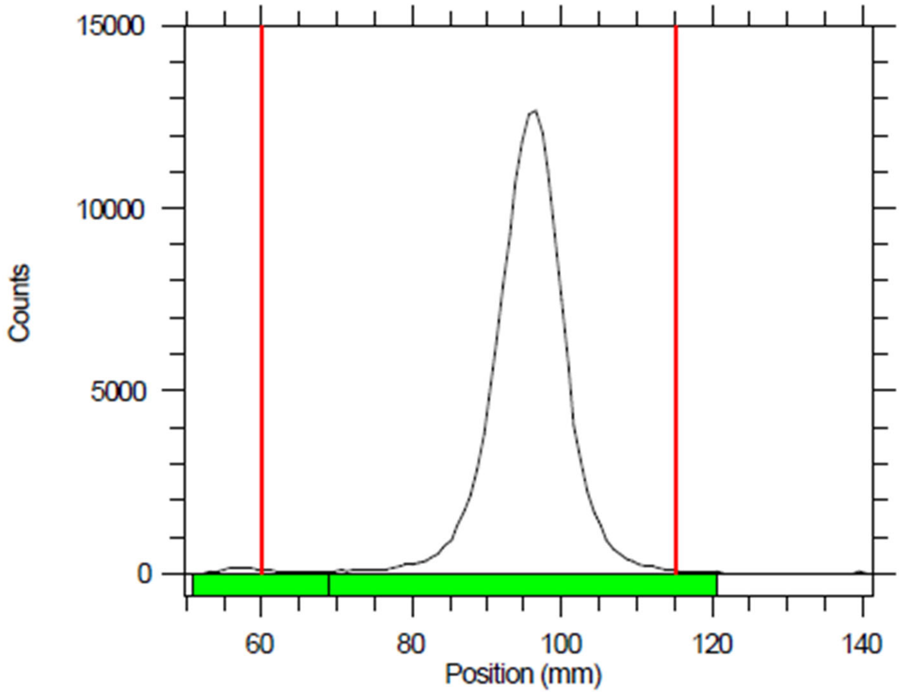

Figure S7: radioTLC of FAPI-04 Validation #1 at initial timepoint (5M NH_4OAc : MeOH (1:1))

Method: AE105 File: 101323-F-5M.R001A

Instrument Parameters

Method:	AE105	File:	101323-F-5M.R001A
Evaluated:	13 Oct 2023 12:34:25	Created:	13 Oct 2023 12:27:18
Evaluation by:	IEW		
Collimator Type:	Hi Efficiency	Width:	10 mm
Elect. Resol:	Normal	Amp. Range:	50 - 2047
Resolution:	256 chan	Chan Size:	0.864 mm
Hi Voltage:	1527 Volts	Chan of Zero mm:	10.3
Run Time:	1.00 min	Max Count	0
Relative Pos:	0.0 mm		

Comments

Default Method for use in defining other methods

Analysis Parameters

Bkg Subtraction:	none	Origin:	60.0 mm
Normalization:	none	Front:	115.0 mm
Total Counts:	67253.0 (67253.0 CPM)	Region:	50.0 - 140.0 mm
Total File Counts:	67253		

Region Analysis

Definition: Table

Reg	(mm) Start	(mm) Stop	(mm) Centroid	RF	Region Counts	Region CPM	% of Total	% of ROI
Rgn 1	46.4	64.5	57.3	-0.048	637.0	637.0	0.95	0.95
Rgn 2	64.5	127.5	90.0	0.546	66366.0	66366.0	98.68	99.05
2 Peaks					67003.0	67003.0	99.63	100.00

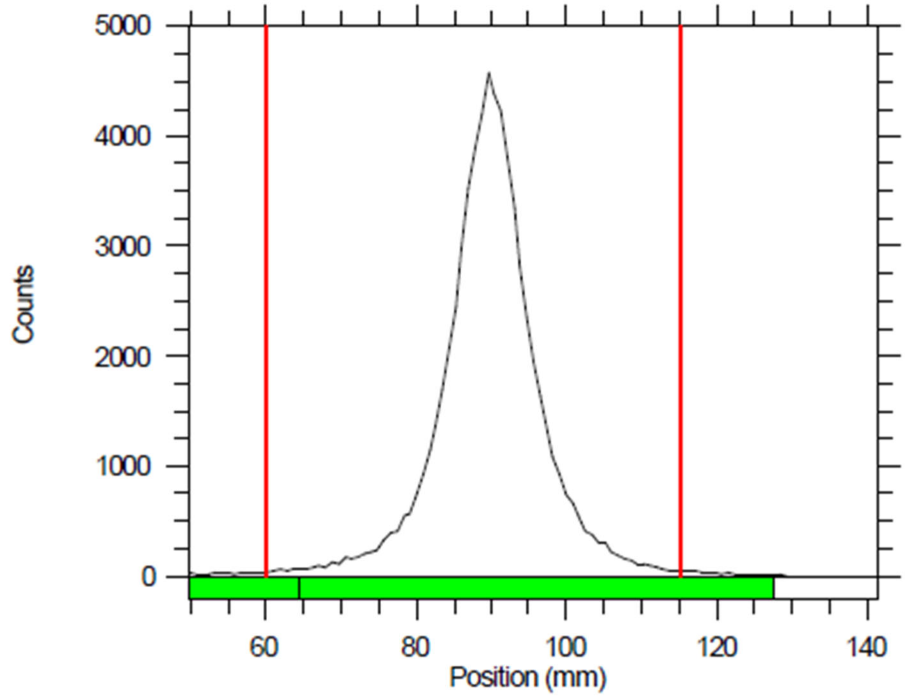

Figure S8: radioTLC of FAPI-04 Validation #2 at initial timepoint (5M NH_4OAc : MeOH (1:1))

Method: AE105 File: 101723FV2-5M.R001A

Instrument Parameters

Method:	AE105	File:	101723FV2-5M.R001A
Evaluated:	17 Oct 2023 10:26:23	Created:	17 Oct 2023 10:20:45
Evaluation by:	IEW		
Collimator Type:	Hi Efficiency	Width:	10 mm
Elect. Resol:	Normal	Amp. Range:	50 - 2047
Resolution:	256 chan	Chan Size:	0.864 mm
Hi Voltage:	1525 Volts	Chan of Zero mm:	10.3
Run Time:	1.00 min	Max Count	0
Relative Pos:	0.0 mm		

Comments

Default Method for use in defining other methods

Analysis Parameters

Bkg Subtraction:	none	Origin:	60.0 mm
Normalization:	none	Front:	115.0 mm
Total Counts:	104457.0 (104457.0 CPM)	Region:	50.0 - 140.0 mm
Total File Counts:	104457		

Region Analysis

Definition: Table

Reg	(mm) Start	(mm) Stop	(mm) Centroid	RF	Region Counts	Region CPM	% of Total	% of ROI
Rgn 1	49.0	65.4	58.9	-0.020	791.0	791.0	0.76	0.76
Rgn 2	65.4	131.0	92.3	0.588	103363.0	103363.0	98.95	99.24
2 Peaks					104154.0	104154.0	99.71	100.00

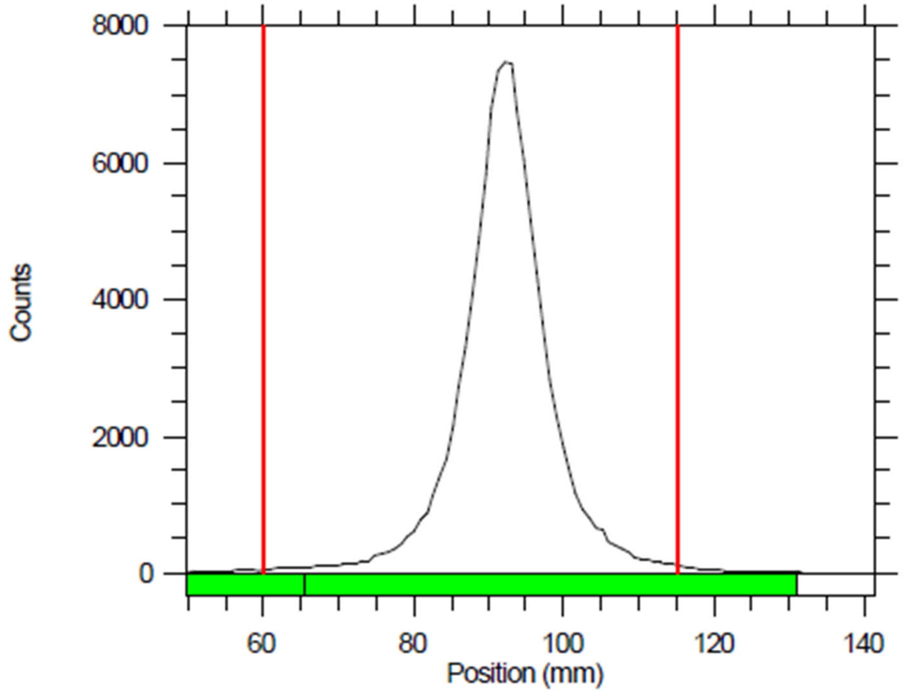

Figure S9: radioTLC of FAPI-04 Validation #3 at initial timepoint (5M NH_4OAc : MeOH (1:1))

Method: AE105 File: 101923FAPI4V3.R001A

Instrument Parameters

Method:	AE105	File:	101923FAPI4V3.R001A
Evaluated:	19 Oct 2023 10:08:33	Created:	19 Oct 2023 10:07:03
Evaluation by:	IEW		
Collimator Type:	Hi Efficiency	Width:	10 mm
Elect. Resol:	Normal	Amp. Range:	50 - 2047
Resolution:	256 chan	Chan Size:	0.864 mm
Hi Voltage:	1527 Volts	Chan of Zero mm:	10.3
Run Time:	1.00 min	Max Count	0
Relative Pos:	0.0 mm		

Comments

Default Method for use in defining other methods

Analysis Parameters

Bkg Subtraction:	none	Origin:	60.0 mm
Normalization:	none	Front:	115.0 mm
Total Counts:	77858.0 (77858.0 CPM)	Region:	50.0 - 140.0 mm
Total File Counts:	77858		

Region Analysis

Definition: Table

Reg	(mm) Start	(mm) Stop	(mm) Centroid	RF	Region Counts	Region CPM	% of Total	% of ROI
Rgn 1	49.0	64.5	58.4	-0.028	564.0	564.0	0.72	0.73
Rgn 2	64.5	132.7	93.4	0.608	76978.0	76978.0	98.87	99.27
2 Peaks					77542.0	77542.0	99.59	100.00

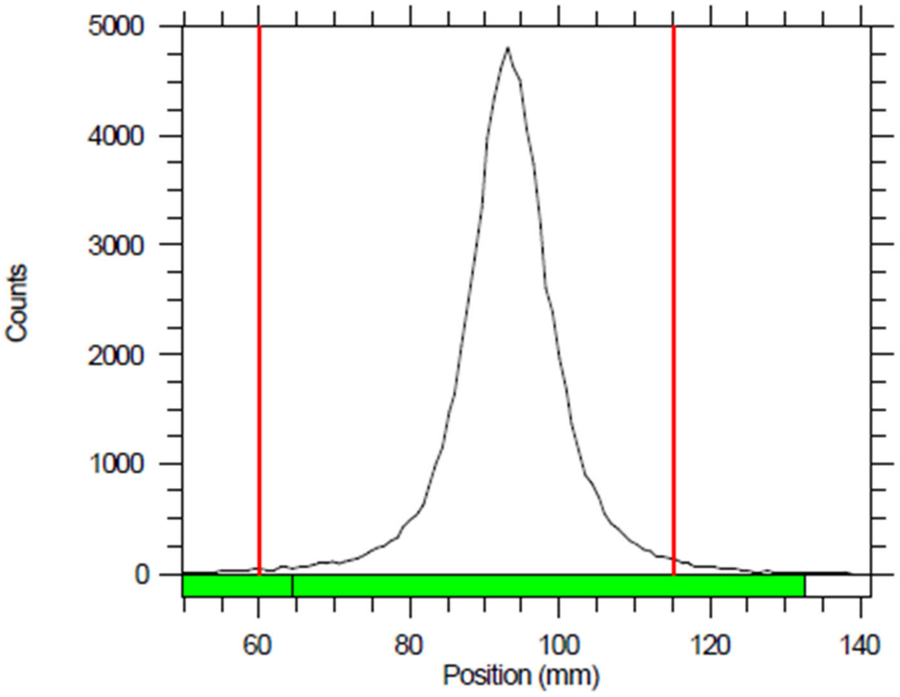

Figure S10: radioHPLC of FAPI-04 Validation #1 at initial timepoint

SHIMADZU LabSolutions

Analysis Report

<Sample Information>

Sample Name	: 101323LunaOmega_dose_imt_2		
Sample ID	: 101323LunaOmega_dose_imt_2		
Data Filename	: 101323LunaOmega_dose_imt_2_0-70B-1minHold-254nm_001.lcd		
Method Filename	: 0-70B-1minHold-254nm.lcm		
Batch Filename	: batch01.lcb		
Vial #	: 1-1	Sample Type	: Unknown
Injection Volume	: 10 uL		
Date Acquired	: 10/13/2023 1:30:38 PM	Acquired by	: System Administrator
Date Processed	: 10/13/2023 2:07:02 PM	Processed by	: System Administrator

<Chromatogram>

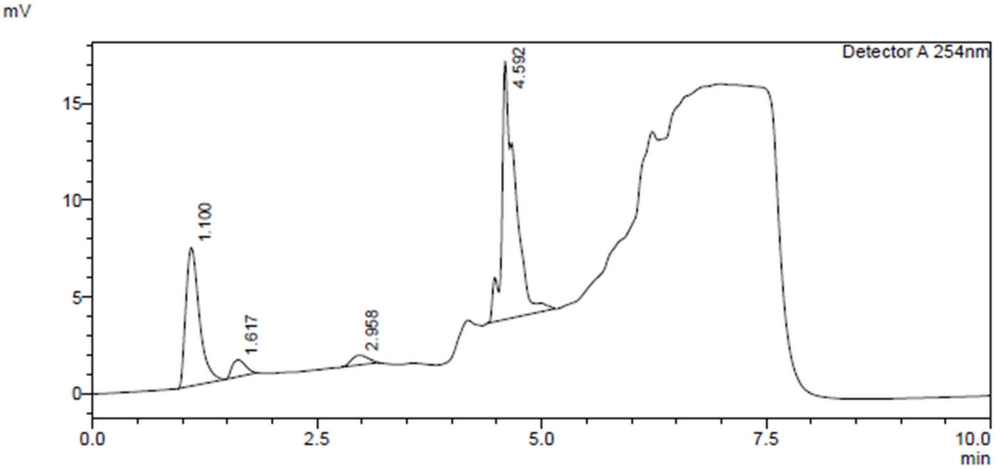

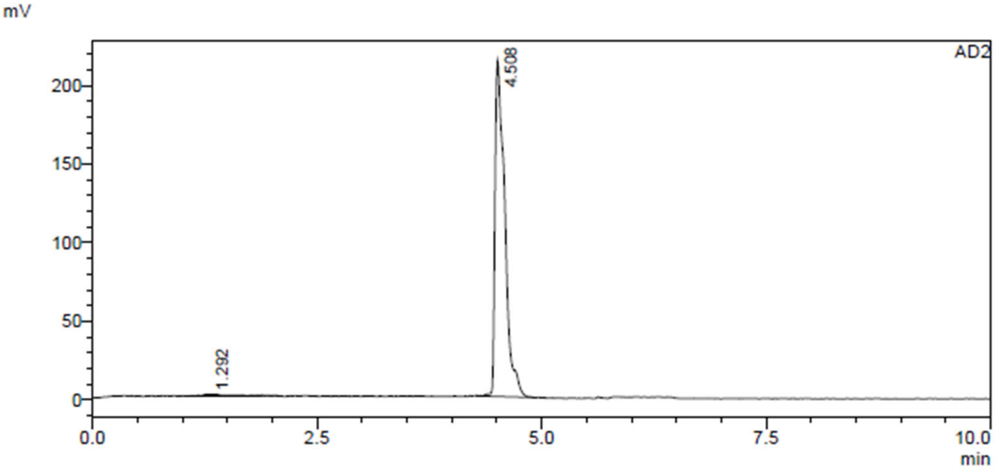

<Peak Table>

Detector A 254nm

Peak#	Ret. Time	Area	Height	Conc.	Unit	Mark	Name
1	1.100	74805	7127	32.695		M	
2	1.617	9015	860	3.940		M	
3	2.958	6346	490	2.774		M	
4	4.592	138628	13340	60.591		M	
Total		228794	21817				

AD2

Peak#	Ret. Time	Area	Height	Conc.	Unit	Mark	Name
1	1.292	23292	1114	1.432		M	
2	4.508	1603391	213654	98.568		M	
Total		1626683	214768				

Figure S11: radioHPLC of FAPI-04 Validation #2 at initial timepoint

SHIMADZU LabSolutions

Analysis Report

<Sample Information>

Sample Name	: 101723_dose		
Sample ID	: 101723_dose		
Data Filename	: 101723_dose_0-70B-1minHold-254nm_003.lcd		
Method Filename	: 0-70B-1minHold-254nm.lcm		
Batch Filename	: batch_001.lcb		
Vial #	: 1-2	Sample Type	: Unknown
Injection Volume	: 10 uL		
Date Acquired	: 10/17/2023 10:02:35 AM	Acquired by	: System Administrator
Date Processed	: 10/17/2023 10:34:00 AM	Processed by	: System Administrator

<Chromatogram>

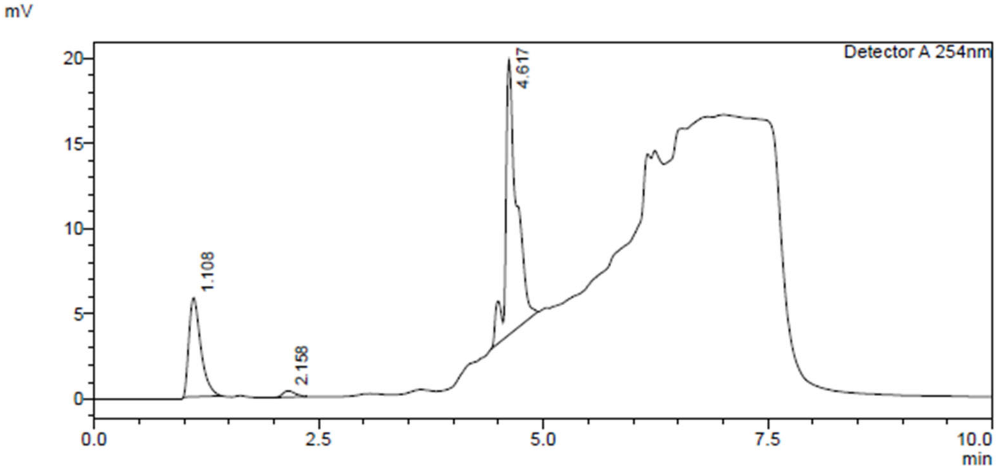

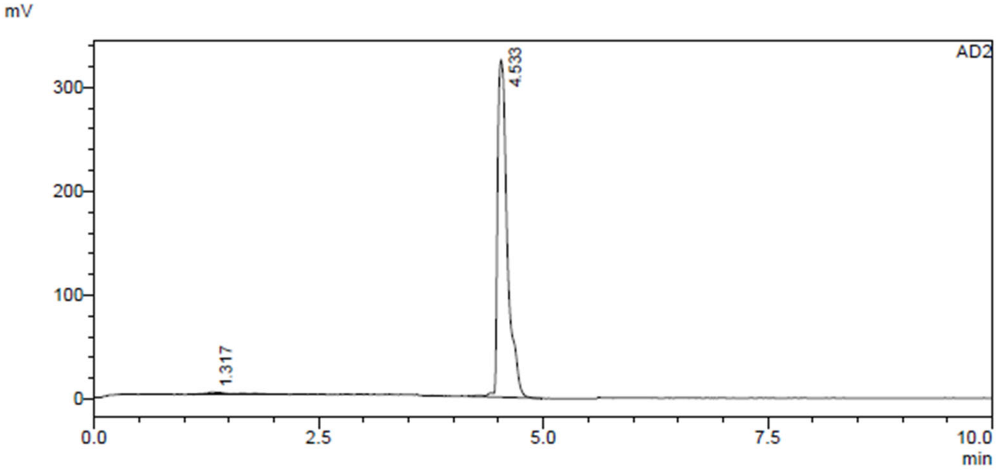

<Peak Table>

Detector A 254nm

Peak#	Ret. Time	Area	Height	Conc.	Unit	Mark	Name
1	1.108	54006	5808	27.437		M	
2	2.158	3497	366	1.777		M	
3	4.617	139334	16084	70.786		M	
Total		196838	22258				

AD2

Peak#	Ret. Time	Area	Height	Conc.	Unit	Mark	Name
1	1.317	43463	2132	1.762		M	
2	4.533	2423841	324141	98.238		M	
Total		2467304	326273				

Figure S12: radioHPLC of FAPI-04 Validation #3 at initial timepoint

SHIMADZU LabSolutions **Analysis Report**

<Sample Information>

Sample Name	: 101923_dose_int		
Sample ID	: 101923_dose_int		
Data Filename	: 101923_dose_int_0-70B-1minHold-254nm_004.lcd		
Method Filename	: 0-70B-1minHold-254nm.lcm		
Batch Filename	: batch_001.lcb		
Vial #	: 1-2	Sample Type	: Unknown
Injection Volume	: 10 uL		
Date Acquired	: 10/19/2023 10:10:41 AM	Acquired by	: System Administrator
Date Processed	: 10/19/2023 10:25:46 AM	Processed by	: System Administrator

<Chromatogram>

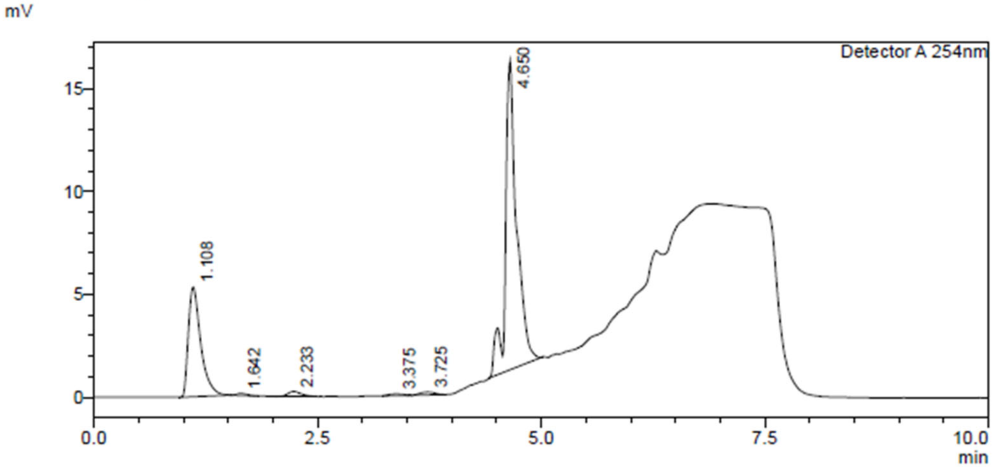

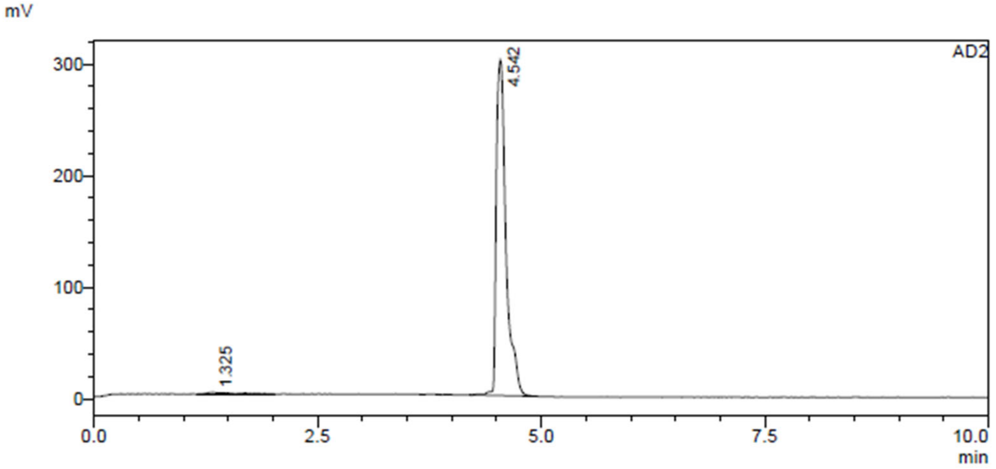

<Peak Table>

Detector A 254nm

Peak#	Ret. Time	Area	Height	Conc.	Unit	Mark	Name
1	1.108	51785	5324	27.290		M	
2	1.642	774	102	0.408		M	
3	2.233	2287	232	1.205		M	
4	3.375	843	81	0.444		M	
5	3.725	1563	141	0.824		M	
6	4.650	132506	15017	69.829		M	
Total		189758	20898				

AD2

Peak#	Ret. Time	Area	Height	Conc.	Unit	Mark	Name
1	1.325	36554	1677	1.600		M	
2	4.542	2248498	300795	98.400		M	
Total		2285051	302472				

Figure S13: radioTLC of FAPI-04 Validation #1 at 4-hr timepoint (5M NH_4OAc : MeOH (1:1))

Method: AE105 File: 101323F_4HR.R001A

Instrument Parameters

Method:	AE105	File:	101323F_4HR.R001A
Evaluated:	13 Oct 2023 16:33:31	Created:	13 Oct 2023 16:28:54
Evaluation by:	IEW		
Collimator Type:	Hi Efficiency	Width:	10 mm
Elect. Resol:	Normal	Amp. Range:	50 - 2047
Resolution:	256 chan	Chan Size:	0.864 mm
Hi Voltage:	1526 Volts	Chan of Zero mm:	10.3
Run Time:	1.00 min	Max Count:	0
Relative Pos:	0.0 mm		

Comments

Default Method for use in defining other methods

Analysis Parameters

Bkg Subtraction:	none	Origin:	60.0 mm
Normalization:	none	Front:	115.0 mm
Total Counts:	97340.0 (97340.0 CPM)	Region:	50.0 - 140.0 mm
Total File Counts:	97340		

Region Analysis

Definition: Table

Reg	(mm) Start	(mm) Stop	(mm) Centroid	RF	Region Counts	Region CPM	% of Total	% of ROI
Rgn 1	51.5	70.5	61.8	0.033	978.0	978.0	1.00	1.02
Rgn 2	70.5	134.5	95.0	0.637	94891.0	94891.0	97.48	98.98
2 Peaks					95869.0	95869.0	98.49	100.00

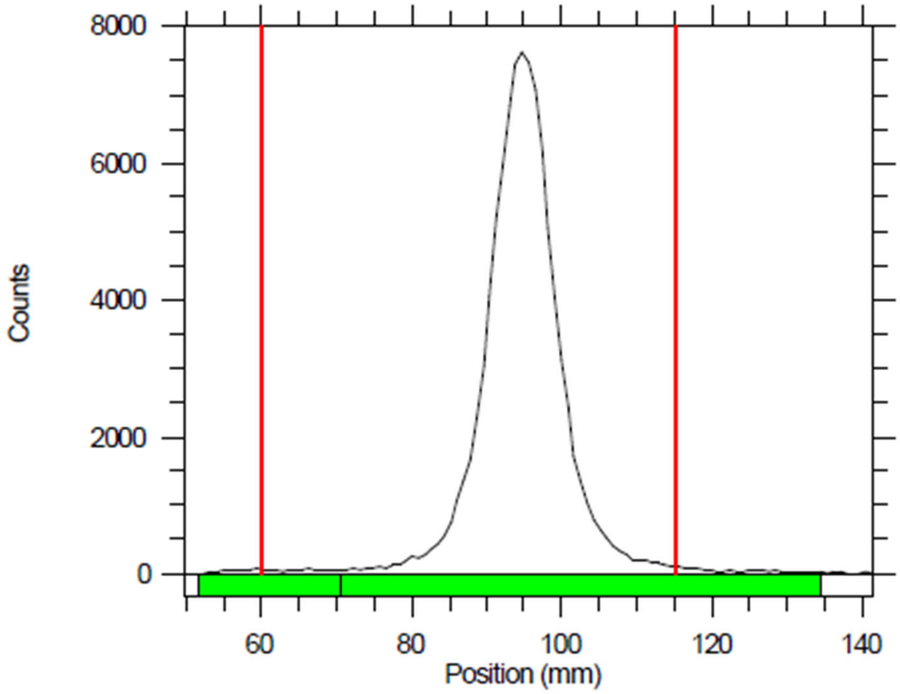

Figure S14: radioTLC of FAPI-04 Validation #2 at 4-hr timepoint (5M NH_4OAc : MeOH (1:1))

Method: AE105 File: 101723FV2-4HR.R001A

Instrument Parameters

Method:	AE105	File:	101723FV2-4HR.R001A
Evaluated:	17 Oct 2023 13:50:34	Created:	17 Oct 2023 13:48:44
Evaluation by:	IEW		
Collimator Type:	Hi Efficiency	Width:	10 mm
Elect. Resol:	Normal	Amp. Range:	50 - 2047
Resolution:	256 chan	Chan Size:	0.864 mm
Hi Voltage:	1526 Volts	Chan of Zero mm:	10.3
Run Time:	1.00 min	Max Count	0
Relative Pos:	0.0 mm		

Comments

Default Method for use in defining other methods

Analysis Parameters

Bkg Subtraction:	none	Origin:	60.0 mm
Normalization:	none	Front:	115.0 mm
Total Counts:	91049.0 (91049.0 CPM)	Region:	50.0 - 140.0 mm
Total File Counts:	91049		

Region Analysis

Definition: Table

Reg	(mm) Start	(mm) Stop	(mm) Centroid	RF	Region Counts	Region CPM	% of Total	% of ROI
Rgn 1	52.4	67.1	60.5	0.008	748.0	748.0	0.82	0.82
Rgn 2	67.1	115.5	91.0	0.563	89943.0	89943.0	98.79	99.18
2 Peaks					90691.0	90691.0	99.61	100.00

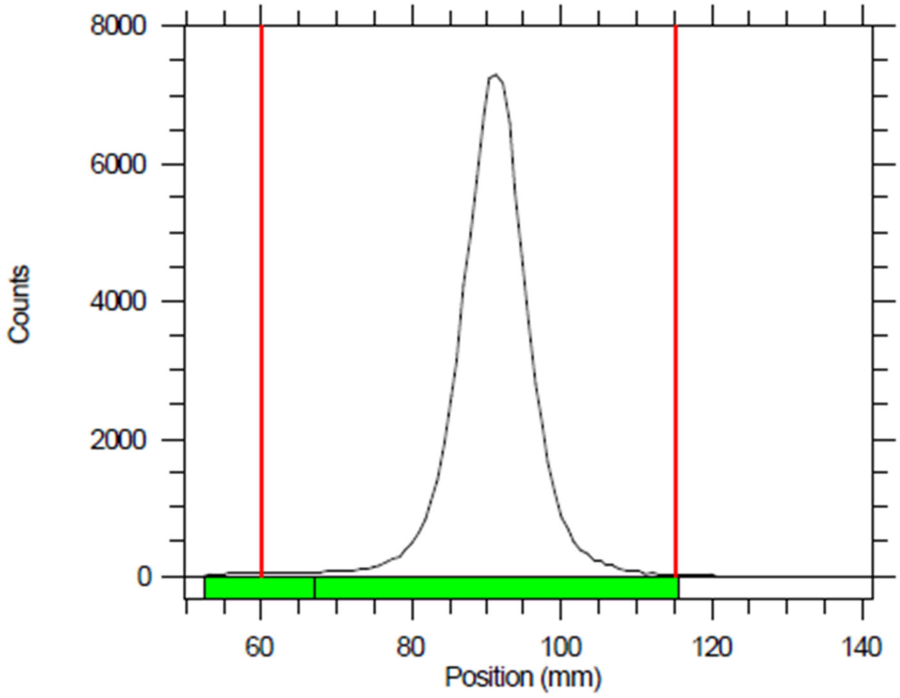

Figure S15: radioTLC of FAPI-04 Validation #3 at 4-hr timepoint (5M NH_4OAc : MeOH (1:1))

Method: AE105 File: 101923FAV3-4H.R001A

Instrument Parameters

Method:	AE105	File:	101923FAV3-4H.R001A
Evaluated:	19 Oct 2023 14:00:42	Created:	19 Oct 2023 13:51:03
Evaluation by:	IEW		
Collimator Type:	Hi Efficiency	Width:	10 mm
Elect. Resol:	Normal	Amp. Range:	50 - 2047
Resolution:	256 chan	Chan Size:	0.864 mm
Hi Voltage:	1527 Volts	Chan of Zero mm:	10.3
Run Time:	1.00 min	Max Count:	0
Relative Pos:	0.0 mm		

Comments

Default Method for use in defining other methods

Analysis Parameters

Bkg Subtraction:	none	Origin:	60.0 mm
Normalization:	none	Front:	115.0 mm
Total Counts:	76168.0 (76168.0 CPM)	Region:	50.0 - 140.0 mm
Total File Counts:	76168		

Region Analysis

Definition: Table

Reg	(mm) Start	(mm) Stop	(mm) Centroid	RF	Region Counts	Region CPM	% of Total	% of ROI
Rgn 1	54.1	71.4	64.1	0.074	917.0	917.0	1.20	1.21
Rgn 2	71.4	114.6	90.3	0.551	74927.0	74927.0	98.37	98.79
2 Peaks					75844.0	75844.0	99.57	100.00

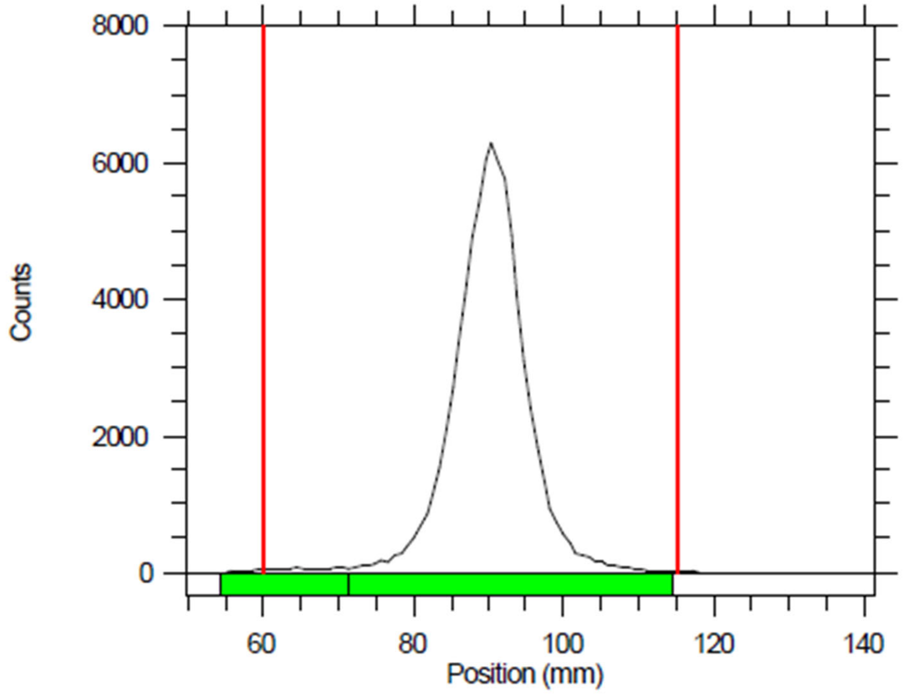

Figure S16: radioHPLC of FAPI-04 Validation #1 at 4-hr timepoint

SHIMADZU LabSolutions **Analysis Report**

<Sample Information>

Sample Name	: 101323LunaOmega_dose_4hr_20mcL		
Sample ID	: 101323LunaOmega_dose_4hr_20mcL		
Data Filename	: 101323LunaOmega_dose_4hr_20mcL_0-70B-1minHold-254nm_001.lcd		
Method Filename	: 0-70B-1minHold-254nm.lcm		
Batch Filename	: batch02.lcb		
Vial #	: 1-1	Sample Type	: Unknown
Injection Volume	: 20 uL		
Date Acquired	: 10/13/2023 4:27:40 PM	Acquired by	: System Administrator
Date Processed	: 10/13/2023 4:44:58 PM	Processed by	: System Administrator

<Chromatogram>

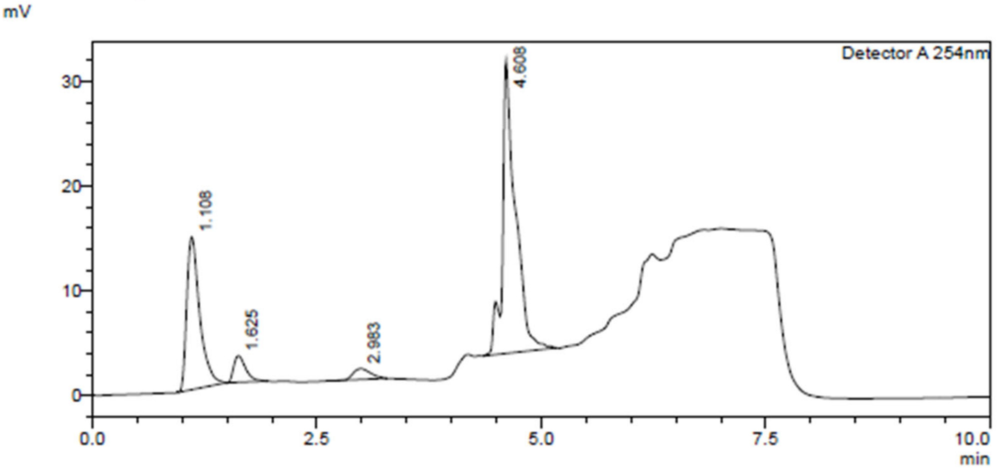

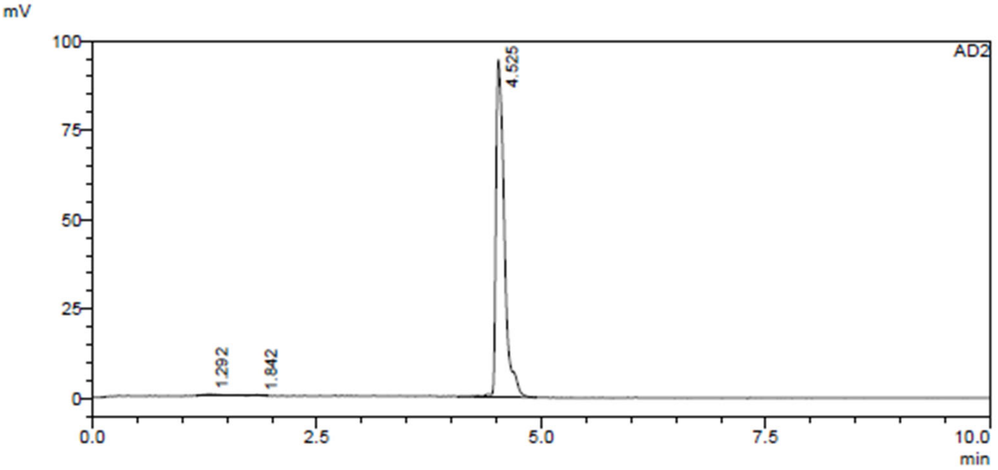

<Peak Table>

Detector A 254nm

Peak#	Ret. Time	Area	Height	Conc.	Unit	Mark	Name
1	1.108	147164	14588	31.453		M	
2	1.625	23824	2545	5.092		M	
3	2.983	13778	1021	2.945		M	
4	4.608	283114	27993	60.510		M	
Total		467880	46146				

AD2

Peak#	Ret. Time	Area	Height	Conc.	Unit	Mark	Name
1	1.292	8184	484	1.339		M	
2	1.842	2167	279	0.355		M	
3	4.525	600882	94268	98.307		M	
Total		611233	95030				

Figure S17: radioHPLC of FAPI-04 Validation #2 at 4-hr timepoint

SHIMADZU LabSolutions

Analysis Report

<Sample Information>

Sample Name	: 101723_dose_5hr_20mcL_inj		
Sample ID	: 101723_dose_5hr_20mcL_inj		
Data Filename	: 101723_dose_5hr_20mcL_inj_0-70B-1minHold-254nm_003.lcd		
Method Filename	: 0-70B-1minHold-254nm.lcm		
Batch Filename	: batch_002.lcb		
Vial #	: 1-16	Sample Type	: Unknown
Injection Volume	: 20 uL		
Date Acquired	: 10/17/2023 3:08:13 PM	Acquired by	: System Administrator
Date Processed	: 10/17/2023 3:20:10 PM	Processed by	: System Administrator

<Chromatogram>

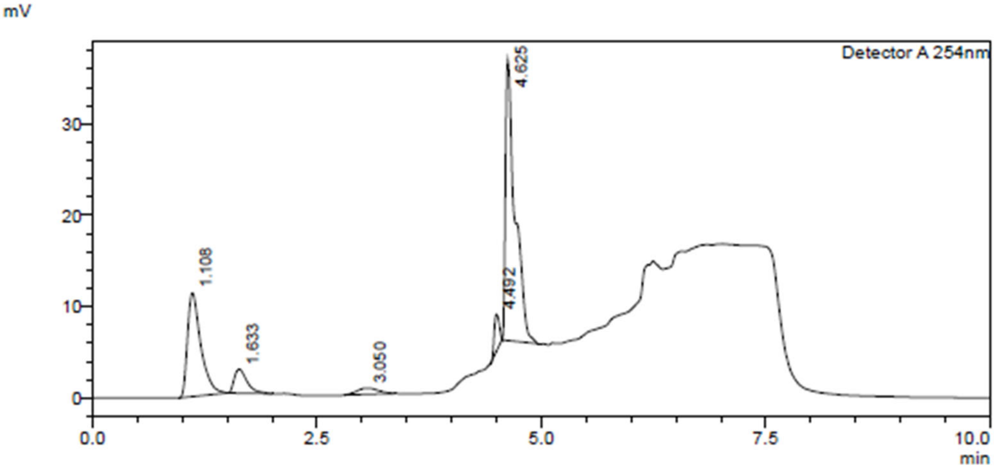

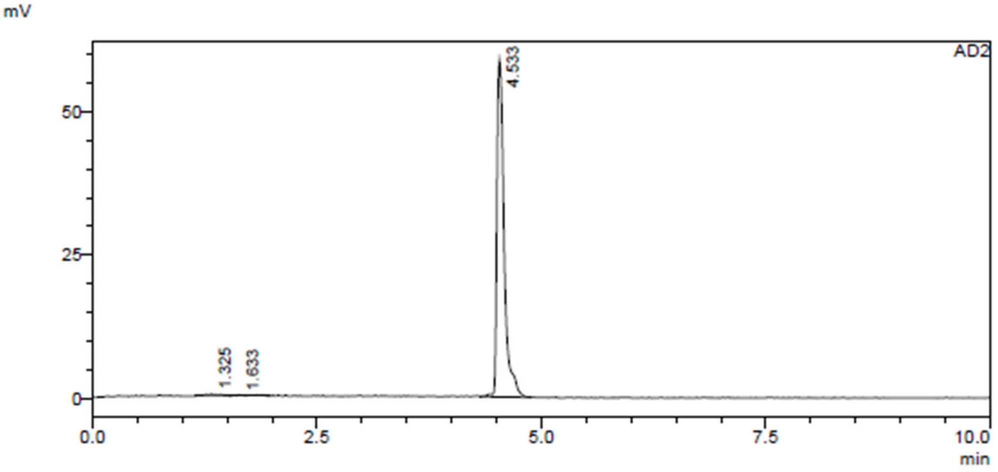

<Peak Table>

Detector A 254nm

Peak#	Ret. Time	Area	Height	Conc.	Unit	Mark	Name
1	1.108	117626	11341	29.465		M	
2	1.633	26288	2625	6.585		M	
3	3.050	11267	679	2.822		M	
4	4.492	14944	4033	3.743		M	
5	4.625	229088	30714	57.385		M	
Total		399212	49393				

AD2

Peak#	Ret. Time	Area	Height	Conc.	Unit	Mark	Name
1	1.325	3471	334	1.063		M	
2	1.633	2373	190	0.727		M	
3	4.533	320746	58701	98.211		M	
Total		326590	59226				

Figure S18: radioHPLC of FAPI-04 Validation #3 at 4-hr timepoint

SHIMADZU LabSolutions

Analysis Report

<Sample Information>

Sample Name	: 101923_dose_4hr_20mcL_inj		
Sample ID	: 101923_dose_4hr_20mcL_inj		
Data Filename	: 101923_dose_4hr_20mcL_inj_0-70B-1minHold-254nm_002.lcd		
Method Filename	: 0-70B-1minHold-254nm.lcm		
Batch Filename	: batch_002.lcb		
Vial #	: 1-1	Sample Type	: Unknown
Injection Volume	: 20 uL		
Date Acquired	: 10/19/2023 1:58:00 PM	Acquired by	: System Administrator
Date Processed	: 10/19/2023 2:14:32 PM	Processed by	: System Administrator

<Chromatogram>

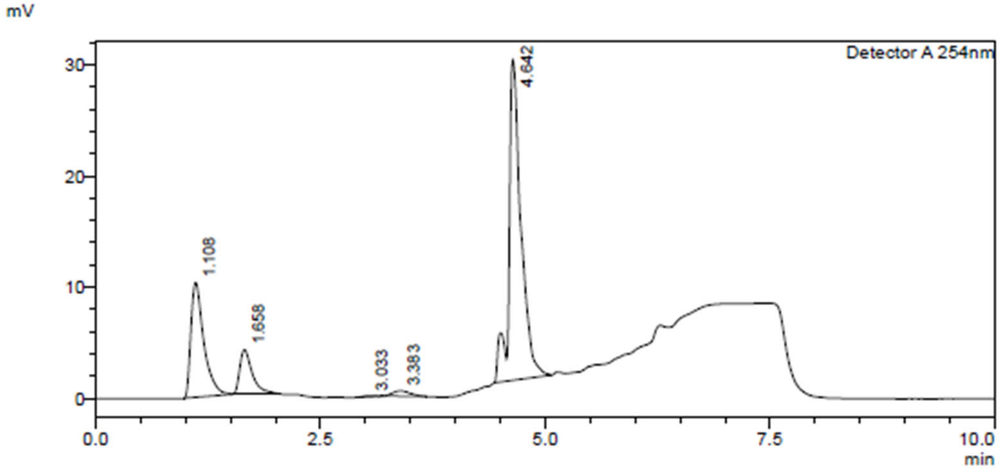

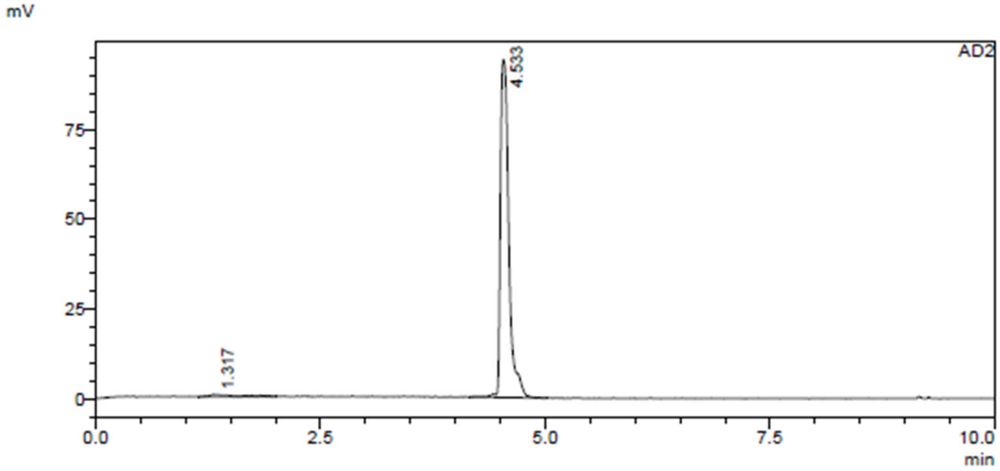

<Peak Table>

Detector A 254nm

Peak#	Ret. Time	Area	Height	Conc.	Unit	Mark	Name
1	1.108	103530	10306	24.638		M	
2	1.658	37876	3954	9.014		M	
3	3.033	907	102	0.216		M	
4	3.383	6594	483	1.569		M	
5	4.642	271302	28808	64.564		M	
Total		420209	43653				

AD2

Peak#	Ret. Time	Area	Height	Conc.	Unit	Mark	Name
1	1.317	10631	585	1.745		M	
2	4.533	598464	93825	98.255		M	
Total		609096	94411				

Figure S19: radioTLC of pentixafor Validation #1 at initial timepoint

Method: IEW File: 103023PTXA4-V1I.R001A

Instrument Parameters

Method:	IEW	File:	103023PTXA4-V1I.R001A
Evaluated:	30 Oct 2023 12:45:32	Created:	30 Oct 2023 12:41:42
Evaluation by:	IEW		
Collimator Type:	Hi Efficiency	Width:	10 mm
Elect. Resol:	Normal	Amp. Range:	50 - 2047
Resolution:	256 chan	Chan Size:	0.864 mm
Hi Voltage:	1526 Volts	Chan of Zero mm:	10.3
Run Time:	1.00 min	Max Count	9999999
Relative Pos:	0.0 mm		

Comments

Default Method for use in defining other methods

Analysis Parameters

Bkg Subtraction:	none	Origin:	60.0 mm
Normalization:	none	Front:	115.0 mm
Total Counts:	98252.0 (98252.0 CPM)	Region:	50.0 - 140.0 mm
Total File Counts:	98252		

Region Analysis

Definition: Table

Reg	(mm) Start	(mm) Stop	(mm) Centroid	RF	Region Counts	Region CPM	% of Total	% of ROI
Rgn 1	50.7	74.0	65.6	0.102	1227.0	1227.0	1.25	1.25
Rgn 2	74.0	130.1	96.8	0.669	96644.0	96644.0	98.36	98.75
2 Peaks					97871.0	97871.0	99.61	100.00

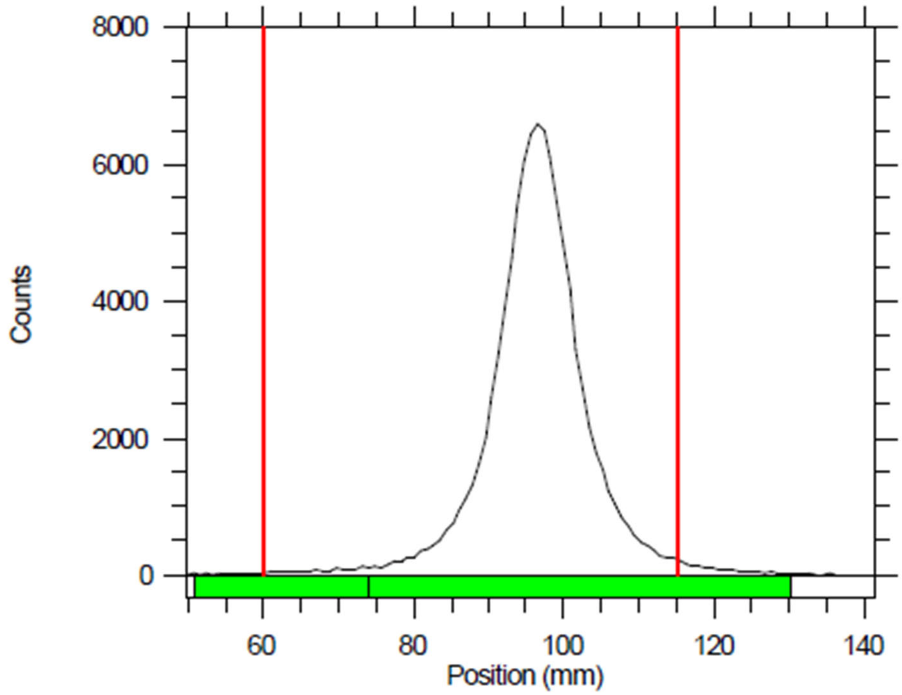

Figure S20: radioTLC of pentixafor Validation #2 at initial timepoint

Method: IEW File: 103123PTXA4-V2.R001A

Instrument Parameters

Method:	IEW	File:	103123PTXA4-V2.R001A
Evaluated:	31 Oct 2023 11:32:21	Created:	31 Oct 2023 11:29:26
Evaluation by:	IEW		
Collimator Type:	Hi Efficiency	Width:	10 mm
Elect. Resol:	Normal	Amp. Range:	50 - 2047
Resolution:	256 chan	Chan Size:	0.864 mm
Hi Voltage:	1526 Volts	Chan of Zero mm:	10.3
Run Time:	1.00 min	Max Count	9999999
Relative Pos:	0.0 mm		

Comments

Default Method for use in defining other methods

Analysis Parameters

Bkg Subtraction:	none	Origin:	60.0 mm
Normalization:	none	Front:	115.0 mm
Total Counts:	100941.0 (100941.0 CPM)	Region:	50.0 - 140.0 mm
Total File Counts:	100941		

Region Analysis

Definition: Table

Reg	(mm) Start	(mm) Stop	(mm) Centroid	RF	Region Counts	Region CPM	% of Total	% of ROI
Rgn 1	52.4	69.7	62.4	0.043	769.0	769.0	0.76	0.76
Rgn 2	69.7	134.5	96.4	0.662	99828.0	99828.0	98.90	99.24
2 Peaks					100597.0	100597.0	99.66	100.00

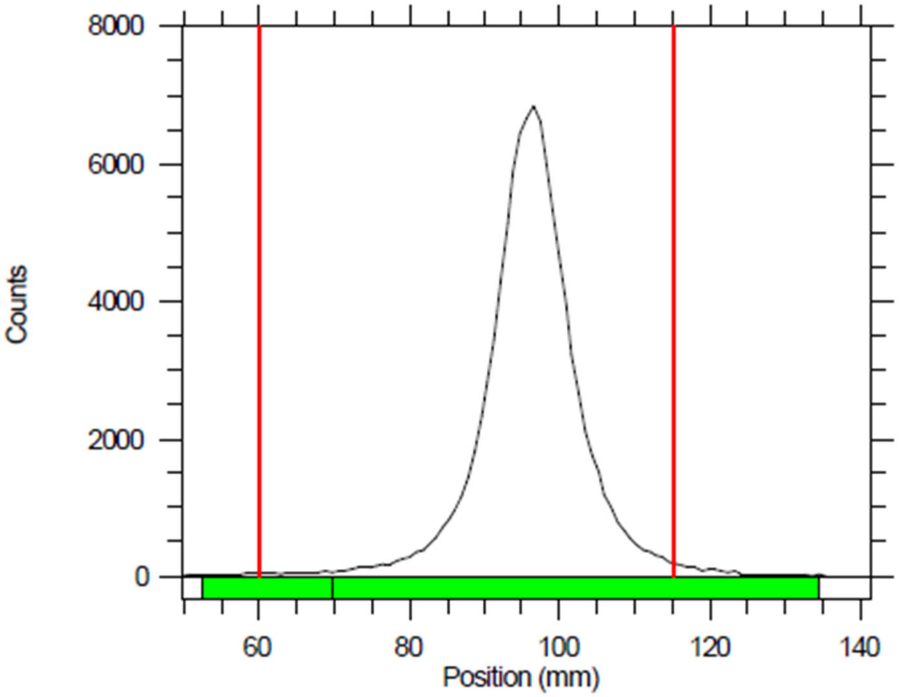

Figure S21: radioTLC of pentixafor Validation #3 at initial timepoint

Method: IEW File: 110123PTXA4-V3.R001A

Instrument Parameters

Method:	IEW	File:	110123PTXA4-V3.R001A
Evaluated:	01 Nov 2023 09:39:18	Created:	01 Nov 2023 09:36:42
Evaluation by:	IEW		
Collimator Type:	Hi Efficiency	Width:	10 mm
Elect. Resol:	Normal	Amp. Range:	50 - 2047
Resolution:	256 chan	Chan Size:	0.864 mm
Hi Voltage:	1527 Volts	Chan of Zero mm:	10.3
Run Time:	1.00 min	Max Count:	9999999
Relative Pos:	0.0 mm		

Comments

Default Method for use in defining other methods

Analysis Parameters

Bkg Subtraction:	none	Origin:	60.0 mm
Normalization:	none	Front:	115.0 mm
Total Counts:	92318.0 (92318.0 CPM)	Region:	50.0 - 140.0 mm
Total File Counts:	92318		

Region Analysis

Definition: Table

Reg	(mm) Start	(mm) Stop	(mm) Centroid	RF	Region Counts	Region CPM	% of Total	% of ROI
Rgn 1	55.9	74.0	66.0	0.109	661.0	661.0	0.72	0.72
Rgn 2	74.0	132.7	101.1	0.748	91242.0	91242.0	98.83	99.28
2 Peaks					91903.0	91903.0	99.55	100.00

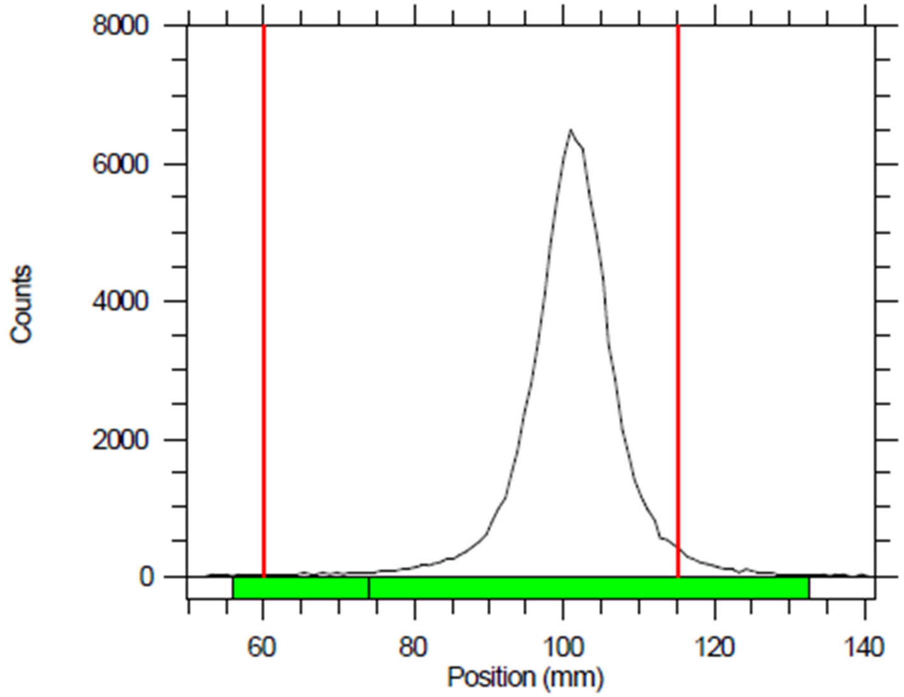

Figure S22: radioHPLC of pentixafor Validation #1 at initial timepoint

SHIMADZU LabSolutions **Analysis Report**

<Sample Information>

Sample Name	: 103023_dose_int_b		
Sample ID	: 103023_dose_int_b		
Data Filename	: 103023_dose_int_b_0-70B-1minHold-254nm_001.lcd		
Method Filename	: 0-70B-1minHold-254nm.lcm		
Batch Filename	: batch_002.lcb		
Vial #	: 1-2	Sample Type	: Unknown
Injection Volume	: 10 uL		
Date Acquired	: 10/30/2023 12:47:05 PM	Acquired by	: System Administrator
Date Processed	: 10/30/2023 1:04:06 PM	Processed by	: System Administrator

<Chromatogram>

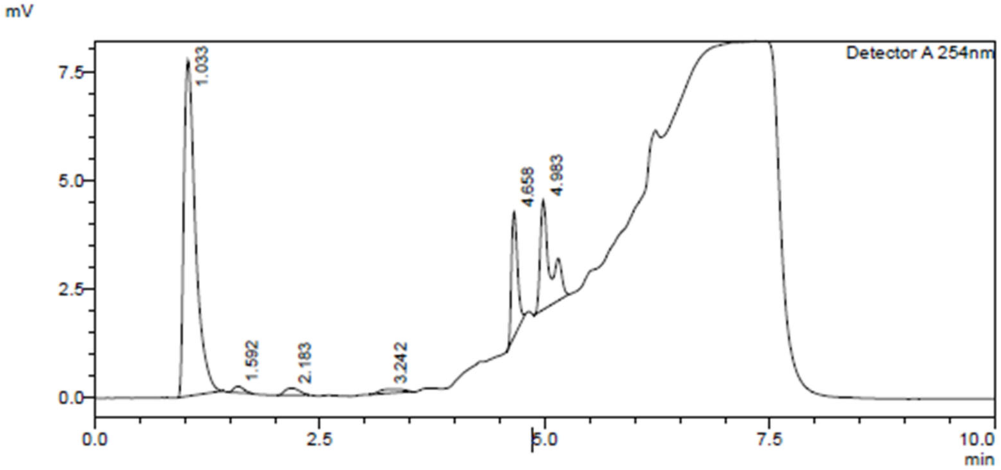

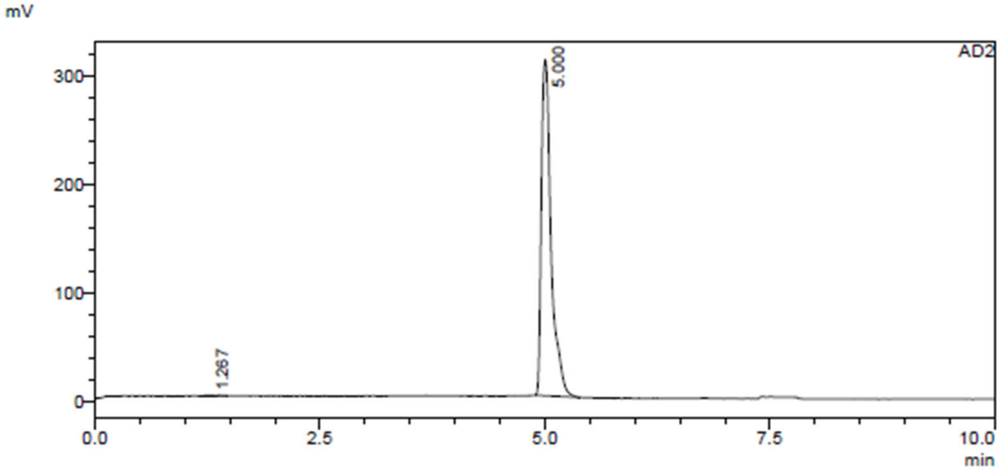

<Peak Table>

Detector A 254nm

Peak#	Ret. Time	Area	Height	Conc.	Unit	Mark	Name
1	1.033	69660	7716	65.439		M	
2	1.592	1114	143	1.046		M	
3	2.183	1750	161	1.644		M	
4	3.242	1560	93	1.465		M	
5	4.658	13165	2849	12.367		M	
6	4.983	19203	2504	18.039		M	
Total		106452	13467				

AD2

Peak#	Ret. Time	Area	Height	Conc.	Unit	Mark	Name
1	1.267	7486	860	0.326		M	
2	5.000	2286220	309766	99.674		M	
Total		2293706	310626				

Figure S23: radioHPLC of pentixafor Validation #2 at initial timepoint

SHIMADZU LabSolutions **Analysis Report**

<Sample Information>

Sample Name	: 103123_dose		
Sample ID	: 103123_dose		
Data Filename	: 103123_dose_0-70B-1minHold-254nm_004.lcd		
Method Filename	: 0-70B-1minHold-254nm.lcm		
Batch Filename	: batch_001.lcb		
Vial #	: 1-2	Sample Type	: Unknown
Injection Volume	: 10 uL		
Date Acquired	: 10/31/2023 11:37:07 AM	Acquired by	: System Administrator
Date Processed	: 10/31/2023 11:55:32 AM	Processed by	: System Administrator

<Chromatogram>

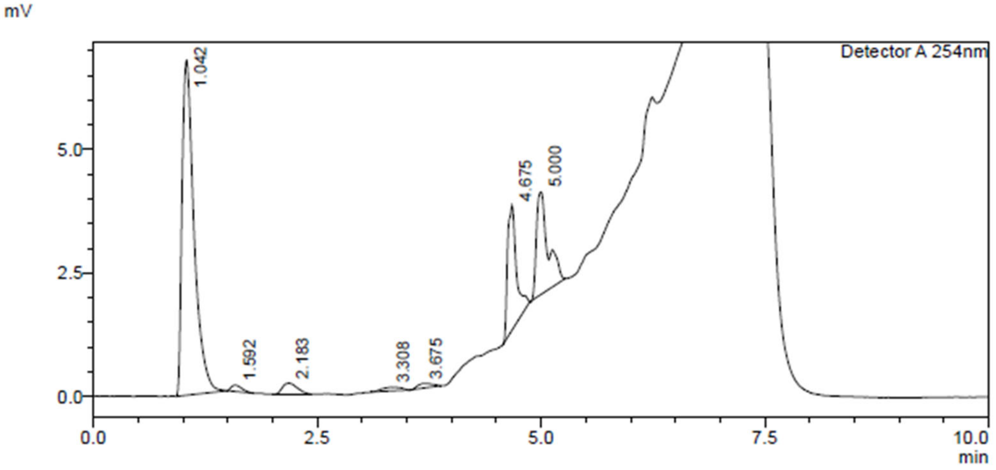

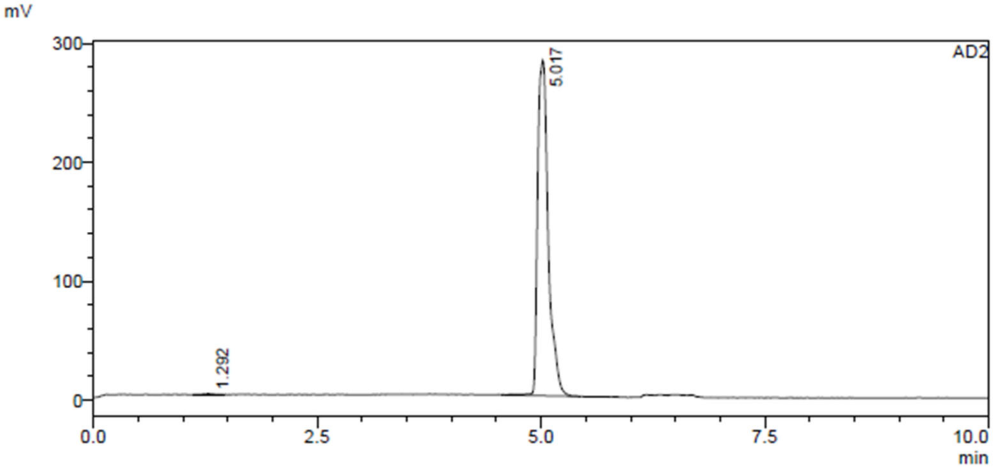

<Peak Table>

Detector A 254nm

Peak#	Ret. Time	Area	Height	Conc.	Unit	Mark	Name
1	1.042	63580	6782	60.367		M	
2	1.592	1069	127	1.015		M	
3	2.183	2544	219	2.415		M	
4	3.308	1146	76	1.088		M	
5	3.675	1023	93	0.972		M	
6	4.675	17527	2527	16.641		M	
7	5.000	18434	2079	17.502		M	
Total		105323	11903				

AD2

Peak#	Ret. Time	Area	Height	Conc.	Unit	Mark	Name
1	1.292	7508	816	0.324		M	
2	5.017	2307169	281858	99.676		M	
Total		2314677	282674				

Figure S24: radioHPLC of pentixafor Validation #3 at initial timepoint

SHIMADZU LabSolutions **Analysis Report**

<Sample Information>

Sample Name	: 110123_dose_int_10mcl_inj		
Sample ID	: 110123_dose_int_10mcl_inj		
Data Filename	: 110123_dose_int_10mcl_inj_0-70B-1minHold-254nm_001.lcd		
Method Filename	: 0-70B-1minHold-254nm.lcm		
Batch Filename	: batch_002.lcb		
Vial #	: 1-1	Sample Type	: Unknown
Injection Volume	: 10 uL		
Date Acquired	: 11/1/2023 10:01:37 AM	Acquired by	: System Administrator
Date Processed	: 11/1/2023 10:30:40 AM	Processed by	: System Administrator

<Chromatogram>

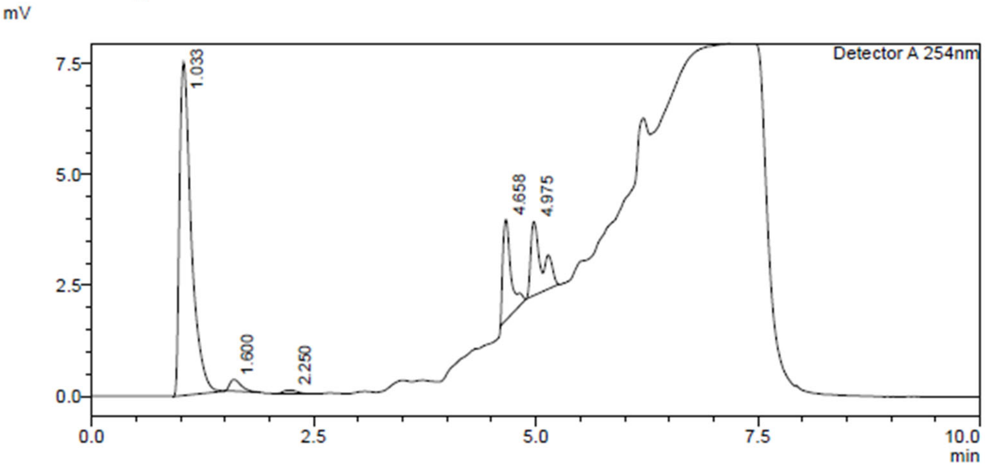

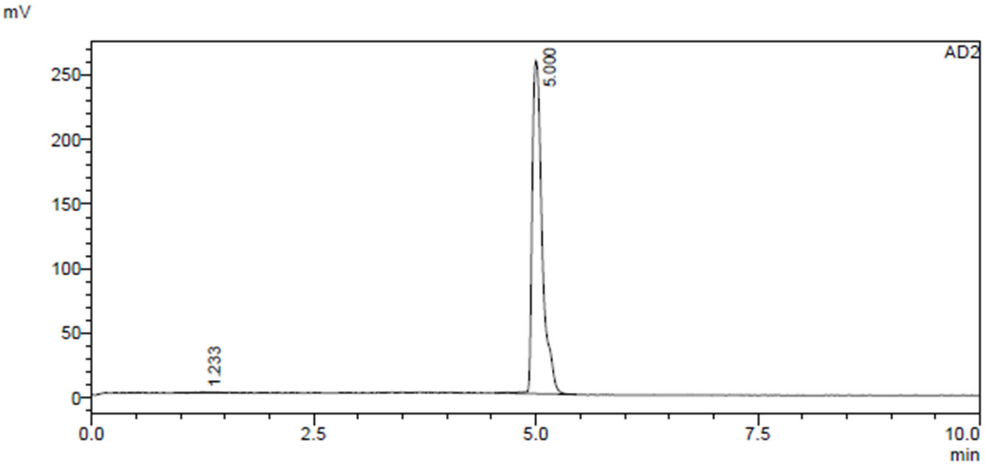

<Peak Table>

Detector A 254nm

Peak#	Ret. Time	Area	Height	Conc.	Unit	Mark	Name
1	1.033	69673	7518	68.448		M	
2	1.600	2351	262	2.309		M	
3	2.250	679	69	0.667		M	
4	4.658	14490	2275	14.235		M	
5	4.975	14597	1668	14.341		M	
Total		101790	11792				

AD2

Peak#	Ret. Time	Area	Height	Conc.	Unit	Mark	Name
1	1.233	7000	680	0.352		M	
2	5.000	1984259	258107	99.648		M	
Total		1991259	258787				

Figure S25: radioTLC of pentixafor Validation #1 at 4-hr timepoint

Method: IEW File: 103023PT_V1_4H.R001A

Instrument Parameters

Method:	IEW	File:	103023PT_V1_4H.R001A
Evaluated:	30 Oct 2023 16:06:00	Created:	30 Oct 2023 16:03:59
Evaluation by:	IEW		
Collimator Type:	Hi Efficiency	Width:	10 mm
Elect. Resol:	Normal	Amp. Range:	50 - 2047
Resolution:	256 chan	Chan Size:	0.864 mm
Hi Voltage:	1523 Volts	Chan of Zero mm:	10.3
Run Time:	1.00 min	Max Count:	9999999
Relative Pos:	0.0 mm		

Comments

Default Method for use in defining other methods

Analysis Parameters

Bkg Subtraction:	none	Origin:	60.0 mm
Normalization:	none	Front:	115.0 mm
Total Counts:	101311.0 (101311.0 CPM)	Region:	50.0 - 140.0 mm
Total File Counts:	101311		

Region Analysis

Definition: Table

Reg	(mm) Start	(mm) Stop	(mm) Centroid	RF	Region Counts	Region CPM	% of Total	% of ROI
Rgn 1	53.3	74.0	64.0	0.073	1409.0	1409.0	1.39	1.39
Rgn 2	74.0	118.9	95.7	0.649	99603.0	99603.0	98.31	98.61
2 Peaks					101012.0	101012.0	99.70	100.00

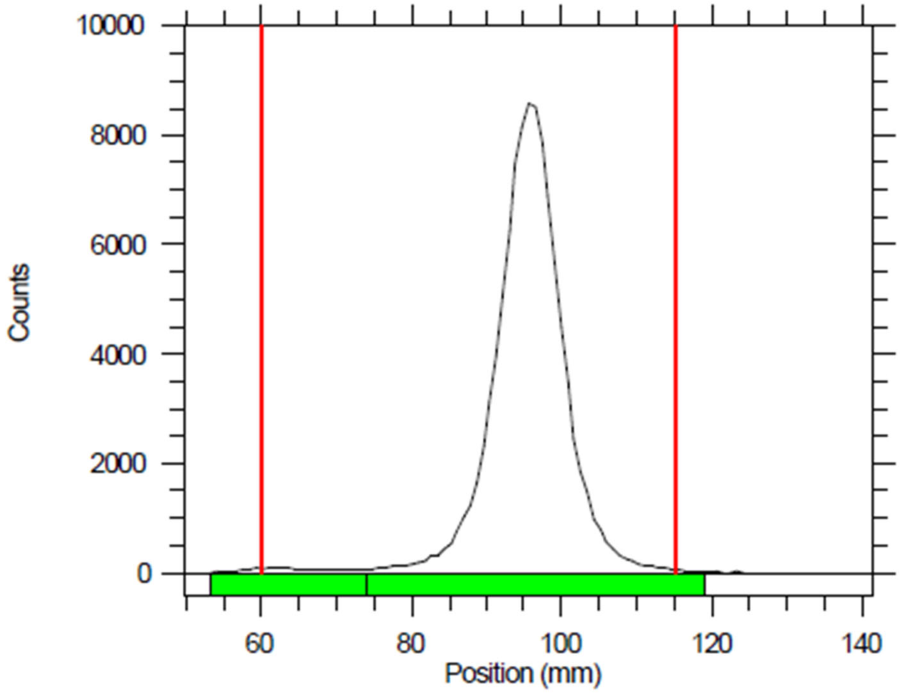

Figure S26: radioTLC of pentixafor Validation #2 at 4-hr timepoint

Method: IEW File: 103123PT_V2_4H.R001A

Instrument Parameters

Method:	IEW	File:	103123PT_V2_4H.R001A
Evaluated:	31 Oct 2023 14:56:25	Created:	31 Oct 2023 14:37:29
Evaluation by:	IEW		
Collimator Type:	Hi Efficiency	Width:	10 mm
Elect. Resol:	Normal	Amp. Range:	50 - 2047
Resolution:	256 chan	Chan Size:	0.864 mm
Hi Voltage:	1527 Volts	Chan of Zero mm:	10.3
Run Time:	1.00 min	Max Count	9999999
Relative Pos:	0.0 mm		

Comments

Default Method for use in defining other methods

Analysis Parameters

Bkg Subtraction:	none	Origin:	60.0 mm
Normalization:	none	Front:	115.0 mm
Total Counts:	75561.0 (75561.0 CPM)	Region:	50.0 - 140.0 mm
Total File Counts:	75561		

Region Analysis

Definition: Table

Reg	(mm) Start	(mm) Stop	(mm) Centroid	RF	Region Counts	Region CPM	% of Total	% of ROI
Rgn 1	51.5	69.7	61.4	0.025	777.0	777.0	1.03	1.03
Rgn 2	69.7	122.4	93.9	0.617	74576.0	74576.0	98.70	98.97
2 Peaks					75353.0	75353.0	99.72	100.00

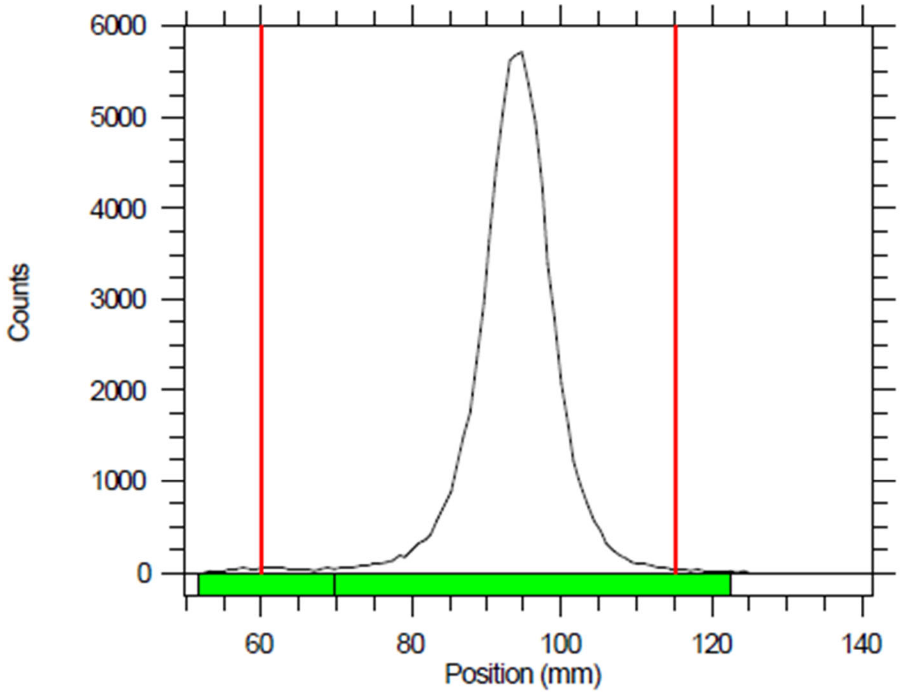

Figure S27: radioTLC of pentixafor Validation #3 at 4-hr timepoint

Method: IEW File: 110123PT-V3-4HB.R001A

Instrument Parameters

Method:	IEW	File:	110123PT-V3-4HB.R001A
Evaluated:	01 Nov 2023 13:15:37	Created:	01 Nov 2023 13:13:26
Evaluation by:	IEW		
Collimator Type:	Hi Efficiency	Width:	10 mm
Elect. Resol:	Normal	Amp. Range:	50 - 2047
Resolution:	256 chan	Chan Size:	0.864 mm
Hi Voltage:	1526 Volts	Chan of Zero mm:	10.3
Run Time:	1.00 min	Max Count	9999999
Relative Pos:	0.0 mm		

Comments

Default Method for use in defining other methods

Analysis Parameters

Bkg Subtraction:	none	Origin:	60.0 mm
Normalization:	none	Front:	115.0 mm
Total Counts:	73311.0 (73311.0 CPM)	Region:	50.0 - 140.0 mm
Total File Counts:	73311		

Region Analysis

Definition: Table

Reg	(mm) Start	(mm) Stop	(mm) Centroid	RF	Region Counts	Region CPM	% of Total	% of ROI
Rgn 1	53.3	81.8	68.9	0.162	1022.0	1022.0	1.39	1.40
Rgn 2	81.8	129.3	102.7	0.776	72054.0	72054.0	98.29	98.60
2 Peaks					73076.0	73076.0	99.68	100.00

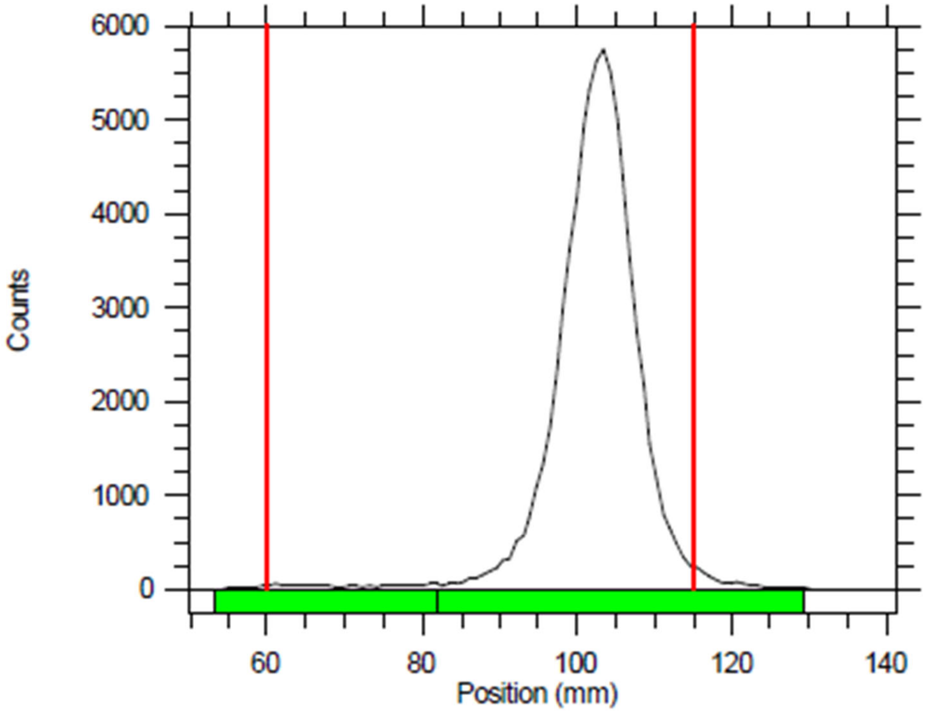

Figure S28: radioHPLC of pentixafor Validation #1 at 4-hr timepoint

SHIMADZU LabSolutions

Analysis Report

<Sample Information>

Sample Name	: 103023_dose_4hr_20mcL_inj		
Sample ID	: 103023_dose_4hr_20mcL_inj		
Data Filename	: 103023_dose_4hr_20mcL_inj_0-70B-1minHold-254nm_002.lcd		
Method Filename	: 0-70B-1minHold-254nm.lcm		
Batch Filename	: batch_004.lcb		
Vial #	: 1-1	Sample Type	: Unknown
Injection Volume	: 20 uL		
Date Acquired	: 10/30/2023 4:07:23 PM	Acquired by	: System Administrator
Date Processed	: 10/30/2023 4:20:05 PM	Processed by	: System Administrator

<Chromatogram>

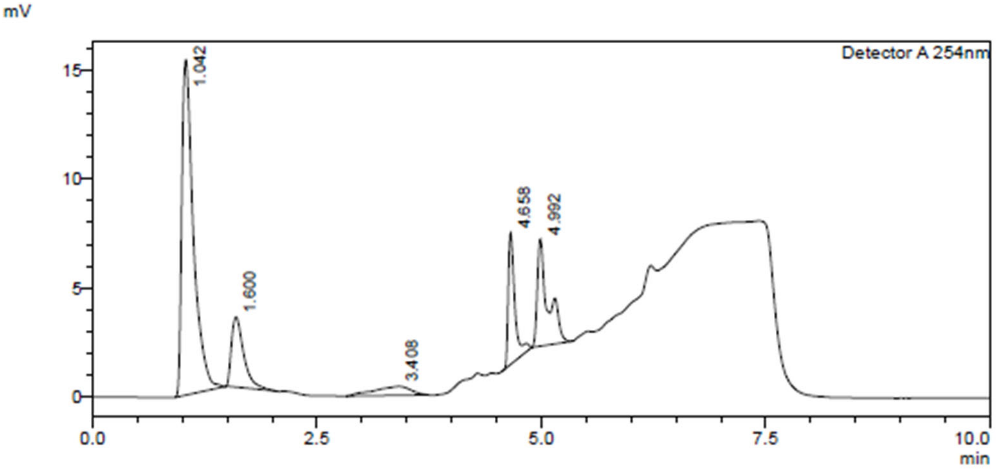

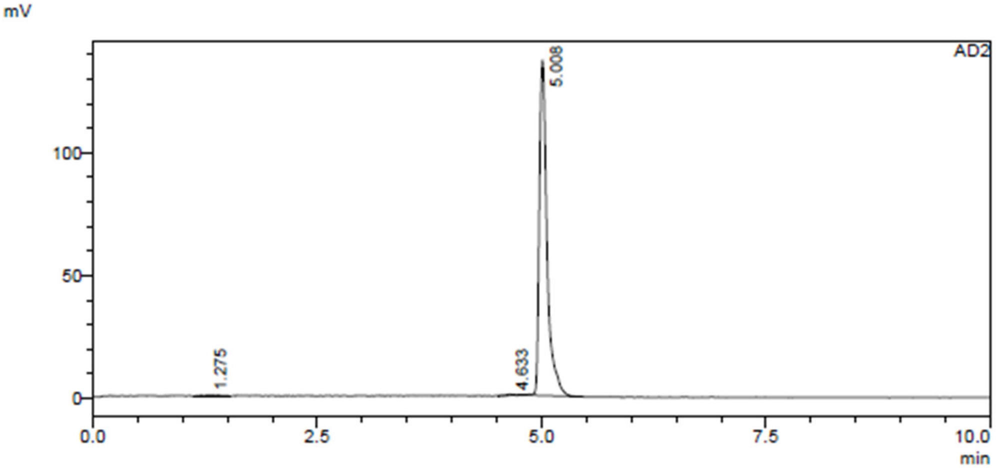

<Peak Table>

Detector A 254nm

Peak#	Ret. Time	Area	Height	Conc.	Unit	Mark	Name
1	1.042	138894	15349	55.136		M	
2	1.600	30766	3208	12.213		M	
3	3.408	11627	407	4.616		M	
4	4.658	30619	6005	12.155		M	
5	4.992	40003	4907	15.880		M	
Total		251910	29876				

AD2

Peak#	Ret. Time	Area	Height	Conc.	Unit	Mark	Name
1	1.275	4280	339	0.528		M	
2	4.633	2188	423	0.270		M	
3	5.008	804783	136871	99.203		M	
Total		811251	137633				

Figure S29: radioHPLC of pentixafor Validation #2 at 4-hr timepoint

SHIMADZU LabSolutions **Analysis Report**

<Sample Information>

Sample Name	: 103123_dose_4hr_20mcl_inj		
Sample ID	: 103123_dose_4hr_20mcl_inj		
Data Filename	: 103123_dose_4hr_20mcl_inj_0-70B-1minHold-254nm_002.lcd		
Method Filename	: 0-70B-1minHold-254nm.lcm		
Batch Filename	: batch_002.lcb		
Vial #	: 1-1	Sample Type	: Unknown
Injection Volume	: 20 uL		
Date Acquired	: 10/31/2023 2:37:41 PM	Acquired by	: System Administrator
Date Processed	: 10/31/2023 2:55:50 PM	Processed by	: System Administrator

<Chromatogram>

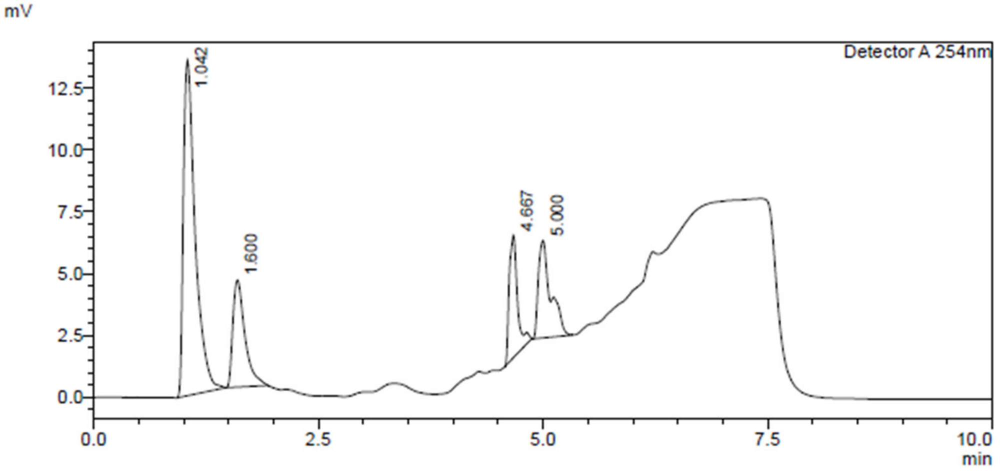

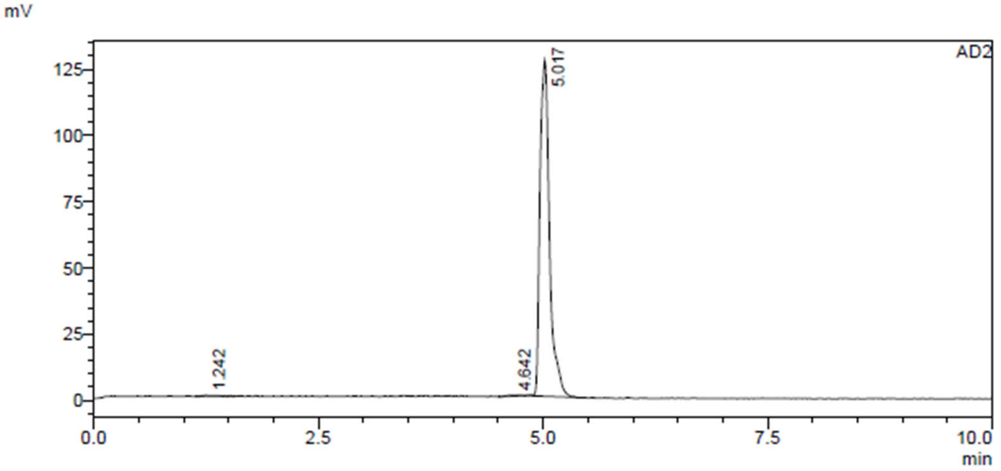

<Peak Table>

Detector A 254nm

Peak#	Ret. Time	Area	Height	Conc.	Unit	Mark	Name
1	1.042	124585	13541	53.107		M	
2	1.600	40872	4338	17.422		M	
3	4.667	31802	4960	13.556		M	
4	5.000	37335	3936	15.915		M	
Total		234594	26775				

AD2

Peak#	Ret. Time	Area	Height	Conc.	Unit	Mark	Name
1	1.242	6127	480	0.653		M	
2	4.642	3640	397	0.388		M	
3	5.017	928338	126846	98.959		M	
Total		938105	127723				

Figure S30: radioHPLC of pentixafor Validation #3 at 4-hr timepoint

<Sample Information>

Sample Name	: 110123_dose_4hr_20mcl_inj		
Sample ID	: 110123_dose_4hr_20mcl_inj		
Data Filename	: 110123_dose_4hr_20mcl_inj_0-70B-1minHold-254nm_002.lcd		
Method Filename	: 0-70B-1minHold-254nm.lcm		
Batch Filename	: batch_003.lcb		
Vial #	: 1-1	Sample Type	: Unknown
Injection Volume	: 10 uL		
Date Acquired	: 11/1/2023 1:21:35 PM	Acquired by	: System Administrator
Date Processed	: 11/1/2023 1:55:54 PM	Processed by	: System Administrator

<Chromatogram>

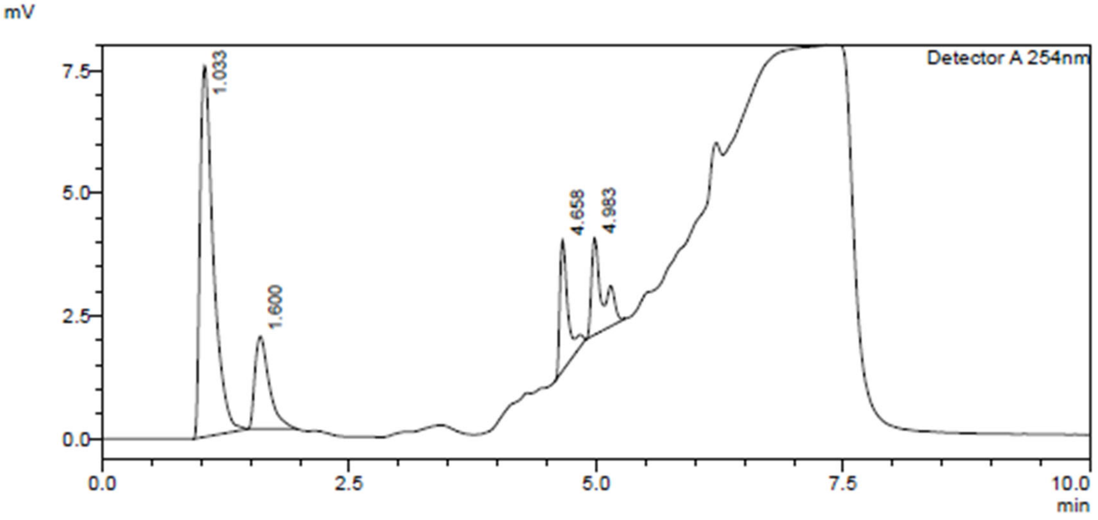

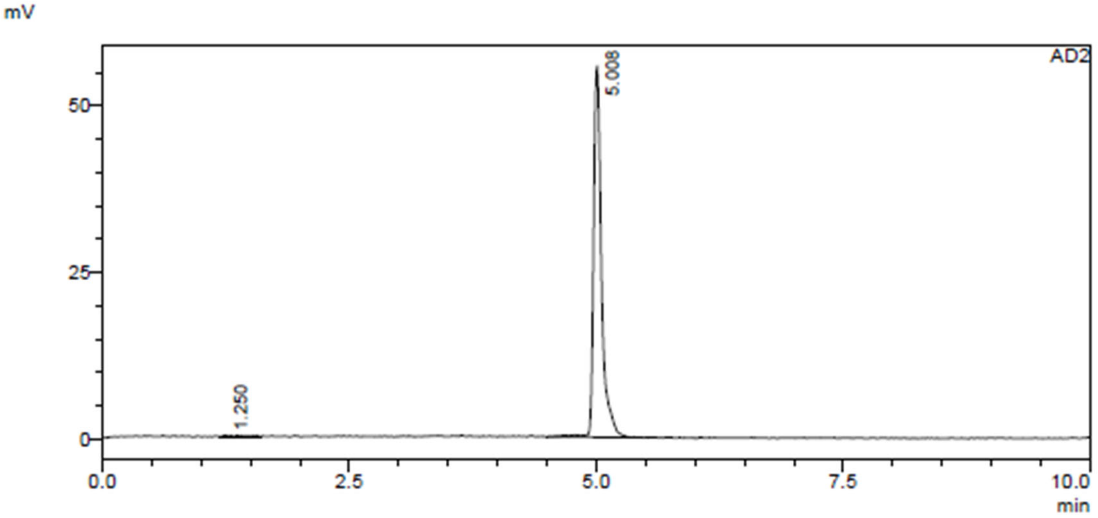

<Peak Table>

Detector A 254nm

Peak#	Ret. Time	Area	Height	Conc.	Unit	Mark	Name
1	1.033	71428	7564	59.031		M	
2	1.600	19123	1889	15.804		M	
3	4.658	15164	2648	12.532		M	
4	4.983	15286	1965	12.633		M	
Total		121001	14066				

AD2

Peak#	Ret. Time	Area	Height	Conc.	Unit	Mark	Name
1	1.250	2202	147	0.718		M	
2	5.008	304504	55685	99.282		M	
Total		306706	55832				

Figure S31: radioTLC of PSMA-11 Validation #1 at initial timepoint

Method: IEW File: 110923PSMA-IN.R001A

Instrument Parameters

Method:	IEW	File:	110923PSMA-IN.R001A
Evaluated:	09 Nov 2023 11:18:26	Created:	09 Nov 2023 10:15:34
Evaluation by:	iew		
Collimator Type:	Hi Efficiency	Width:	10 mm
Elect. Resol:	Normal	Amp. Range:	50 - 2047
Resolution:	256 chan	Chan Size:	0.864 mm
Hi Voltage:	1525 Volts	Chan of Zero mm:	10.3
Run Time:	1.00 min	Max Count:	9999999
Relative Pos:	0.0 mm		

Comments

Default Method for use in defining other methods

Analysis Parameters

Bkg Subtraction:	none	Origin:	60.0 mm
Normalization:	none	Front:	115.0 mm
Total Counts:	126185.0 (126185.0 CPM)	Region:	50.0 - 140.0 mm
Total File Counts:	126185		

Region Analysis

Definition: Table

Reg	(mm) Start	(mm) Stop	(mm) Centroid	RF	Region Counts	Region CPM	% of Total	% of ROI
Rgn 1	52.4	72.3	63.4	0.062	790.0	790.0	0.63	0.63
Rgn 2	71.4	138.8	102.7	0.776	124987.0	124987.0	99.05	99.37
2 Peaks					125777.0	125777.0	99.68	100.00

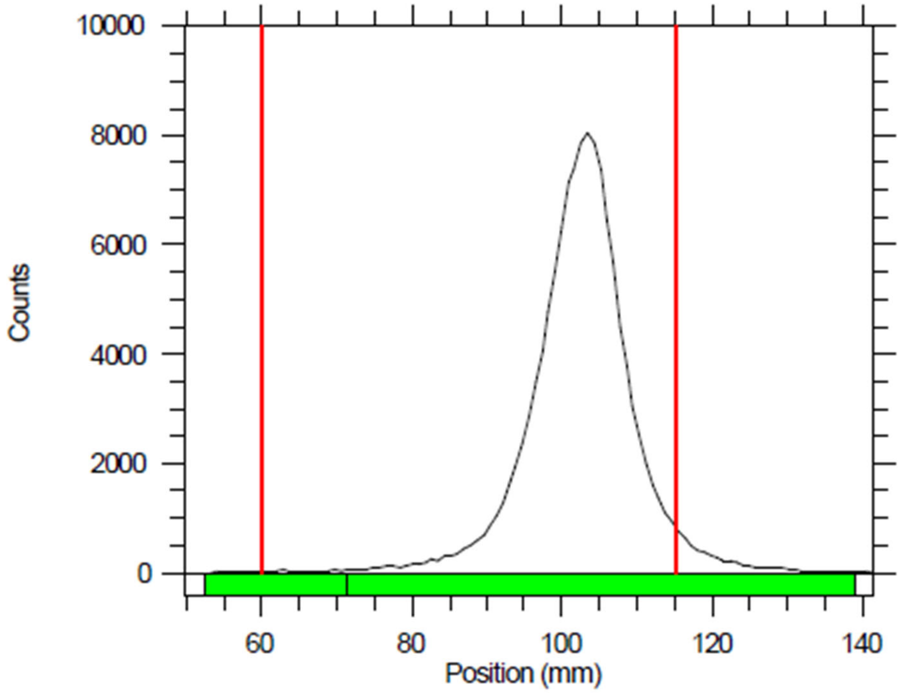

Figure S32: radioTLC of PSMA-11 pseudo-validation #1 at initial timepoint

Method: IEW File: 112023PSMA1-IN.R001A

Instrument Parameters

Method:	IEW	File:	112023PSMA1-IN.R001A
Evaluated:	20 Nov 2023 10:25:52	Created:	20 Nov 2023 10:23:35
Evaluation by:	IEW		
Collimator Type:	Hi Efficiency	Width:	10 mm
Elect. Resol:	Normal	Amp. Range:	50 - 2047
Resolution:	256 chan	Chan Size:	0.864 mm
Hi Voltage:	1525 Volts	Chan of Zero mm:	10.3
Run Time:	1.00 min	Max Count	9999999
Relative Pos:	0.0 mm		

Comments

Default Method for use in defining other methods

Analysis Parameters

Bkg Subtraction:	none	Origin:	60.0 mm
Normalization:	none	Front:	115.0 mm
Total Counts:	110102.0 (110102.0 CPM)	Region:	50.0 - 140.0 mm
Total File Counts:	110102		

Region Analysis

Definition: Table

Reg	(mm) Start	(mm) Stop	(mm) Centroid	RF	Region Counts	Region CPM	% of Total	% of ROI
Rgn 1	43.8	66.2	57.1	-0.052	1204.0	1204.0	1.09	1.10
Rgn 2	66.2	137.9	101.6	0.756	108523.0	108523.0	98.57	98.90
2 Peaks					109727.0	109727.0	99.66	100.00

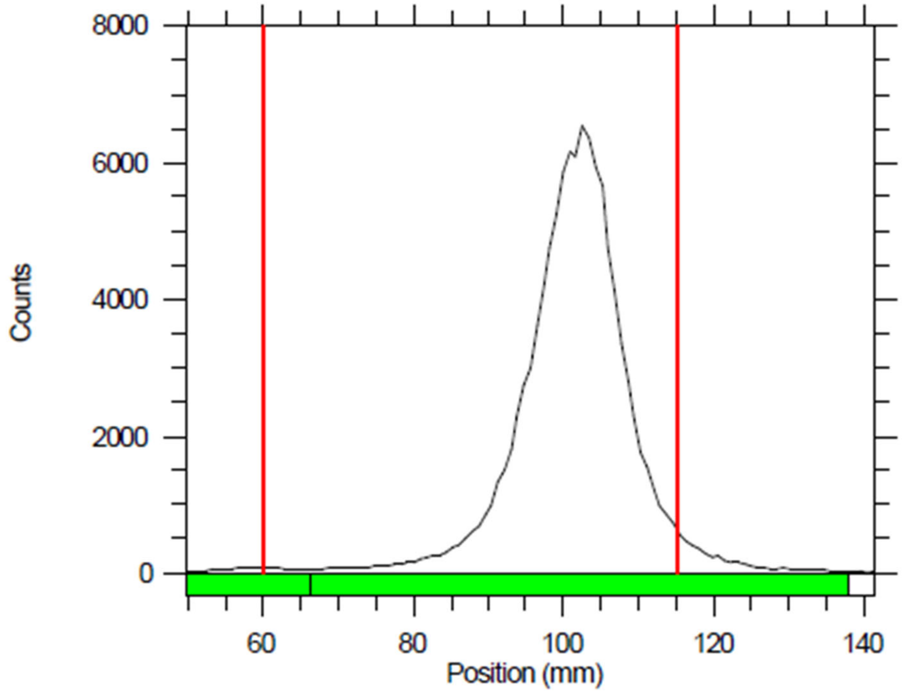

Figure S33: radioTLC of PSMA-11 pseudo-validation #2 at initial timepoint

Method: IEW File: 112123PSAM-INT.R001A

Instrument Parameters

Method:	IEW	File:	112123PSAM-INT.R001A
Evaluated:	21 Nov 2023 10:02:16	Created:	21 Nov 2023 09:58:58
Evaluation by:	IEW		
Collimator Type:	Hi Efficiency	Width:	10 mm
Elect. Resol:	Normal	Amp. Range:	50 - 2047
Resolution:	256 chan	Chan Size:	0.864 mm
Hi Voltage:	1527 Volts	Chan of Zero mm:	10.3
Run Time:	1.00 min	Max Count	9999999
Relative Pos:	0.0 mm		

Comments

Default Method for use in defining other methods

Analysis Parameters

Bkg Subtraction:	none	Origin:	60.0 mm
Normalization:	none	Front:	115.0 mm
Total Counts:	97363.0 (97363.0 CPM)	Region:	50.0 - 140.0 mm
Total File Counts:	97363		

Region Analysis

Definition: Table

Reg	(mm) Start	(mm) Stop	(mm) Centroid	RF	Region Counts	Region CPM	% of Total	% of ROI
Rgn 1	53.3	74.9	64.9	0.089	584.0	584.0	0.60	0.60
Rgn 2	74.9	140.5	106.6	0.847	96374.0	96374.0	98.98	99.40
2 Peaks					96958.0	96958.0	99.58	100.00

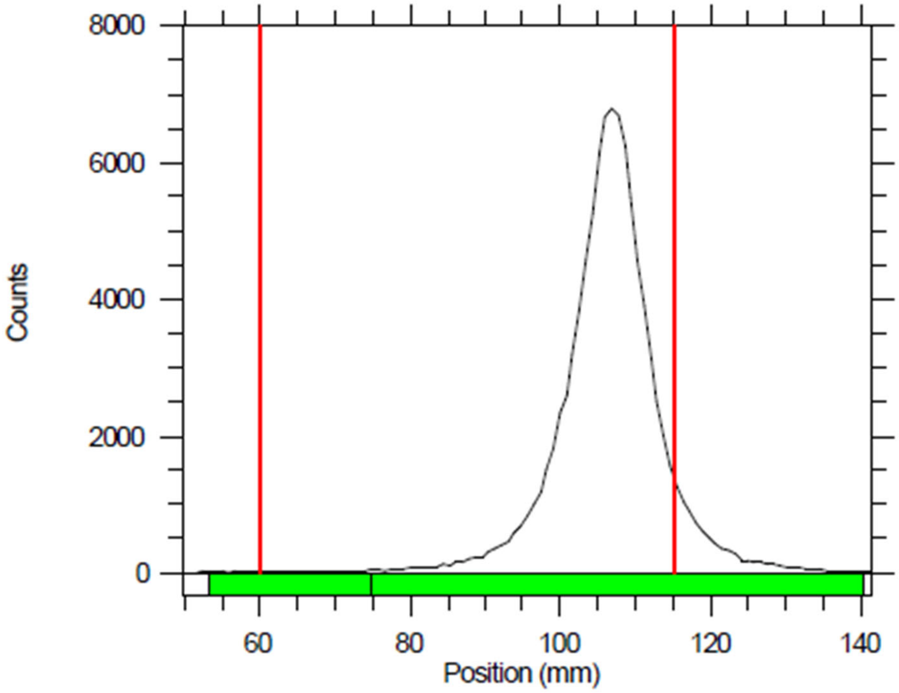

Figure S34: radioHPLC of PSMA-11 Validation #1 at initial timepoint

SHIMADZU LabSolutions Analysis Report

<Sample Information>

Sample Name	: 110923_dose		
Sample ID	: 110923_dose		
Data Filename	: 110923_dose_0-70B-1minHold-254nm_003.lcd		
Method Filename	: 0-70B-1minHold-254nm.lcm		
Batch Filename	: batch_001.lcb		
Vial #	: 1-2	Sample Type	: Unknown
Injection Volume	: 10 uL		
Date Acquired	: 11/9/2023 11:10:27 AM	Acquired by	: System Administrator
Date Processed	: 11/9/2023 11:25:53 AM	Processed by	: System Administrator

<Chromatogram>

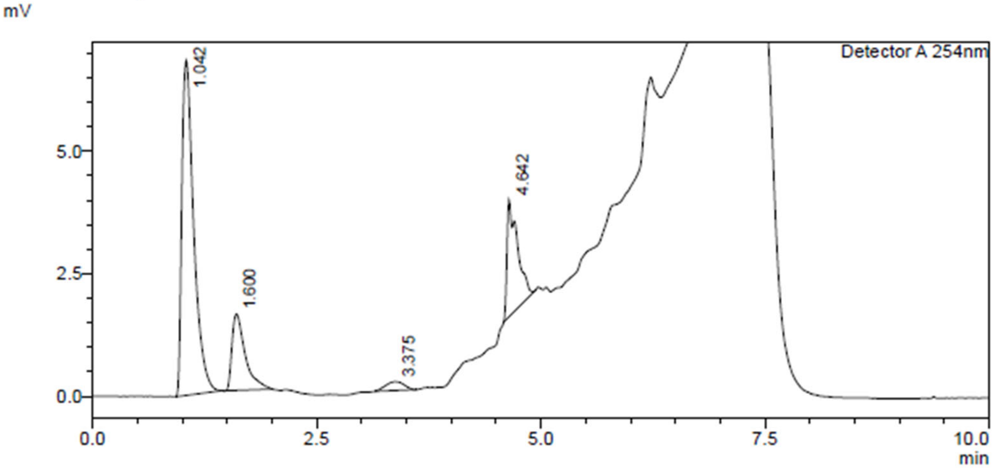

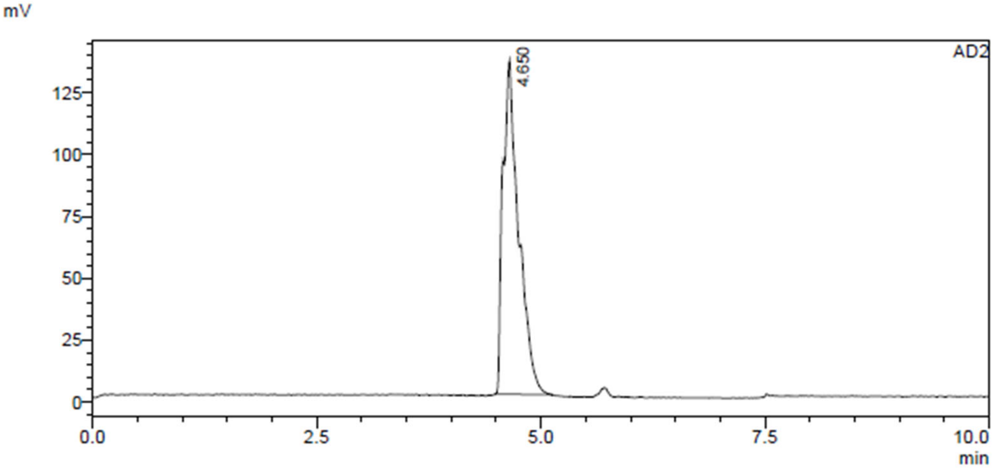

<Peak Table>

Detector A 254nm

Peak#	Ret. Time	Area	Height	Conc.	Unit	Mark	Name
1	1.042	63265	6831	62.391		M	
2	1.600	16115	1557	15.892		M	
3	3.375	2584	181	2.548		M	
4	4.642	19437	2391	19.168		M	
Total		101402	10959				

AD2

Peak#	Ret. Time	Area	Height	Conc.	Unit	Mark	Name
1	4.650	1650577	134943	100.000		M	
Total		1650577	134943				

Figure S35: radioHPLC of PSMA-11 pseudo-validation #1 at initial timepoint

SHIMADZU LabSolutions **Analysis Report**

<Sample Information>

Sample Name	: dose_int_10mcl_inj		
Sample ID	: dose_int_10mcl_inj		
Data Filename	: dose_int_10mcl_inj_0-70B-1minHold-254nm_001.lcd		
Method Filename	: 0-70B-1minHold-254nm.lcm		
Batch Filename	: batch_001.lcb		
Vial #	: 1-2	Sample Type	: Unknown
Injection Volume	: 10 uL		
Date Acquired	: 11/20/2023 10:18:01 AM	Acquired by	: System Administrator
Date Processed	: 11/20/2023 10:31:09 AM	Processed by	: System Administrator

<Chromatogram>

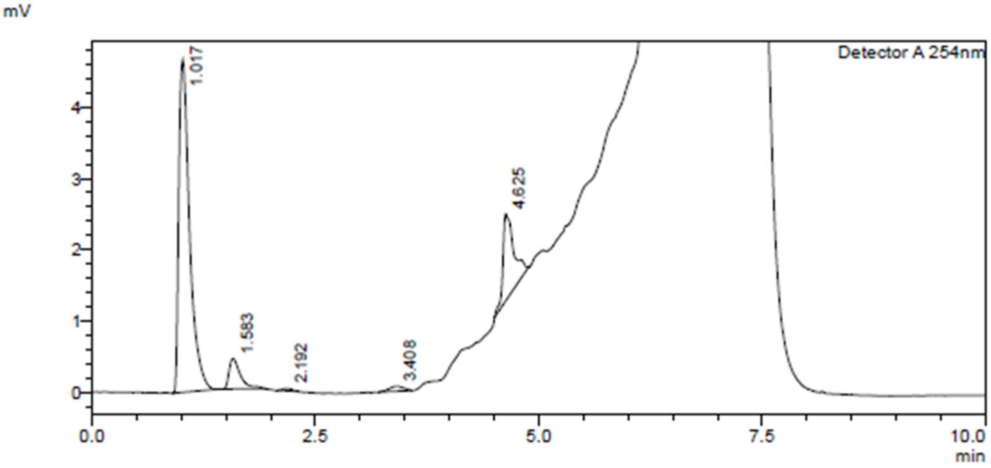

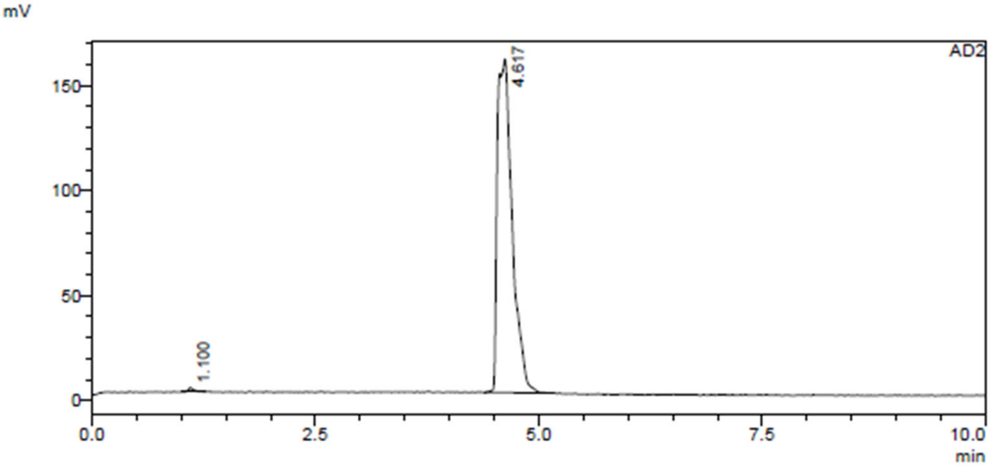

<Peak Table>

Detector A 254nm

Peak#	Ret. Time	Area	Height	Conc.	Unit	Mark	Name
1	1.017	39411	4671	72.499		M	
2	1.583	3785	427	6.963		M	
3	2.192	246	34	0.453		M	
4	3.408	797	69	1.466		M	
5	4.625	10122	1228	18.619		M	
Total		54361	6428				

AD2

Peak#	Ret. Time	Area	Height	Conc.	Unit	Mark	Name
1	1.100	8691	2024	0.474		M	
2	4.617	1824304	158611	99.526			
Total		1832995	160635				

Figure S36: radioHPLC of PSMA-11 pseudo-validation #2 at initial timepoint

SHIMADZU LabSolutions **Analysis Report**

<Sample Information>

Sample Name	: dose_int_10mcl_inj		
Sample ID	: dose_int_10mcl_inj		
Data Filename	: dose_int_10mcl_inj_0-70B-1minHold-254nm_001.lcd		
Method Filename	: 0-70B-1minHold-254nm.lcm		
Batch Filename	: batch_001.lcb		
Vial #	: 1-2	Sample Type	: Unknown
Injection Volume	: 10 uL		
Date Acquired	: 11/21/2023 9:46:54 AM	Acquired by	: System Administrator
Date Processed	: 11/21/2023 10:18:53 AM	Processed by	: System Administrator

<Chromatogram>

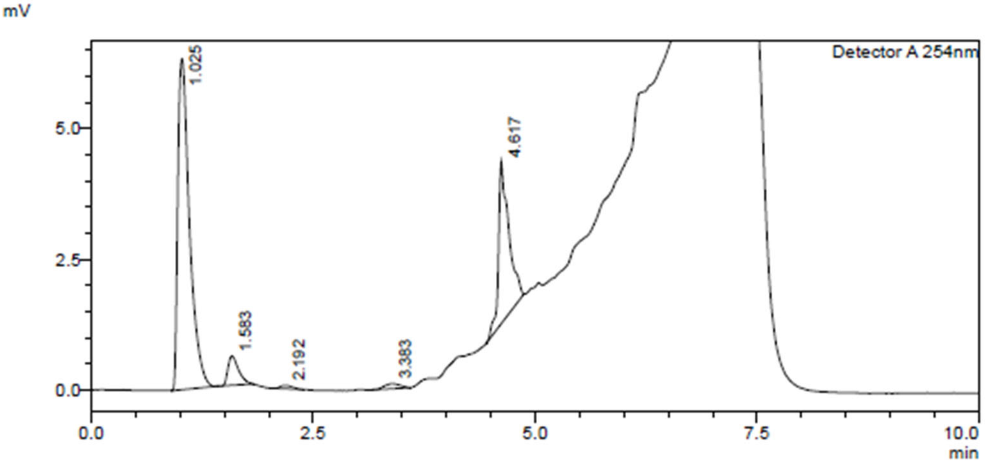

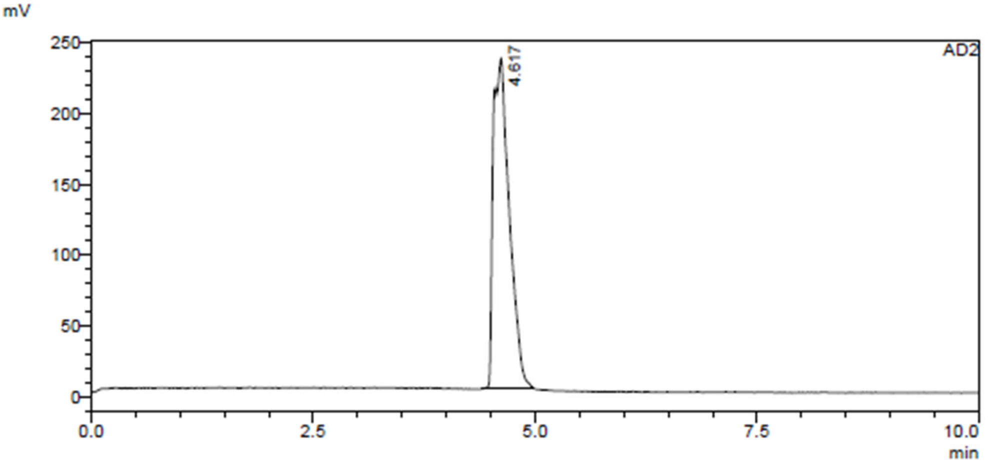

<Peak Table>

Detector A 254nm

Peak#	Ret. Time	Area	Height	Conc.	Unit	Mark	Name
1	1.025	58242	6330	64.774		M	
2	1.583	4632	558	5.151		M	
3	2.192	606	67	0.674		M	
4	3.383	1152	94	1.282		M	
5	4.617	25284	3103	28.119		M	
Total		89916	10152				

AD2

Peak#	Ret. Time	Area	Height	Conc.	Unit	Mark	Name
1	4.617	2941122	233266	100.000		M	
Total		2941122	233266				

Figure S37: radioTLC of PSMA-11 Validation #1 at 4-hr timepoint

Method: IEW File: 110923PSMA-4HR.R001A

Instrument Parameters

Method:	IEW	File:	110923PSMA-4HR.R001A
Evaluated:	09 Nov 2023 13:59:42	Created:	09 Nov 2023 13:56:11
Evaluation by:	TJD		
Collimator Type:	Hi Efficiency	Width:	10 mm
Elect. Resol:	Normal	Amp. Range:	50 - 2047
Resolution:	256 chan	Chan Size:	0.864 mm
Hi Voltage:	1526 Volts	Chan of Zero mm:	10.3
Run Time:	1.00 min	Max Count	9999999
Relative Pos:	0.0 mm		

Comments

Default Method for use in defining other methods

Analysis Parameters

Bkg Subtraction:	none	Origin:	60.0 mm
Normalization:	none	Front:	115.0 mm
Total Counts:	129981.0 (129981.0 CPM)	Region:	50.0 - 140.0 mm
Total File Counts:	129981		

Region Analysis

Definition: Table

Reg	(mm) Start	(mm) Stop	(mm) Centroid	RF	Region Counts	Region CPM	% of Total	% of ROI
Rgn 1	51.5	73.1	63.9	0.071	1265.0	1265.0	0.97	0.98
Rgn 2	73.1	128.4	101.2	0.750	128288.0	128288.0	98.70	99.02
2 Peaks					129553.0	129553.0	99.67	100.00

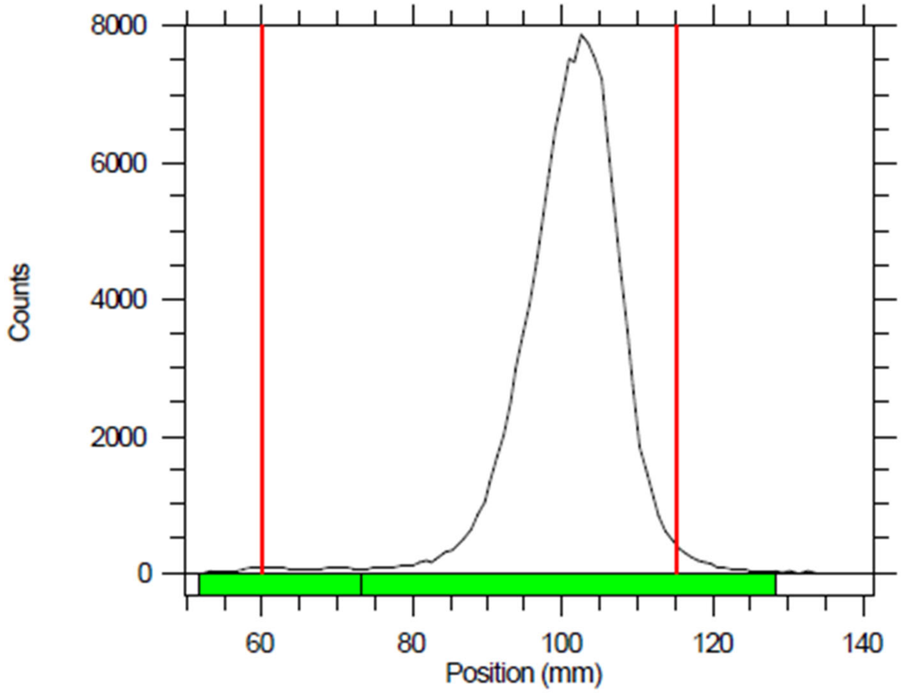

Figure S38: radioTLC of PSMA-11 pseudo-validation #1 at 4-hr timepoint

Method: IEW File: 112023PSAM-4H.R001A

Instrument Parameters

Method:	IEW	File:	112023PSAM-4H.R001A
Evaluated:	20 Nov 2023 13:58:14	Created:	20 Nov 2023 13:56:39
Evaluation by:	IEW		
Collimator Type:	Hi Efficiency	Width:	10 mm
Elect. Resol:	Normal	Amp. Range:	50 - 2047
Resolution:	256 chan	Chan Size:	0.864 mm
Hi Voltage:	1527 Volts	Chan of Zero mm:	10.3
Run Time:	1.00 min	Max Count:	9999999
Relative Pos:	0.0 mm		

Comments

Default Method for use in defining other methods

Analysis Parameters

Bkg Subtraction:	none	Origin:	60.0 mm
Normalization:	none	Front:	115.0 mm
Total Counts:	104728.0 (104728.0 CPM)	Region:	50.0 - 140.0 mm
Total File Counts:	104728		

Region Analysis

Definition: Table

Reg	(mm) Start	(mm) Stop	(mm) Centroid	RF	Region Counts	Region CPM	% of Total	% of ROI
Rgn 1	52.4	73.1	61.9	0.034	956.0	956.0	0.91	0.91
Rgn 2	73.1	126.7	100.3	0.733	103559.0	103559.0	98.88	99.09
2 Peaks					104515.0	104515.0	99.80	100.00

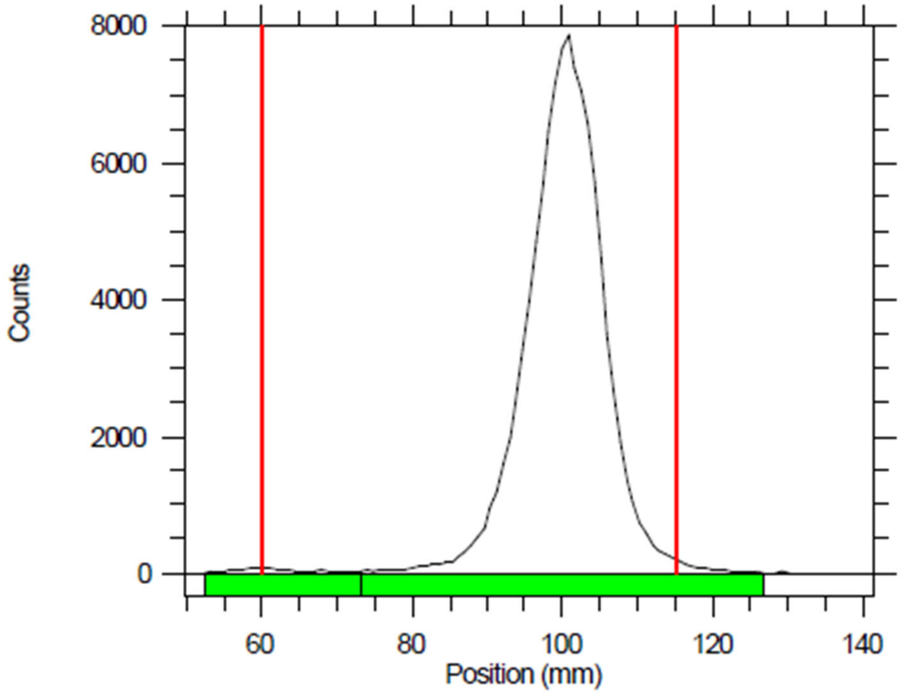

Figure S39: radioTLC of PSMA-11 pseudo-validation #2 at 4-hr timepoint

Method: IEW File: 112123PSMA-4H.R001A

Instrument Parameters

Method:	IEW	File:	112123PSMA-4H.R001A
Evaluated:	21 Nov 2023 13:51:49	Created:	21 Nov 2023 13:50:15
Evaluation by:	IEW		
Collimator Type:	Hi Efficiency	Width:	10 mm
Elect. Resol:	Normal	Amp. Range:	50 - 2047
Resolution:	256 chan	Chan Size:	0.864 mm
Hi Voltage:	1526 Volts	Chan of Zero mm:	10.3
Run Time:	1.00 min	Max Count:	9999999
Relative Pos:	0.0 mm		

Comments

Default Method for use in defining other methods

Analysis Parameters

Bkg Subtraction:	none	Origin:	60.0 mm
Normalization:	none	Front:	115.0 mm
Total Counts:	116788.0 (116788.0 CPM)	Region:	50.0 - 140.0 mm
Total File Counts:	116788		

Region Analysis

Definition: Table

Reg	(mm) Start	(mm) Stop	(mm) Centroid	RF	Region Counts	Region CPM	% of Total	% of ROI
Rgn 1	53.3	79.2	66.8	0.124	901.0	901.0	0.77	0.77
Rgn 2	79.2	125.0	102.0	0.764	115540.0	115540.0	98.93	99.23
2 Peaks					116441.0	116441.0	99.70	100.00

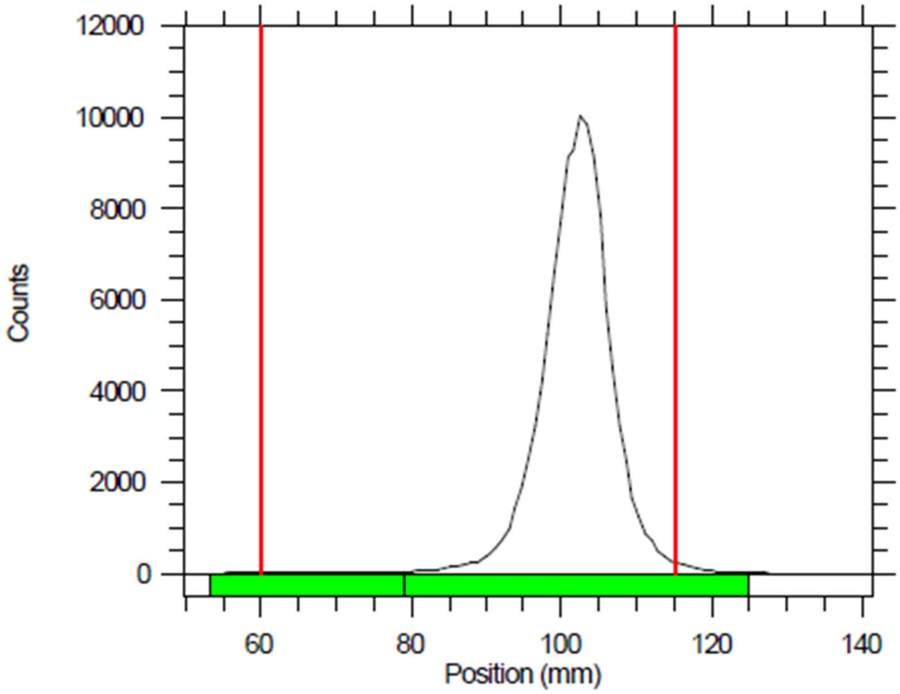

Figure S40: radioHPLC of PSMA-11 Validation #1 at 4-hr timepoint

SHIMADZU LabSolutions **Analysis Report**

<Sample Information>

Sample Name	: 110923_dose_4hr_40mcl_inj		
Sample ID	: 110923_dose_4hr_40mcl_inj		
Data Filename	: 110923_dose_4hr_40mcl_inj_0-70B-1minHold-254nm_002.lcd		
Method Filename	: 0-70B-1minHold-254nm.lcm		
Batch Filename	: batch_003.lcb		
Vial #	: 1-1	Sample Type	: Unknown
Injection Volume	: 40 uL		
Date Acquired	: 11/9/2023 1:56:29 PM	Acquired by	: System Administrator
Date Processed	: 11/9/2023 2:08:13 PM	Processed by	: System Administrator

<Chromatogram>

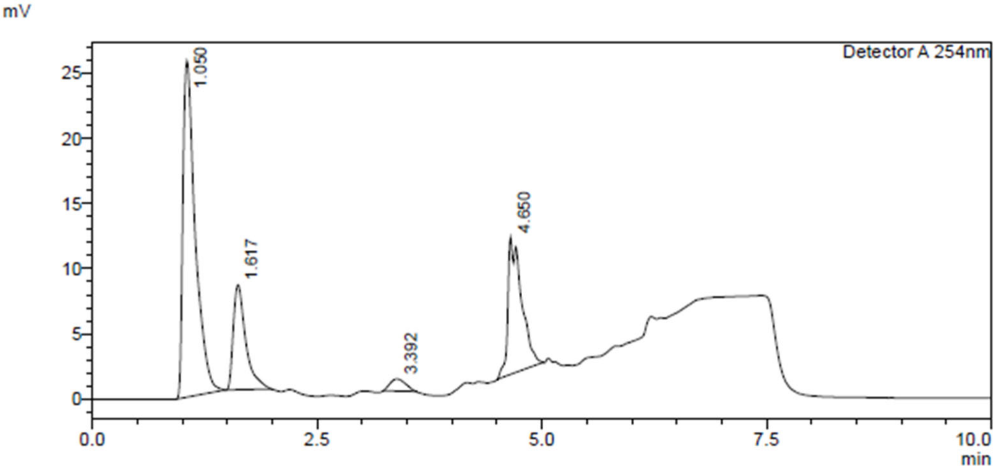

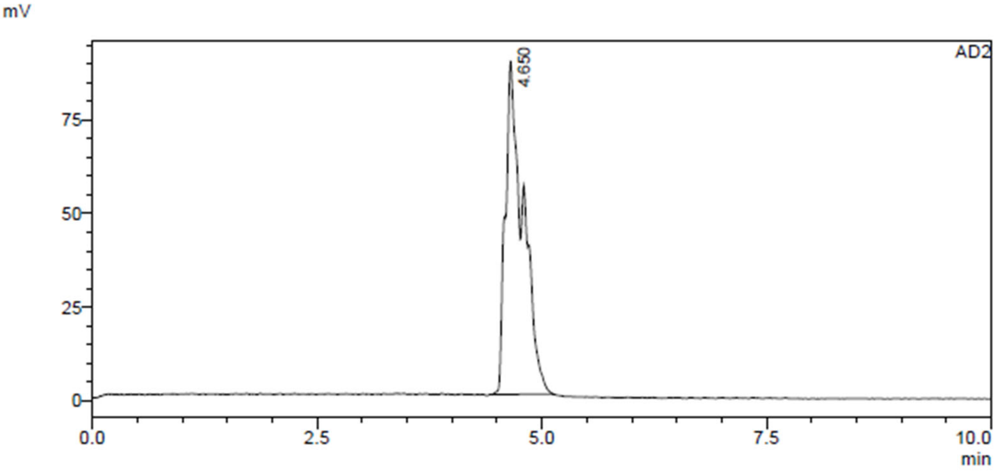

<Peak Table>

Detector A 254nm

Peak#	Ret. Time	Area	Height	Conc.	Unit	Mark	Name
1	1.050	246281	25749	55.211		M	
2	1.617	77687	8029	17.416		M	
3	3.392	11285	922	2.530		M	
4	4.650	110819	10486	24.843		M	
Total		446071	45186				

AD2

Peak#	Ret. Time	Area	Height	Conc.	Unit	Mark	Name
1	4.650	1228351	89268	100.000		M	
Total		1228351	89268				

Figure S41: radioHPLC of PSMA-11 pseudo-validation #1 at 4-hr timepoint

SHIMADZU LabSolutions

Analysis Report

<Sample Information>

Sample Name	: dose_4hr_30mcl_inj		
Sample ID	: dose_4hr_30mcl_inj		
Data Filename	: dose_4hr_30mcl_inj_0-70B-1minHold-254nm_002.lcd		
Method Filename	: 0-70B-1minHold-254nm.lcm		
Batch Filename	: batch_002.lcb		
Vial #	: 1-1	Sample Type	: Unknown
Injection Volume	: 30 uL		
Date Acquired	: 11/20/2023 1:59:32 PM	Acquired by	: System Administrator
Date Processed	: 11/20/2023 2:17:23 PM	Processed by	: System Administrator

<Chromatogram>

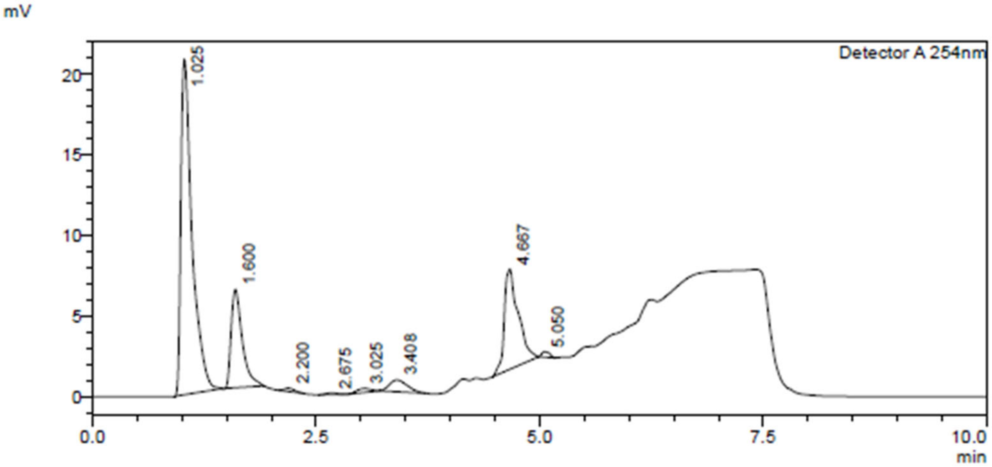

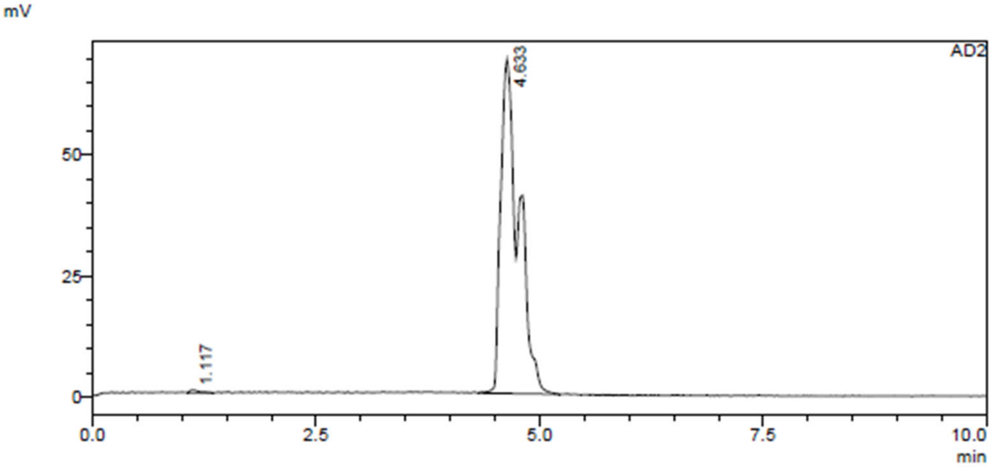

<Peak Table>

Detector A 254nm

Peak#	Ret. Time	Area	Height	Conc.	Unit	Mark	Name
1	1.025	182388	20793	56.933		M	
2	1.600	53082	6087	16.570		M	
3	2.200	1686	207	0.526		M	
4	2.675	646	75	0.202		M	
5	3.025	2604	247	0.813		M	
6	3.408	10171	725	3.175		M	
7	4.667	67785	6217	21.160		M	
8	5.050	1991	332	0.621		M	
Total		320354	34683				

AD2

Peak#	Ret. Time	Area	Height	Conc.	Unit	Mark	Name
1	1.117	4651	548	0.475		M	
2	4.633	974099	69023	99.525		M	
Total		978750	69572				

Figure S42: radioHPLC of PSMA-11 pseudo-validation #2 at 4-hr timepoint

SHIMADZU LabSolutions **Analysis Report**

<Sample Information>

Sample Name : dose_4hr_30mcl_inj
Sample ID : dose_4hr_30mcl_inj
Data Filename : dose_4hr_30mcl_inj_0-70B-1minHold-254nm_001.lcd
Method Filename : 0-70B-1minHold-254nm.lcm
Batch Filename : batch_002.lcb
Vial # : 1-1 Sample Type : Unknown
Injection Volume : 30 uL
Date Acquired : 11/21/2023 1:46:21 PM Acquired by : System Administrator
Date Processed : 11/21/2023 1:58:12 PM Processed by : System Administrator

<Chromatogram>

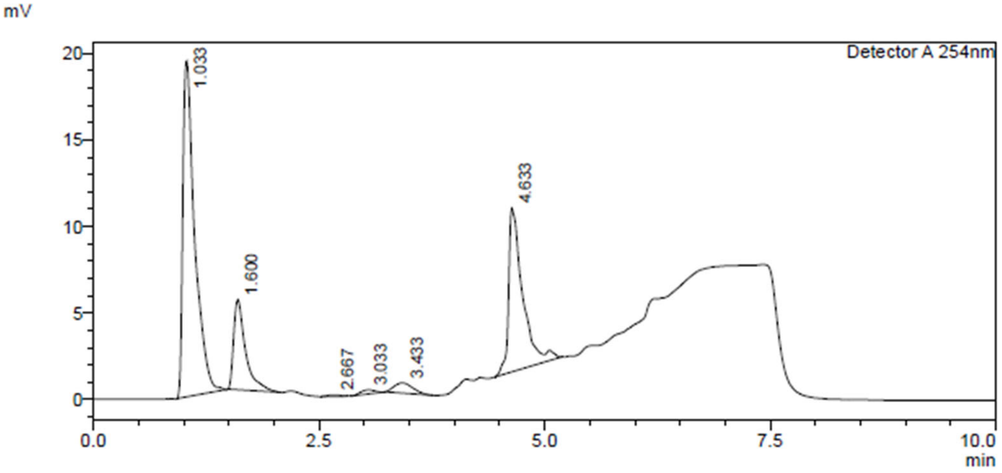

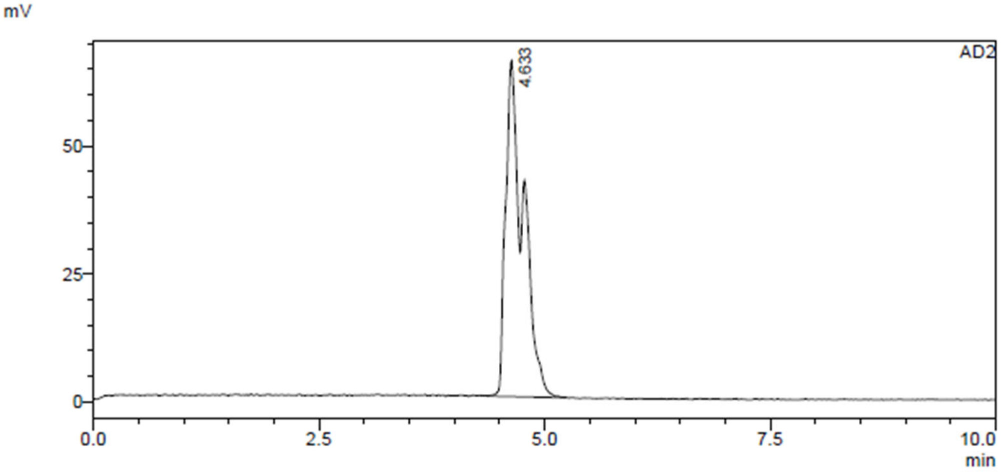

<Peak Table>

Detector A 254nm

Peak#	Ret. Time	Area	Height	Conc.	Unit	Mark	Name
1	1.033	178252	19420	52.229		M	
2	1.600	47890	5213	14.032		M	
3	2.667	648	71	0.190		M	
4	3.033	2548	240	0.747		M	
5	3.433	8712	585	2.553		M	
6	4.633	103237	9469	30.249		M	
Total		341286	34998				

AD2

Peak#	Ret. Time	Area	Height	Conc.	Unit	Mark	Name
1	4.633	899967	65654	100.000			
Total		899967	65654				
